# Short-term effects of pet acquisition and loss on well-being in an unbiased sample during the COVID-19 pandemic

**DOI:** 10.1038/s41598-025-06987-7

**Published:** 2025-07-01

**Authors:** Judit Mokos, Eniko Kubinyi, Dorottya J. Ujfalussy, Ivaylo B. Iotchev, Borbála Paksi, Zsolt Demetrovics, Róbert Urbán, Ádám Miklósi

**Affiliations:** 1 MTA-ELTE Lendület “Momentum” Companion Animal Research Group, Budapest, Hungary; 2 Department of Ethology, ELTE Eötvös Loránd University, Budapest, Hungary; 3 ELTE-NAP Dog Brain Research Group, Budapest, Hungary; 4 Institute of Education, ELTE Eötvös Loránd University, Budapest, Hungary; 5 Institute of Psychology, ELTE Eötvös Loránd University, Budapest, Hungary; 6 College of Education, Psychology and Social Work, Flinders University Institute for Mental Health and Wellbeing, Flinders University, Bedford Park, South Australia Australia; 7 Centre of Excellence in Responsible Gaming, University of Gibraltar, Gibraltar, Gibraltar

**Keywords:** Human behaviour, Quality of life

## Abstract

Research on the impact of pet ownership on well-being has produced mixed results, often influenced by a focus on pet enthusiasts. To address this bias, we conducted a longitudinal study in Hungary using a stratified random sample based on gender, age, education, and settlement type. Unlike previous studies, our participants were not particularly devoted to pets and were not necessarily the primary caretakers, offering a more balanced perspective on how pet acquisition affects well-being. Among the 2783 respondents who participated three times in data collection, 65 acquired a pet, and 75 lost a pet during the COVID-19 pandemic. Pet acquisition initially increased cheerfulness, but this effect was short-lived, lasting only 1–4 months. Over a longer period (up to 6 months), pet acquisition—particularly dog acquisition—was linked to declines in calmness, activity, cheerfulness, and life satisfaction. Importantly, neither mental nor physical well-being was linked to future pet acquisition, and losing a pet had no significant effect on well-being. These findings challenge the widely held belief that pet acquisition leads to lasting improvements in well-being, suggesting instead that the demands of pet care—especially for dogs—can outweigh initial benefits. Moreover, the results underscore the context-dependent nature of the human-animal bond. The stress and uncertainty of a global crisis may alter the typical emotional and psychological benefits of pet ownership.

**Supplementary Information:**

The online version contains supplementary material available at 10.1038/s41598-025-06987-7.

## Introduction

Studies on the effects of pet ownership on well-being have produced mixed findings, which can be attributed to several methodological issues^1–5^. One key limitation is that most studies rely on cross-sectional designs, comparing pet owners to non-pet owners. However, this approach is problematic because differences between pet owners and non-pet owners may extend beyond ownership itself, as pet owners often have better socioeconomic status, which is linked to improved mental and physical well-being^6^. In fact, the positive effects of pet ownership often disappear after controlling for such confounders^6–8^. Another source of mixed results is the inconsistent operationalization of pet ownership; many studies fail to clearly define how pet ownership was assessed^9^. To accurately evaluate the impact of pet acquisition, a within-subject design is preferable, comparing individuals’ well-being longitudinally, before and after acquiring a pet in the household. Additionally, to distinguish between initial novelty effects and longer-term changes, short-term and long-term effects of pet ownership should be compared. While acquiring a new pet can bring joy, excitement, and positive changes in everyday daily routines^10–12^, maintaining a dependent relationship can become burdensome over time^13–16^ due to behavioural or health problems and restrictions on activities such as traveling or visiting places that do not allow pets.

However, identifying and tracking individuals who are about to acquire a pet is challenging. Previous studies have often relied on convenience samples, such as individuals visiting shelters with the intention of adopting a pet^12,17^. These samples are inherently biased, as adopters are typically motivated by the desire to “rescue” a pet, which may not reflect the motivations of individuals who purchase pets from breeders or acquire them from other sources^18–21^. Furthermore, comparing individuals considering pet adoption to those who have already adopted may be misleading, as the decision to adopt could itself reflect underlying differences in well-being. To assess the long-term effects of pet ownership, it is crucial to measure participants’ well-being before and after adoption and to follow them for at least several months post-adoption.

Research also suggests that the impact of pet ownership on well-being varies by species. Dog owners tend to report higher life satisfaction^22^ and well-being^23,24^ than cat owners, and they have higher self-esteem, than individuals without pets, while cat ownership is associated with lower self-esteem among women^22–25^. Cat owners reported poorer sleep quality^26^lower stroke mortality^27^ but higher mortality from acute coronary syndrome^28^ than dog owners. The greater burdens associated with dog ownership^29^ as well as physical activities and social interactions^30^ may explain some of these differences. Therefore, distinguishing between species is essential for understanding the broader impact of pet ownership on well-being.

In this study, we aimed to examine the effects of pet acquisition on well-being while addressing the methodological shortcomings described above. Relying on a Computer Assisted Web Interview (CAWI) panel that is unbiased in its attitude toward pet ownership, applying both between- and within-subject comparisons, our goals were to (1) assess how pet acquisition or pet loss affects well-being using a longitudinal design, (2) explore differences based on species (dogs, cats, and other animals), and (3) investigate whether well-being predicts future pet acquisition (i.e., whether individuals with higher or lower well-being are more likely to acquire a pet).

Notably, the sample was originally collected for studying the effects of COVID-19 restrictions on the mental health of the Hungarian population^31 ^which represents a limitation because the findings may be specific to times of global or local crises. Lockdowns may have altered the impact of pet ownership, with some studies reporting increased difficulties and anxiety as well as slightly worse mental health among pet owners^32–34 ^while others found smaller decreases in mental health compared to non-owners^35^. A recent review^36^ examining 21 studies on pet ownership and mental health during the COVID-19 pandemic also found contradictory results across measures of anxiety, depression, general well-being, and other mental health symptoms. For example, regarding anxiety, four of eight studies found no significant association with pet ownership, while one reported lower anxiety levels among pet owners. However, subjective reports often indicated that individuals felt their pets were helpful during the pandemic, even when objective measures did not support these perceptions^37^. Animal type did not appear to significantly influence anxiety or affective states in most studies reviewed, but in an international sample, people obtained more comfort from horses and dogs than from other animals^38 ^and, in a Malaysian sample, cat owners reported more positive emotions and greater well-being than dog owners^39^.

Given these inconsistencies, we hypothesised that pet acquisition would lead to both short- and long-term improvements in well-being, consistent with studies emphasising the psychological benefits of human-animal bonds^11,40–42^. Conversely, we expected that pet loss would lead to measurable declines in well-being^43–45^. We also expected that acquiring or losing a dog would have a stronger impact than in the case of a cat or other animals^22–24,29^. However, since previous studies have often relied on convenience samples (participants who are easily accessible and highly interested in pet ownership), it is plausible that acquiring or losing a pet might have no significant effect in a more representative, unbiased sample during a global crisis.

Importantly, in this study, we had no information on the participants’ ownership status, i.e. whether they were the primary caretaker of the animal or not, as acquisition was defined as the acquisition of a new pet by the household (regardless of whether the pet was obtained through adoption, purchase, or any other method), while losing a pet was defined as the situation where participants no longer have a pet in their household (which may occur due to various reasons, including the pet’s death, abandonment, or being given away). The effect of a pet’s appearing or disappearing from a household may differ from the effect of a pet’s presence or absence in the primary caretaker’s life due to the nature of the emotional bond and the level of personal involvement. Therefore, the results of this study reflect the effects of living with an animal rather than those of caring for one during a global crisis.

## Methods

### Ethics

Participation in this study was anonymous and voluntary. The participants were requested to construct a code which made it possible to contact them later for a possible follow-up data-collection. The study was performed in accordance with the Helsinki declaration, and the protocol was approved by the Institutional Review Board of Eötvös Loránd University, Budapest, Hungary (no: 2020/134). Informed consent was obtained from all participants.

### Data collection and subjects

#### Description of the computer-assisted web interviewing (CAWI) panel

The target population was Hungarian residents aged 18 and older, and the sampling frame consisted of members of the CAWI “living panel” (74,500 persons, https://cawi.hu/). The CAWI panel is a pre-recruited online panel run by CAWI services. Its large size and stratification allow it to represent the entire adult population of the country in terms of age, education, type of settlement, and gender. The panel includes 100,000 members, of whom the “living panel” consists of those who were active within 90 days prior to the survey.)

The sample was selected using a one-stage random sampling, stratified by gender, age group (18–29, 30–49, 50–64, 64+), educational level (primary, secondary, tertiary), and type of settlement (capital city, county seat, town, village). Groups with historically low response rates, based on previous panel experience, were over-sampled to improve representation.

The survey was titled “Coronavirus study”, and participants were informed that the aim was to examine the situation arising from the pandemic, understand public perceptions of the pandemic, and explore responses to it. Pet ownership was not mentioned in the introduction.

#### Time of data collection

The longitudinal data collection was conducted in Hungary during the COVID-19 pandemic across three periods: 1 st period: 27 March 2020–6 April 2020, 2nd period: 14 May 2020–26 May 2020, and 3rd period: 22 September 2020–6 October 2020. Therefore, nearly two months elapsed between the 1 st and 2nd periods and four months between the 2nd and 3rd periods.

#### Epidemic management in the three waves

The first case of SARS-CoV-2 in Hungary was reported on 4 March 2020, and the first death was announced on 15 March 2020. By 18 March, it was confirmed that SARS-CoV-2 had spread across the country.

During the 1 st and 2nd data collection periods, the number of daily new infections and active cases remained relatively low (Figure S1). However, strict regulations were introduced, including the closure of kindergartens and schools, and people were instructed to stay at home (Table S1). In contrast, during the 3rd period, the number of active cases and daily infections was high, but the regulations had changed. Instead of advising people to stay at home, they were instructed to wear masks, limit social contact, and continue with their daily routines.

These changing circumstances could have influenced participants’ mental well-being. Therefore, we tested whether average mental well-being differed across the three data collection periods (Table S2). Since significant differences were found, all data were standardised.

#### Standardization

Since well-being changed over time (Table S2), comparing raw scores before and after pet acquisition or loss could be misleading, as any observed differences might reflect general temporal changes rather than the effect of pet acquisition or loss. To account for this, the scores were standardised using the *scale* function from the *base* R package, separately for each of the three periods, based on data from participants who provided data in all three periods (*N* = 2,783).

The standardised scores reflect how a participant’s well-being compares to others (including both pet owners and non-pet owners) within the same period. A score of zero represents the average well-being for that period, positive values represent higher-than-average well-being, and negative values indicate lower-than-average well-being.

#### Subjects

In this study, 2783 participants completed all three data collection periods. The average age of the participants was 53.8 ± 14.2 years, and 60% were women. A total of 1,446 participants (52%) reported living with a pet during at least one of the three data collection periods.

Of the 2,783 households, 65 (2.3%) acquired, and 75 (2.7%) lost a single pet. Participants whose households acquired or lost multiple pets (e.g., both a cat and a dog) were excluded from the analysis. Additionally, 17 participants (0.6%) reported both acquiring and losing a pet during the pandemic. Descriptive data (age, gender, settlement type, and household composition) on the participants are in Table 1.

#### The level of commitment to pet ownership

The “Coronavirus study” survey did not directly ask about pet ownership status, only whether there was a pet in the household. However, ownership status could be indirectly assessed based on the number of people in the household. If a participant lived alone, it can be reasonably assumed that they were the primary caretaker of the pet. A total of 12.3% of participants who acquired a pet and 14.7% of those who lost a pet lived alone (Table 1).

Another way to estimate the relationship with the pet is to assess the level of commitment. The survey included a question about how important the animal was to the participant compared to other close relationships. While no dogs were reported to be the most important relationship, some cats and other animals were. Conversely, some pets were reported as not being important (Table 1).

**Table 1:** Descriptive statistics of participants whose households acquired or lost a pet.

	Acquired a pet (no pet in 1 st period), *N* = 65		Lost a pet (no pet in 3rd period), *N* = 75	
Age (year ± SD)	49.1 ± 15.3		50.6 ± 14.5	
	N (%)			
Gender				
Male	21 (32.3)		30 (40.0)	
Female	44 (67.7)		45 (60.0)	
Settlement type				
1 Capital city (Budapest)	18 (27.7)		13 (17.3)	
2 County seat	30 (46.2)		30 (40.0)	
3 Other city	11 (16.9)		21 28.0)	
4 Village	6 (9.2)		11 (14.7)	
Household composition (Q6)				
Living with other people	57 (87.7)		64 (85.4)	
Living alone	8 (12.3)		11 (14.7)	
Period in 2020	2nd (14–26/05)	3rd (22/09 − 06/10)	1 st (27/03–06/04)	2nd (14–26/05)
Species (Q53)				
Dog	4 (20.0)	17 (37.8)	14 (42.4)	14 (33.3)
Cat	9 (45.0)	19 (42.2)	8 (24.2)	19 (45.2)
Other	7 (35.0)	9 (20.0)	11 (33.3)	9 (21.4)
If you think about your close relationships, how would you describe your current relationship with your dog(s)? (Q54)				
1 Most important	0 (0.0)	0 (0.0)	0 (0.0)	0 (0.0)
2 Important	0 (0.0)	2 (12.5)	2 (14.3)	3 (21.4)
3 Somewhat important	4 (100.0)	12 (75.0)	7 (50.0)	6 (42.9)
4 Not important	0 (0.0)	2 (12.5)	5 (35.7)	5 (35.7)
If you think about your close relationships, how would you describe your current relationship with your cat(s)? (Q58)				
1 Most important	1 (11.1)	5 (27.8)	0 (0.0)	5 (26.3)
2 Important	5 (55.6)	7 (38.9)	5 (71.4)	8 (42.1)
3 Somewhat important	2 (22.2)	6 (33.3)	2 (28.6)	5 (26.3)
4 Not important	1 (11.1)	0 (0.0)	0 (0.0)	1 (5.3)
If you think about your close relationships, how would you describe your current relationship with your companion animal(s) [other than a dog or cat]? (Q62)				
1 Most important	3 (60.0)	1 (11.1)	2 (22.2)	5 (55.6)
2 Important	1 (20.0)	3 (33.3)	6 (66.7)	0 (0.0)
3 Somewhat important	1 (20.0)	3 (33.3)	1 (11.1)	2 (22.2)
4 Not important	0 (0.0)	2 (22.2)	0 (0.0)	2 (22.2)

#### Well-being variables

Participants’ well-being was assessed using nine items. Items 3–7, which constitute the World Health Organization Well-Being Index (WHO-5)^46^ were analysed both individually and as a composite scale, as preliminary analysis confirmed that both approaches revealed meaningful patterns (Table 2).

**Table 2:** Variables used to assess well-being.

*N*	Variable name	Item	Scale (higher number indicates higher levels)
1	Sadness	In the past week, how often have you felt sad, depressed, down and less interested in life?	1–10
2	Anxiety	In the past week, how often have you felt anxious and tense?	1–10
3	Cheerfulness	Over the past week, I have felt cheerful and in good spirits	1–4
4	Calmness	Over the past week, I have felt calm and relaxed spirits	1–4
5	Activity	Over the past week, I have felt active and vigorous	1–4
6	Feeling fresh and rested	Over the past week, I woke up feeling fresh and rested	1–4
7	Having interesting days	Over the past week, my daily life has been filled with things that interest me	1–4
8	WHO-5 modified	Sum of items 3–7 (modification: the questions asked about the past week instead of the past two weeks)	1–20
9	Physical well-being	How would you describe your health?	1–5

### Statistical analysis

#### Does acquiring a pet affect well-being? GLMM A-D

To examine the effect of pet acquisition on well-being, four sets of General Linear Mixed Models (*GLMM sets A*,* B*,* C*, and *D*) were performed. In each model, a well-being variable from a given data collection period was used as the response variable, with participant ID included as a random factor. The analyses were performed using the *lmer* function from the *lme4* package^47^ and p-values were calculated using the *sum* function from the *jtools* package^48^ in R.

*GLMM set A* evaluates the overall effect of pet acquisition on well-being. The independent variables included the data collection period (1st, 2nd, or 3rd), pet acquisition status (whether the participant’s household acquired a pet during any of the three data collection periods), age, sex and settlement type. To assess whether demographic factors moderated the effect of pet acquisition on well-being, interaction terms between acquisition status and age, sex, and settlement type were included. The model is specified as: Y ~ data collection period + acquisition + age + sex + settlement type + acquisition * age + acquisition * sex + acquisition * settlement type + (1|ID), where Y represents a specific well-being variable at a given data collection period.

*GLMM set A* used data from participants who completed all three data collection periods and either acquired a dog (*N* = 21), cat (*N* = 28), or another species of pet (*N* = 16), or did not have pets (*N* = 1,337), resulting in a total sample size of 1,402. For each participant, three data points were used (1st, 2nd and 3rd data collection periods).

*GLMM set B* focused on the effect of both pet acquisition and the species of the acquired pet on well-being. The independent variables included the data collection period (1st, 2nd, and 3rd), species of pet acquired (none, dog, cat or other), whether the participant had a pet in the household at the data collection period (yes or no), age, sex and settlement type. Interaction terms were included between having a pet at the data collection time and the species of the pet acquired, the age, sex and settlement type, to explore whether the demographic background and the species of pet moderated the effect of pet acquisition on well-being. The model was structured as: Y ~ data collection period + species of pet + having a pet at the data collection period + age + sex + settlement type + having a pet at the data collection period * species of pet + having a pet at the data collection period * age + having a pet at the data collection period * sex + having a pet at the data collection period * settlement type + (1|ID), where Y represents a specific well-being variable at a given data collection period.

*GLMM set B* was carried out on the same dataset as *GLMM set A*, with three data points from the participants across the three data collection periods.

*GLMM set C* examined how the species of the acquired pet affects well-being among participants who acquired a pet (*N* = 65). The independent variables included the data collection period (1st, 2nd, 3rd data collection period), species of pet (dog, cat, other), pet ownership status during the given data collection period (not yet acquired or already acquired), age, sex, and settlement type. Interaction terms were used between having a pet at the data collection period and the species of the pet, age, sex, and settlement type, to assess whether the demographic factors and pet species moderated the effect of pet ownership on well-being. The model was structured as: Y ~ data collection period + species of pet + having a pet at the data collection period + age + sex + settlement type + having a pet at the data collection period * species of pet + having a pet at the data collection period * age + having a pet at the data collection period * sex + having a pet at the data collection period * settlement type + (1|ID), where Y represents a specific well-being score at a given data collection period. Three data points were used for each participant across the three data collection periods.

*GLMM set D* focused on comparing well-being before and after pet acquisition. Data were drawn from participants who acquired a pet during the study. “Before acquisition” was defined as the last data collection period in which the participant did not own a pet, while “after acquisition” was defined as the first data collection period in which the participant reported owning a pet. For example, if a participant did not own a pet during the 1 st data collection period but owned one during the 2nd and 3rd periods, “before acquisition” corresponded to the 1 st period and “after acquisition” to the 2nd period. If a participant reported not having a pet at the 1 st and 2nd periods and having a pet at the 3rd period, “before acquisition” corresponded to the 2nd period, and “after acquisition” to the 3rd period. The sample size used in GLMM set D was 63, of whom 20 acquired a pet at the 2nd period, and 43 acquired a pet at the 3rd period. The dependent variable was the standardized score of one of the well-being measurements before or after acquisition. The independent variables were the acquisition status (before or after acquisition), pet species (dog, cat, or other), the period of the acquisition (2nd or 3rd data collection period), age, sex, and settlement type. The model was structured as: Y ~ acquisition state + species of pet + period of acquisition + age + sex + settlement type + acquisition state * pet species + (1|ID), where Y represented one of the well-being scores at a given data collection period. Two data points were used for each participant: one before and one after acquisition.

#### Does losing a pet affect well-being? GLMM E

*GLMM E* examined changes in well-being before and after losing a pet, using data from participant who reported having a pet during the 1 st data collection period but not during the 2nd or 3rd data collection periods, indicating that they had lost their pet between the 1 st and 2nd or between the 2nd and 3rd data collection period. The sample consisted of 75 participants, of whom 28 lost a dog, 27 lost a cat, and 20 lost another species of pet. Each participant contributed to two data points: one from before and one from after pet loss. The “before loss” period was defined as the last data collection period in which the participant reported having a pet, and the “after loss” period was the first data collection period in which they reported no longer having a pet. For instance, if a participant had a pet in the 1 st data collection period, but not in the 2nd and 3rd periods, their well-being data from the 1 st data collection period represented the “before loss” state, and their 2nd -period data represented the “after loss” state, and the data of 3rd period were excluded. Similarly, if a participant reported having a pet in the 1 st and 2nd data collection periods but not in the 3rd data collection period, their 2nd -period data represented the “before loss” state, and their 3rd -period data represented the “after loss” state. The dependent variable was one of the well-being measures, and the independent variables included the pet loss state (before or after losing the pet), species of pet lost, period of pet loss, age, sex and settlement type. Participant ID was included as a random effect. To assess whether the species of pet moderated the effect of pet loss on well-being, interaction terms between the pet loss state and pet species, age, sex, and settlement type were included. The model was structured as: Y ~ pet losing state + species of pet lost + period when pet was lost + age + sex + settlement type + pet losing state * species of pet + (1|ID). Two data points were used for each participant: one from before and one from after losing the pet.

#### Bootstrapping for GLMM A-E

Due to the limited sample size of individuals who acquired or lost a pet, all GLMM analyses (sets A-E) were bootstrapped with case resampling (B = 1000) as well, using the *bootstrap* function from the *lmersampler* R package^49^. Detailed results from these analyses are provided in the Supplementary Material.

#### Does well-being differ between individuals whose household acquired a pet and non-pet owners?

To study whether the well-being differs between participants who are going to acquire a pet and non-pet owners, Kruskal-Wallis tests were conducted, followed by Dunn post hoc tests. Standardized well-being scores were used. First, the 1st -period scores of the participants who acquired a pet during the 2nd data collection period were compared to the 1st-period scores of non-pet owners (sample sizes: participants who acquired a dog = 4, cat = 9, other = 7, non-pet owners = 1337). Second, the 2nd -period scores of the participants who acquired a pet during the 3rd period were compared to the 2nd-period scores of non-pet owners (sample sizes: participants who acquired a dog = 17, cat = 19, other = 9, non-pet owners = 1337).

## Results

### The effect of acquisition and species

**Fig. 1:**
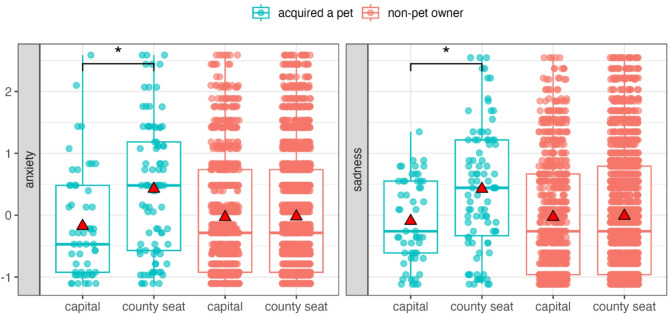
Interaction between pet acquisition and settlement type on anxiety and sadness (GLMM set A). Asterisks indicate significant differences. Data points are shown with transparency and horizontal jitter to reduce overlap. Mean values are represented by red triangles.

Comparing participants who acquired a pet versus non-pet owners revealed no significant overall effect of pet acquisition on well-being (GLMM set A, Figure S2). However, interactions between pet acquisition and settlement type indicated that the effect of acquisition on anxiety (Est. = −0.58, S.E = 0.25, t = −2.34, df = 1393.64, *p* = 0.02) and sadness (Est. = −0.50, S.E = 0.25, t = −2.03, df = 1391.53, *p* = 0.04) was moderated by settlement type, with bootstrapped GLMM results showing 95% confidence intervals (CIs) that did not include zero. While anxiety and sadness levels did not differ by settlement type among non-pet owners, those who acquired a pet and lived in the capital city reported lower anxiety and sadness compared to those living in county seats (Fig. 1).

**Fig. 2:**
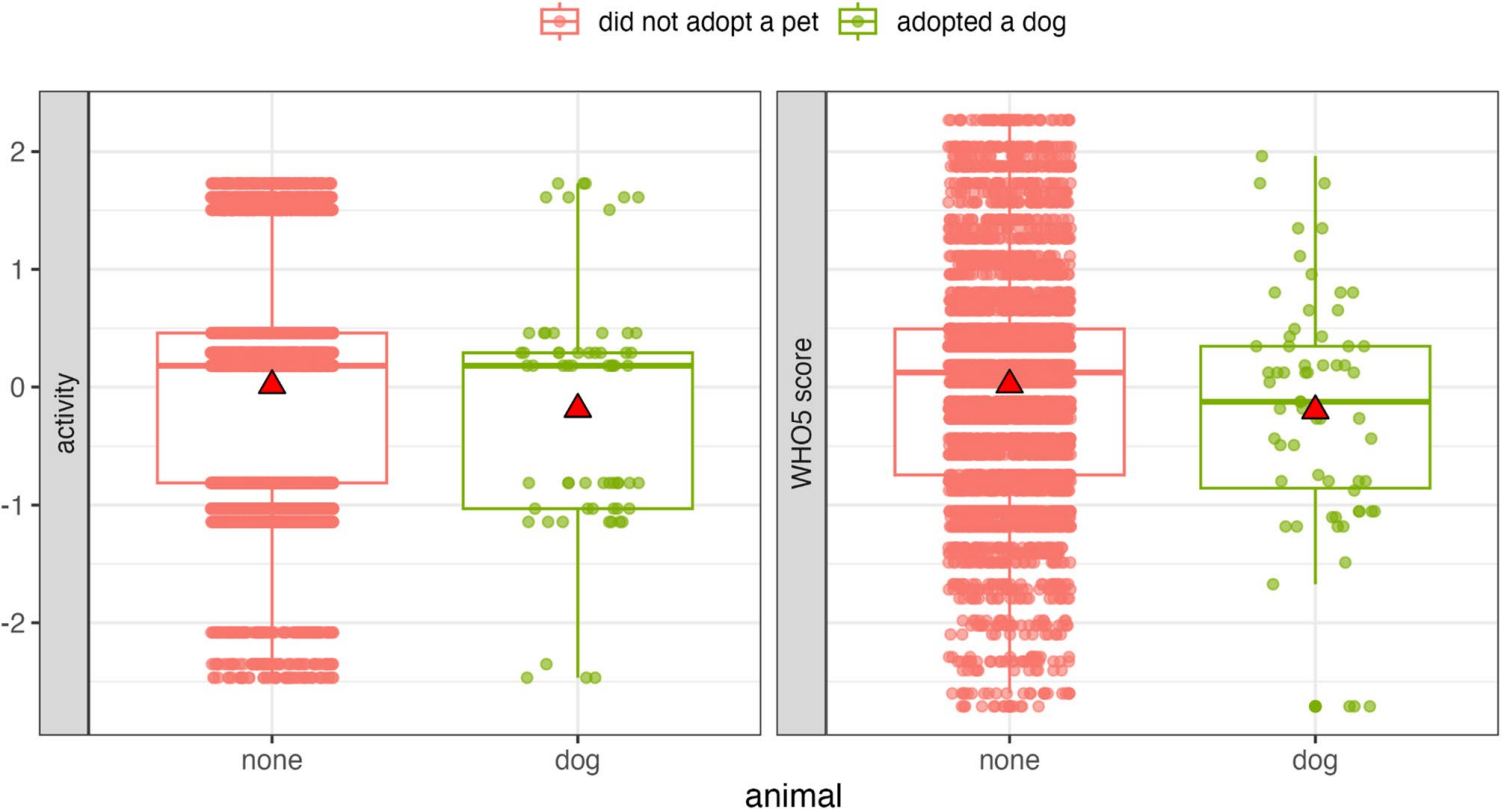
Participants who acquired a dog were less active and had a lower WHO-5 score compared to non-pet owners (main effect of GLMM set B). Data points are displayed with transparency and horizontal jitter to minimise overlap. Mean values are represented by red triangles.

However, when examining the effect of species of pet, participants who acquired a dog were significantly less active (Est. = −0.92, S.E = 0.31, t = −2.94, df = 4151.40, *p* < 0.001) and had an overall lower WHO-5 score (Est. = −0.64, S.E = 0.30, t = −2.17, df = 3990.65, *p* = 0.03) compared to non-pet owners. Although the bootstrapped GLMM showed that the 95% CIs for these effects included zero (i.e., no effect), the 66% CIs did not (i.e., the effect is likely non-zero, albeit with less confidence) suggesting a possible trend toward lower mental well-being in participants who acquired a dog relative to non-pet owners (GLMM set B, Fig. 2, Figure S3).

**Fig. 3:**
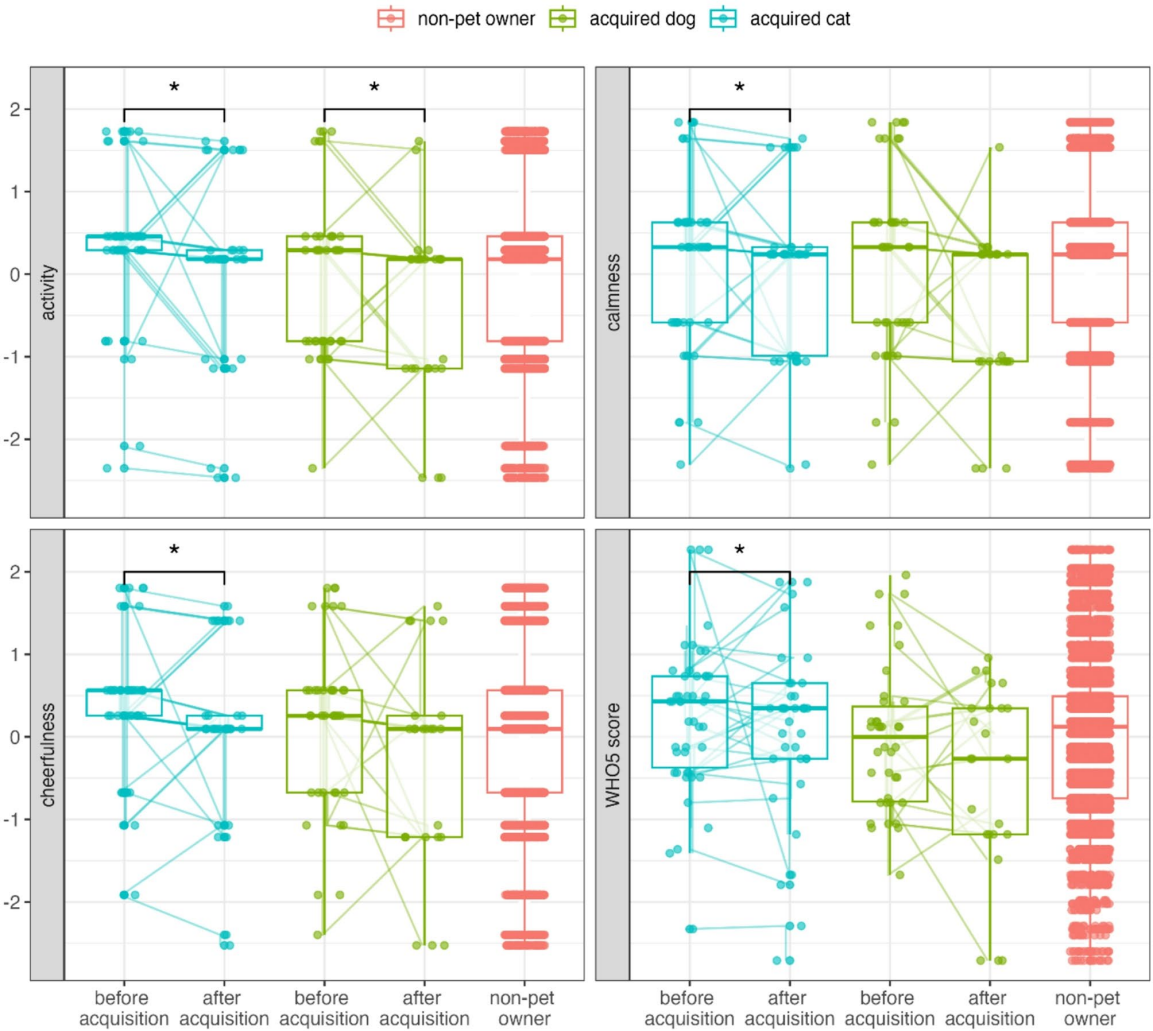
Interaction between acquired species and pet ownership status on activity, calmness, cheerfulness, and WHO-5 score (GLMM set B). Before and after acquisition values for the same participants are connected by lines. Asterisks indicate significant differences. Data points are displayed with transparency and horizontal jitter to minimise overlap.

The interaction between pet species and having a pet during the data collection period revealed that pet species moderated the effect of pet acquisition: participants who acquired a dog were less active (Est. = 0.93, S.E = 0.30, t = 3.13, df = 2963.54, *p* < 0.01), calm (Est. = 0.67, S.E = 0.30, t = 2.23, df = 2961.79, *p* = 0.03), cheerful (Est. = 0.60, S.E = 0.29, t = 2.05, df = 2946.09, *p* = 0.04), and had a lower WHO-5 score (Est. = 0.65, S.E = 0.26, t = 2.46, df = 2888.67, *p* = 0.01) after pet acquisition compared to before pet acquisition, and non-pet owners (Fig. 3).

Participants who acquired a cat were less active post-acquisition than pre-acquisition and were also less active compared to non-pet owners (Est. = 0.83, S.E = 0.28, t = 2.95, df = 2965.33, *p* < 0.01).

The bootstrapped GLMM confirmed that the 95% CIs for interactions between having a pet and pet species on activity did not include zero. For the rest of the effects, the 95% CIs of the estimates included zero, while the 66% CIs excluded zero.

**Fig. 4:**
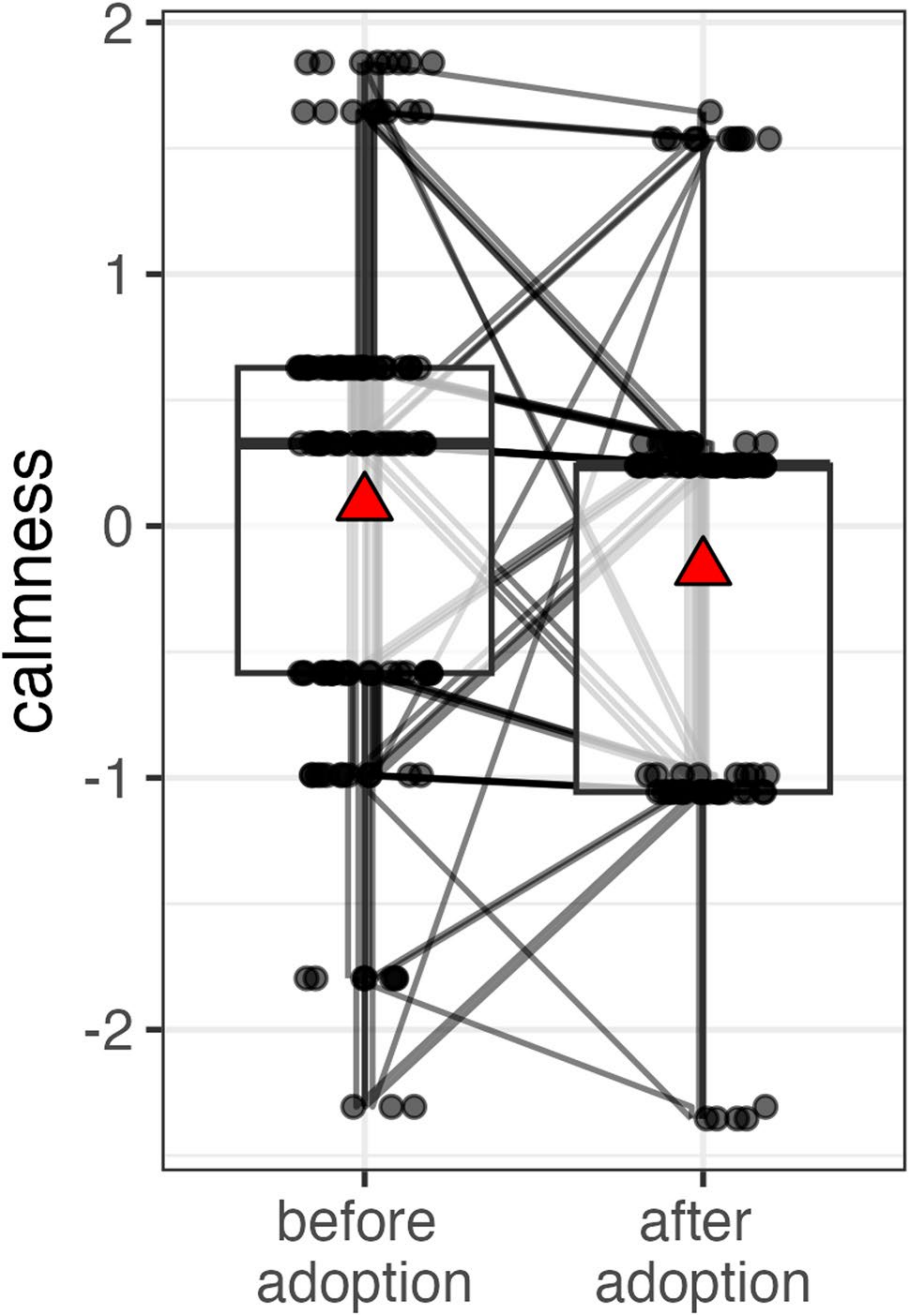
Participants who acquired a pet were less calm post-acquisition compared to pre-acquisition (GLMM set C). Before and after acquisition values for the same participants are connected by lines. Data points are displayed with transparency and horizontal jitter to minimise overlap. Mean values are represented by red triangles.

Participants were less calm after pet acquisition (Est. = −0.74, S.E = 0.35, t = −2.09, df = 131.77, *p* = 0.04, GLMM set C, Fig. 4, Figure S4). While the 95% CIs for this effect included zero in the bootstrapped GLMM, the 66% CIs did not.

**Fig. 5:**
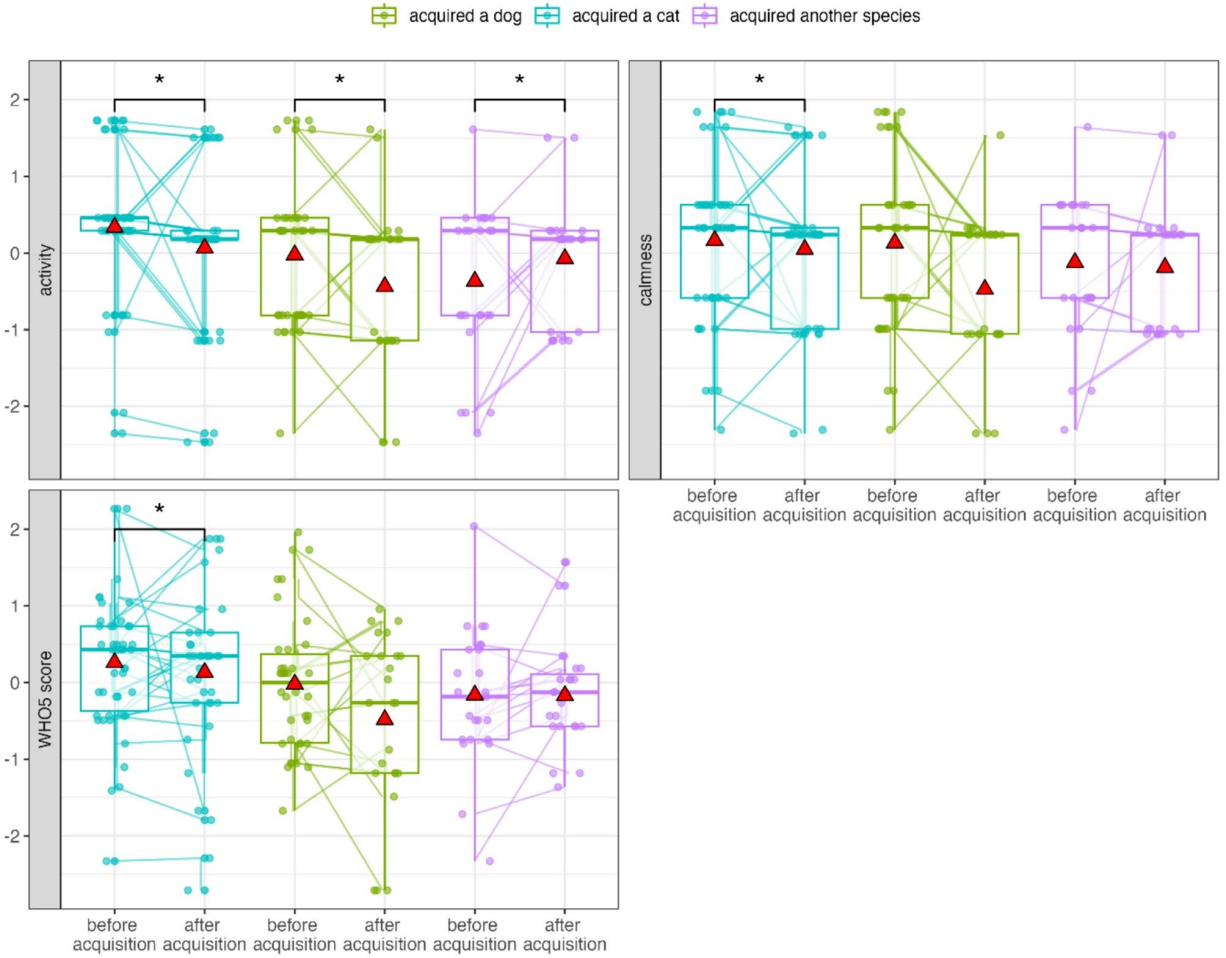
Interaction between the acquired species and pet ownership status on activity, calmness, and WHO-5 score (GLMM set C). Before and after acquisition values for the same participants are connected by lines. Asterisks indicate significant differences. Data points are displayed with transparency and horizontal jitter to minimise overlap. Mean values are represented by red triangles.

Interactions indicated that pet species moderated the effect of acquisition, with well-being outcomes varying by the species of pet acquired (Fig. 5). Participants who acquired dogs or cats became less active after pet acquisition, whereas those acquiring another species became more active (Est. = 0.88, S.E = 0.28, t = 3.11, df = 124.99, *p* < 0.01). Participants who acquired a cat were more active before and after pet acquisition compared to those who acquired a dog. Participants who acquired a dog showed a decrease in calmness after pet acquisition (Est. = 0.69, S.E = 0.30, t = 2.32, df = 125.08, *p* = 0.02), while calmness levels for participants who acquired a cat and those who acquired other animals remained stable. Participants who acquired a dog also experienced a decrease in their WHO-5 scores after acquisition, while scores for participants who acquired a cat and those who acquired other animals remained stable (Est. = 0.65, S.E = 0.28, t = 2.34, df = 123.50, *p* = 0.02). The bootstrapped GLMM showed that the 95% CIs for the interaction between pet ownership and pet species on activity did not include zero, whereas the 95% CIs for the other interactions did include zero, but the 66% CIs excluded zero.

**Fig. 6:**
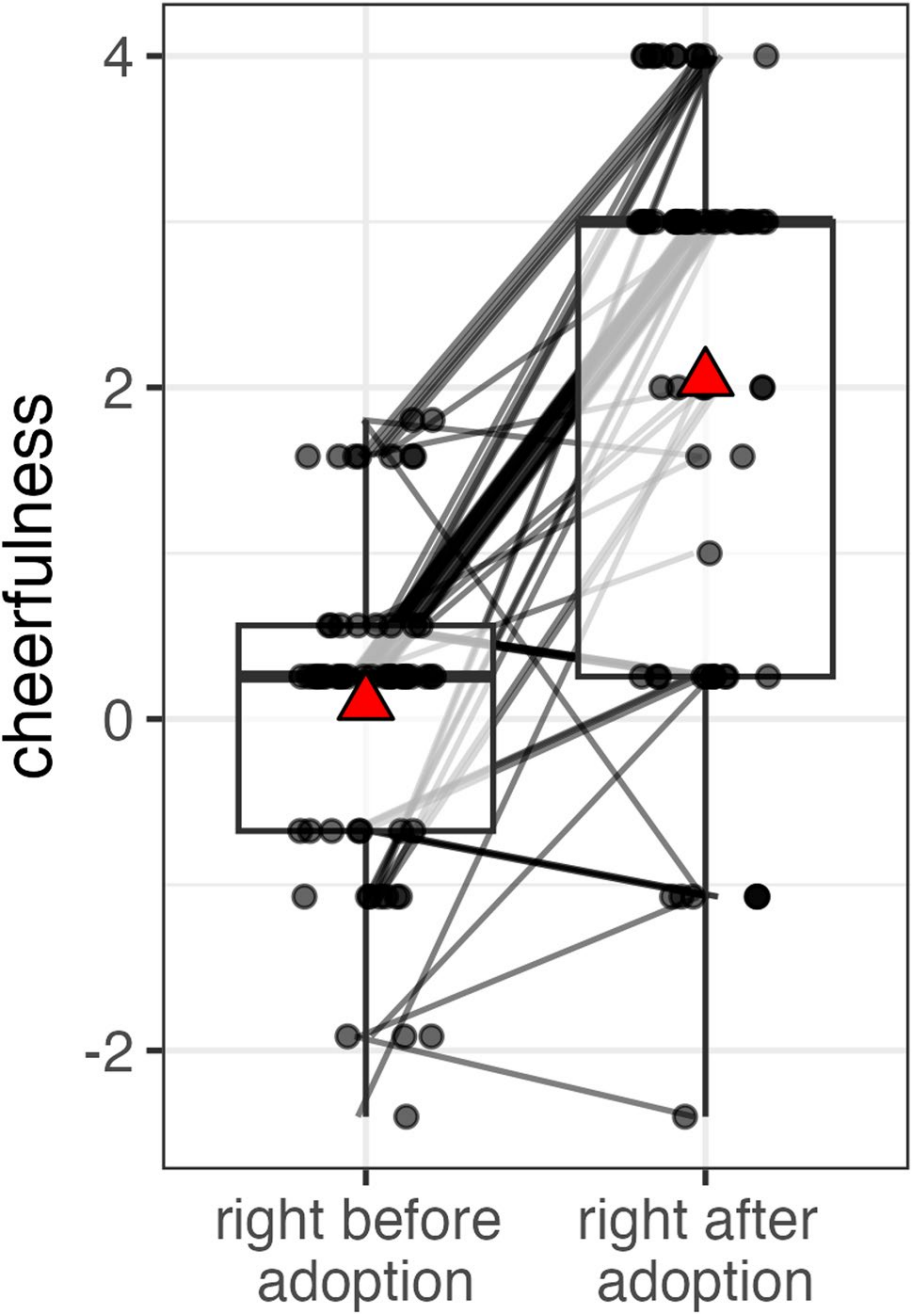
Cheerfulness increased following pet acquisition (main effect of GLMM set D). Before and after acquisition values for the same participants are connected by lines. Data points are displayed with transparency and horizontal jitter to minimise overlap. Mean values are represented by red triangles.

Comparison of participants’ well-being right before and after pet acquisition (1–4 months) showed a significant increase in cheerfulness (Est. = 2.09, S.E = 0.35, t = 5.95, df = 114.00, *p* < 0.01, GLMM set D, Fig. 6, Figure S5). The bootstrapped GLMM showed that the 95% CIs of this effect did not include zero.

**Fig. 7:**
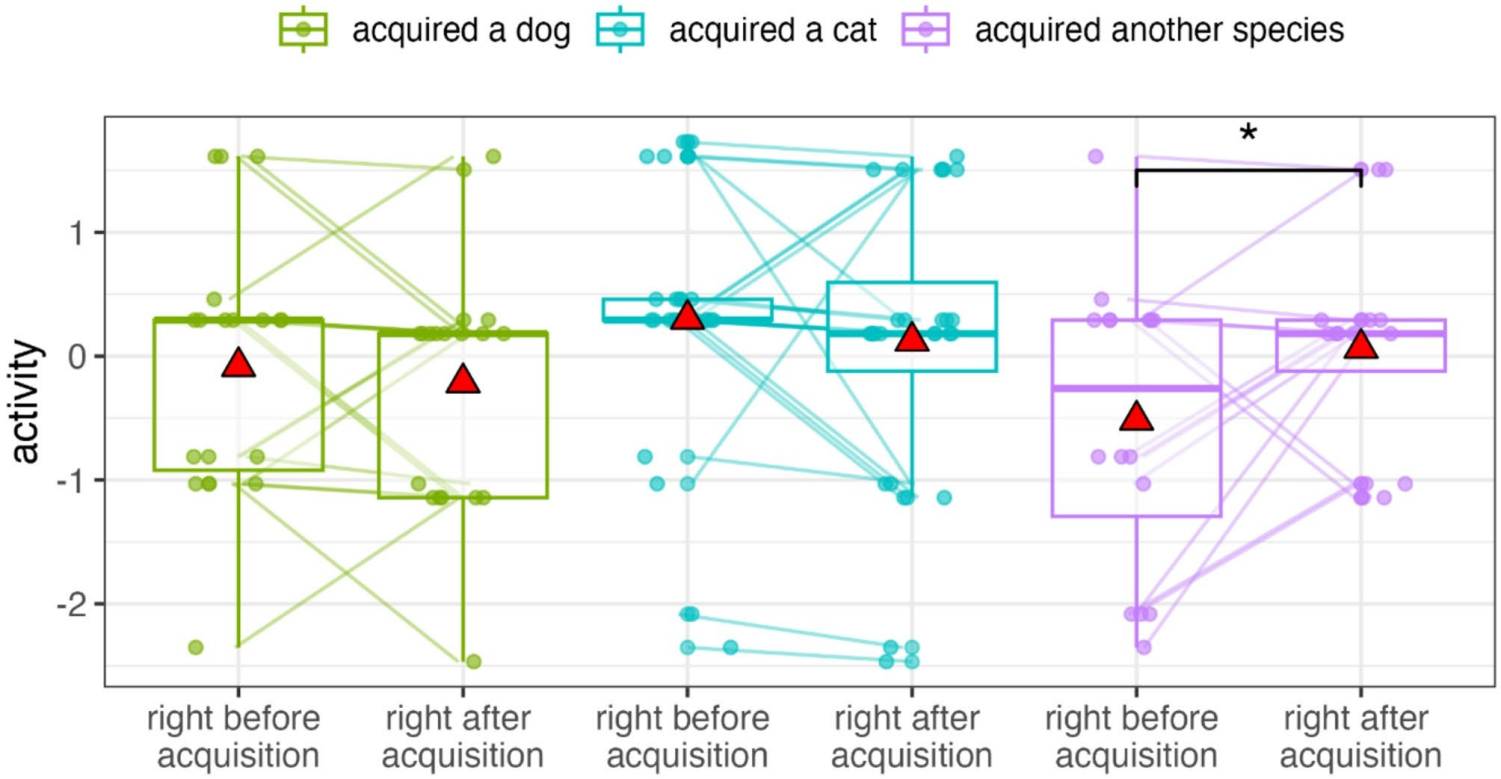
Interaction between time (before or after acquisition) and acquired species on activity (GLMM set D). Before and after acquisition values for the same participants are connected by lines. Asterisks indicate significant differences. Data points are displayed with transparency and horizontal jitter to minimise overlap. Mean values are represented by red triangles.

The interaction between time (before or after the acquisition) and the acquired species showed that species moderated the effect of acquisition: while participants acquiring other pet species became more active after pet acquisition, the activity of individuals acquiring a dog or cat did not change (Est. = 0.71, S.E = 0.35, t = 2.05, df = 60.00, *p* = 0.04, Fig. 7). The bootstrapped GLMM showed that the 95% CIs for these interactions included zero, but the 66% CIs did not.

**Fig. 8:**
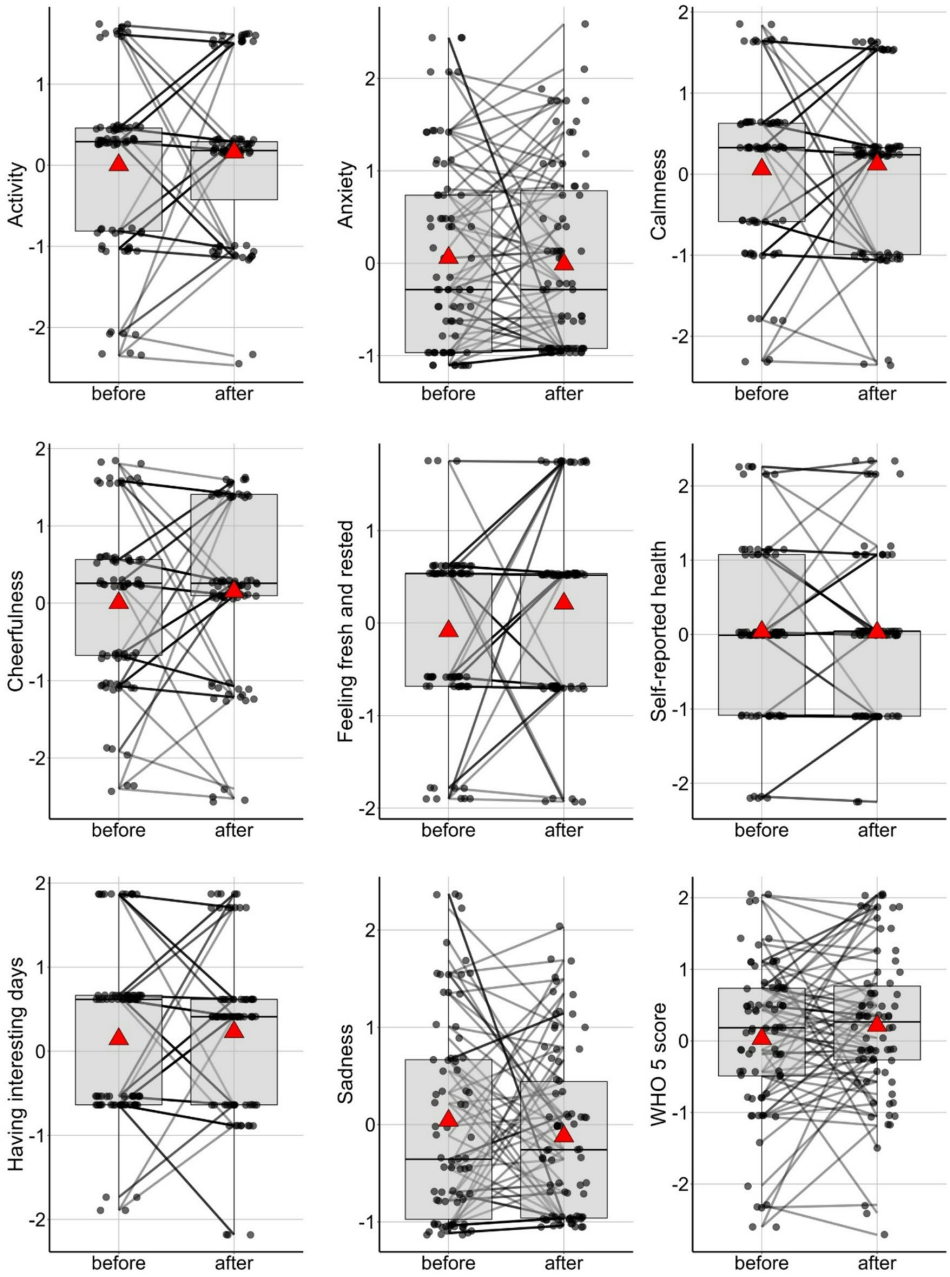
Well-being measures did not change significantly after losing a pet. Mean values are represented by red triangles.

Losing a pet did not affect well-being variables (GLMM set E, Fig. 8, Figure S6).

**Fig. 9:**
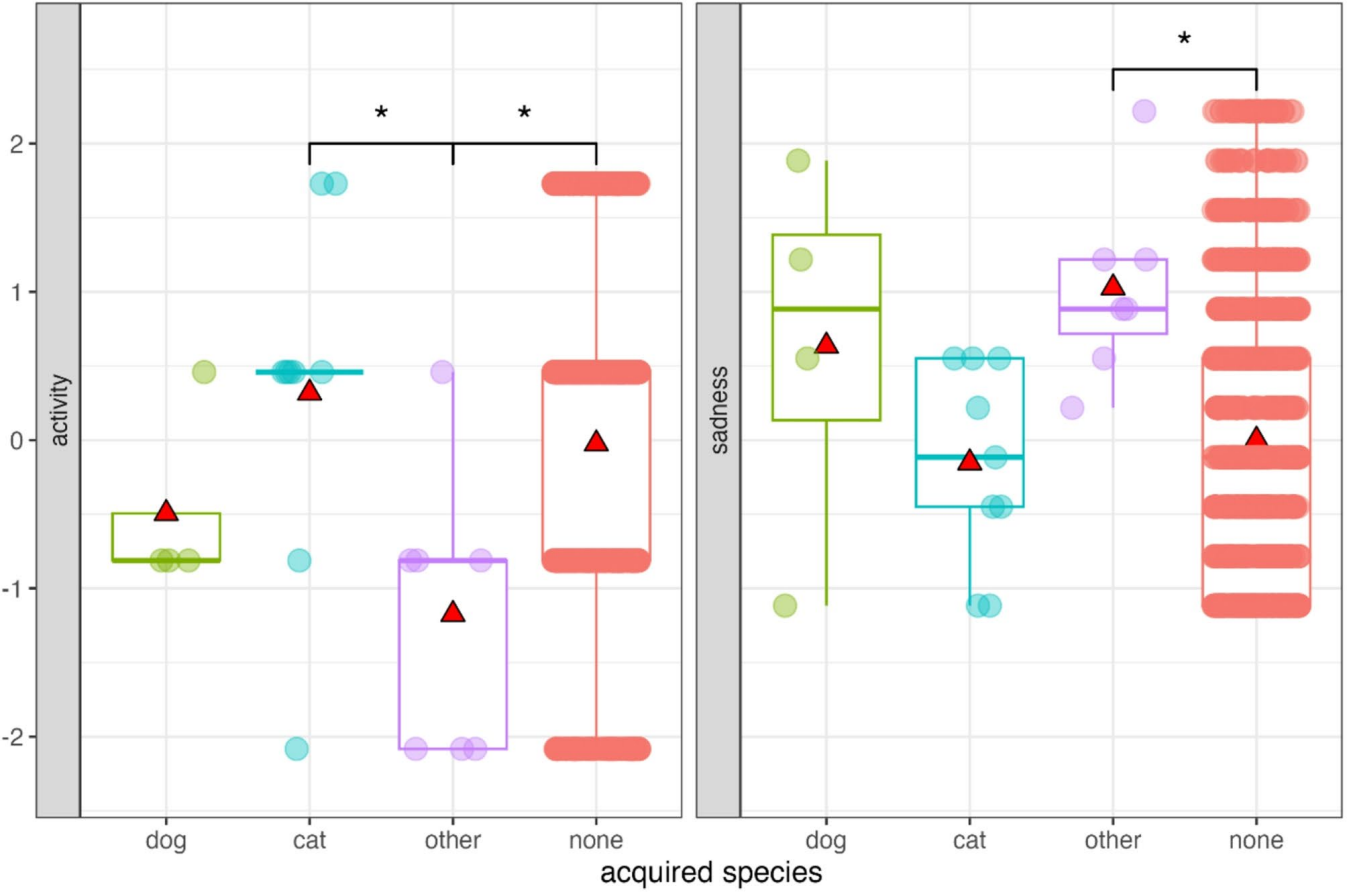
Differences in activity and sadness during the 1st data collection period between participants before acquiring a dog, cat, or other species and non-pet owners in the 2nd period. Asterisks indicate significant differences between individuals before acquiring a pet and non-pet owners. Data points are displayed with transparency and horizontal jitter to minimise overlap. Mean values are represented by red triangles.

Participants, before acquiring other species, were less active than participants acquiring a cat (*p* = 0.025), and they were also less active (*p* = 0.026) and sadder (*p* = 0.044) compared to non-pet owners (Table 3, Table S3, Fig. 9).

**Table 3:** Comparison of mental well-being and physical health among participants acquiring a dog, cat, or other pet, and those with no pets. P-values represent the results of the Kruskal-Wallis tests. Participants who acquired a pet during the 2nd and 3rd periods were analyzed separately.

	Score of 1 st period Acquiring a dog in 2nd period *N* = 4 Acquiring a cat in 2nd period *N* = 9 Acquiring other pet in 2nd period *N* = 7 No pets in 2nd period *N* = 1337			Score of 2nd period Acquiring a dog in 3rd period *N* = 17 Acquiring a cat in 3rd period *N* = 19 Acquiring other pet in 3rd period *N* = 9 No pets in 3rd period *N* = 1337		
	χ^2^	df	p	χ^2^	df	p
Cheerful	5.901	3	0.914	0.937	3	0.404
Calm	10.362	3	0.478	2.066	3	0.957
Active	3.941	3	0.016*	1.916	3	0.559
Feeling fresh and rested	2.484	3	0.174	0.314	3	0.448
Having interesting days	0.520	3	0.161	2.922	3	0.901
Sad	4.976	3	0.037*	2.652	3	0.607
Anxiety	5.151	3	0.268	0.578	3	0.590
WHO-5	8.507	3	0.117	1.836	3	0.817
Health	1.718	3	0.633	6.037	3	0.110

*p < 0.05.

## Discussion

Our data did not support previous findings indicating an increased pet adoption rate during the COVID-19 pandemic^42,50^ with only 2% of participants acquiring and 3% losing a pet during the same period. Several factors may explain this discrepancy. First, Hungary has a high prevalence of pet ownership (38% in a recent representative study^51^ and 52% in the present study), suggesting that those who wanted to keep pets already had one, reducing the need for new acquisitions. Additionally, many acquisitions may have occurred outside the study period, influencing the results. For example, data from Israel^42^ indicates that adoption rates peaked in April 2020 in shelters. If this trend occurred in Hungary as well, these adoptions would not have been reflected in our dataset, as newly adopted animals were already reported in the first period in May 2020. A specific population may also have been underrepresented in the sample, leading to skewed findings. Furthermore, differences in how the pandemic was managed—such as less stringent or shorter lockdowns—could have reduced the perceived need for pets as companions, compared to other countries where stricter restrictions may have heightened adoption motivations^52^. Other factors, such as economic conditions, local cultural attitudes toward pet ownership, and the availability of adoption programs, may have also played a role in the adoption trends observed in different regions.

Our findings also challenge the notion that pet owners fare better on several well-being measures^11,41,53,54^. While we observed an initial increase in cheerfulness following pet acquisition, this positive effect was short-lived, lasting no more than 1–4 months. One possible reason for this could be that prospective pet owners often have strong expectations about the positive impact of living with an animal, such as a happier, more active, less lonely, and less stressed life^55^. In the post-acquisition period, these initial expectations, combined with the novelty effect of the pet, may mask emerging challenges, and owners may not immediately recognise if the animal fails to meet their expectations. However, as the novelty fades, unmet expectations and associated difficulties may negatively impact the owner’s well-being. Over the long term, we found that pet acquisition, particularly dog acquisition, was linked to negative impacts on well-being, including reductions in calmness, cheerfulness, activity levels, and overall life satisfaction. This finding suggests that the unrealistic expectations^56^ and a relatively high burden of dog ownership^29,57^—particularly when the dog has behavioural problems or special needs^13–15,58^—can often have negative consequences on the well-being of humans living in the same household. In addition, we found no evidence to support the assumption that people with lower well-being are more likely to choose dogs to improve their mood, as we found no significant difference in well-being between those intending to acquire a dog and those without pets. However, it is important to note that our participants were not necessarily the primary caretakers of the pets, and therefore, it is possible that, for example, the pet was obtained by a family member against the participant’s will. The results might have been different if we had gathered data exclusively from the primary caretakers of the animals.

Acquiring a cat also negatively impacted activity levels, suggesting that new cat owners spent more time at home^59^. Surprisingly, cat owners remained more active than dog owners, possibly because newly acquired dogs are more challenging to leave at home compared to cats.

While some studies reported poorer sleep quality among pet owners^60–62^ others have shown improved sleep^41^. We found no difference in sleep quality between participants who acquired a pet and those who had no pets. However, we lacked information on whether the participants actually co-slept with their pets, which could have influenced the results.

The impact of pet acquisition on well-being appears to vary by location. While no difference in well-being was observed between participants acquiring a pet and non-owners in the capital city, adopters in county seats reported higher levels of anxiety and sadness. This could be because new owners in the capital have better access to resources for managing the challenges of pet acquisition or due to differences in how the pandemic was experienced in the capital versus county seats.

Contrary to expectations^43–45,63–66^ losing a pet did not affect well-being measures in this study. This lack of effect could be due to the nature of the sample, which represented an average involvement with pets and/or was not necessarily primarily responsible for their care. For example, 36% of participants who lost a dog reported that the pet was not important to them. The varied reasons behind pet loss and the already stressful circumstances of the pandemic may have also contributed. Additionally, it is possible that those who lost a pet might have experienced grief and other negative emotions, such as guilt or anger, but these were not reflected in the surveyed well-being variables. Among the 75 participants who lost a pet, 17 (22.6%) had only owned the pet for a short period, exclusively during the second data collection period, which likely prevented the formation of a strong bond and influenced how they processed the loss^67^. Furthermore, an alternative explanation for the lack of effect on well-being measures after the loss of a pet could be that the Hungarian population has a different attitude toward pets compared to residents of other countries. However, we reject this explanation. According to a recent representative survey in Hungary, 66% of dog- and 63% of cat owners view their pets as family members, 16% vs. 20% consider them “furry kids,” and 12% regard them as more important than anyone else. Additionally, 58% of dogs and 93% of cats have indoor access^51^. These patterns are consistent with findings in other studies. For example, in Victoria, Australia, 72% of dog owners reported that their dog had access to indoor spaces^68^ while in central Italy, 40%^71^ of dog owners indicated the same.

The key strength of this study is its three-wave sampling design, which allowed us to examine whether the absence of a general positive effect on well-being (“pet-effect”^53^) in representative, unbiased samples^2,6,7,9,70,71^ might be explained by the initially lower well-being of prospective pet adopters. If individuals with lower well-being are more likely to acquire pets, their well-being might improve post-acquisition but still not differ from the general population. Our results provided only partial support for this hypothesis. We found that participants planning to acquire pets other than dogs or cats were less active and sadder compared to non-pet owners and also less active than those intending to acquire a cat. This suggests that individuals with a less active lifestyle may prefer low-maintenance, indoor pets such as small rodents, fish, or reptiles, which require fewer lifestyle adjustments. Therefore, while well-being—particularly activity levels—may influence pet acquisition, more detailed data are needed to fully understand these dynamics.

### Limitations

Limitations include the relatively short duration of longitudinal data collection, the specific population (CAWI panel members, representing frequent internet-users), and the specific timing of data collection during a global crisis, which may have influenced participants’ well-being and behaviour. Moreover, sample size differences between participants who acquired a pet and non-pet owners could have distorted the magnitude of the observed differences. We addressed this concern with bootstrapping, which allowed us to derive more reliable estimates of variance, and random effects helped control for individual-level variability. Another limitation is the lack of information on whether the participants were the primary caregivers of the pet or merely lived with it. To mitigate this, we reported that 12–15% of participants whose households acquired or lost a pet lived alone, suggesting they were likely the primary caregivers. Additionally, the majority (87%) of participants reported that the pet was at least “somewhat” important to them, supporting the assumption that most participants had a meaningful relationship with the pet. A further limitation is the lack of specificity regarding the species of pets in the “other” category, which restricted the interpretation of species-specific effects. Future research should aim to examine the combined impact of well-defined pet ownership and external stressors on mental well-being, particularly in non-pandemic times on a larger sample size, to better isolate the specific effects of pet acquisition on long-term well-being.

## Conclusion

Our findings—that acquiring a pet into the household during a global crisis has only a short-term positive effect on well-being, followed by a negative effect in the case of dog acquisition, while losing a pet has no significant impact—challenge the common belief that pet ownership improves well-being. This suggests that results based on representative samples of individuals living with pets but not necessarily the primary caretakers may differ markedly from those obtained from convenience samples, which often include highly devoted pet owners with particularly strong emotional bonds to their pets. This highlights the importance of recognizing potential biases in convenience samples, as their findings may not be generalizable to the broader population. Furthermore, the findings underscore the context-dependent nature of the human-animal bond. The stress and uncertainty of a global crisis may alter the typical emotional and psychological benefits of pet ownership, suggesting that the timing and context of acquisition could be key moderators of its effects on well-being. Future research should explore how broader social and psychological factors shape the impact of pet acquisition over time.

## Electronic supplementary material

Below is the link to the electronic supplementary material.

Supplementary Material 1

## Data Availability

The datasets used and/or analysed during the current study available from the corresponding author on reasonable request.
