# Supplementary material for: Short-term effects of pet acquisition and loss on well-being in an unbiased sample during the COVID-19 pandemic
Source: Sci Rep. 2025 Jul 1;15:20267. doi: 10.1038/s41598-025-06987-7 (PMC12215059; doi:10.1038/s41598-025-06987-7)
Supplement: Supplementary file 1 — Supplementary Material 1 [file 41598_2025_6987_MOESM1_ESM.pdf]

Supplementary material

Short-term effects of pet acquisition and loss on well-being in an unbiased sample during the COVID-19 pandemic

Authors
Judit Mokos[1,†], Eniko Kubinyi[1,2,3,*,†], Dorottya Ujfalussy[1,2], Ivaylo B. Iotchev[1,2], Borbála Paksi[4], Zsolt Demetrovics[5,6,7], Róbert Urbán[5], Ádám Miklósi[2]

[1] MTA-ELTE Lendület "Momentum" Companion Animal Research Group, Budapest, Hungary
[2] Department of Ethology, ELTE Eötvös Loránd University, Budapest, Hungary
[3] ELTE-NAP Dog Brain Research Group, Budapest, Hungary
[4] Institute of Education, ELTE Eötvös Loránd University, Budapest, Hungary
[5] Institute of Psychology, ELTE Eötvös Loránd University, Budapest, Hungary
[6] Centre of Excellence in Responsible Gaming, University of Gibraltar, Gibraltar, Gibraltar
[7] College of Education, Psychology and Social Work, Flinders University, Adelaide, Australia

*Corresponding author
Eniko Kubinyi - eniko.kubinyi@ttk.elte.hu
[†]These authors contributed equally to this work.

Contents

Pandemic-related regulations during data collection 2
Table S1. 2
Figure S1. 3
The change in mental well-being and health over time 4
Table S2 4
Q1. Does acquiring a pet affect mental well-being and health? Figures about the interactions. ... 5
GLMM series A 5
Figure S2. 5
GLMM series B 10
Figure S3. 11
GLMM series C 18
Figure S4. 18
GLMM series D 25
Figure S5. 25
Q2. Does losing a pet affect well-being? 31
GLMM series E 31
Figure S6. 31
Q3. Is there a difference between the well-being of participants who were about to acquire a pet and those who have no pets? 37
Table S3 37
Detailed results of the bootstrapped GLMM series 39
The effect of demographic variables 59

Pandemic-related regulations during data collection

Table S1. Regulations and potential stressors during the three periods of data collection.

**data collection periods**	**1st period**	**2nd period**	**3rd period**
**before the 1st period**	**27 March 2020 – 6 April 2020**	**14 May 2020 – 26 May 2020**	**22 September 2020 – 6 October 2020**
January: Establishment of the Operational Task Force for the Control of the Coronavirus Epidemic. February: shortage of hand sanitizer, face mask, non-perishable food.	8 March: 1st case in Hungary<br>11 March: declaration of a national emergency; universities switched to online teaching; ban of entry from certain countries; reintroduction of border control; mandatory quarantine for returnees from abroad; ban of events for more than 100 people.<br>16 March: the closure of nurseries, kindergartens and schools;	27 April: compulsory mask-wearing in public transport and shops; curfew.<br>4 May: relaxing restrictions, reopening of restaurants, again allowing weddings and funerals and travel abroad.<br>25 May: Reopening of nurseries and kindergartens. Secondary school leaving certificates are written only and can be postponed until September.	June: End of national emergency.<br>August: 2nd period of the epidemic started.<br>September: schools were open, but in case of infection, classes switched to online teaching;
	main message: “Stay at home”		main message: “Wear a mask and carry on”

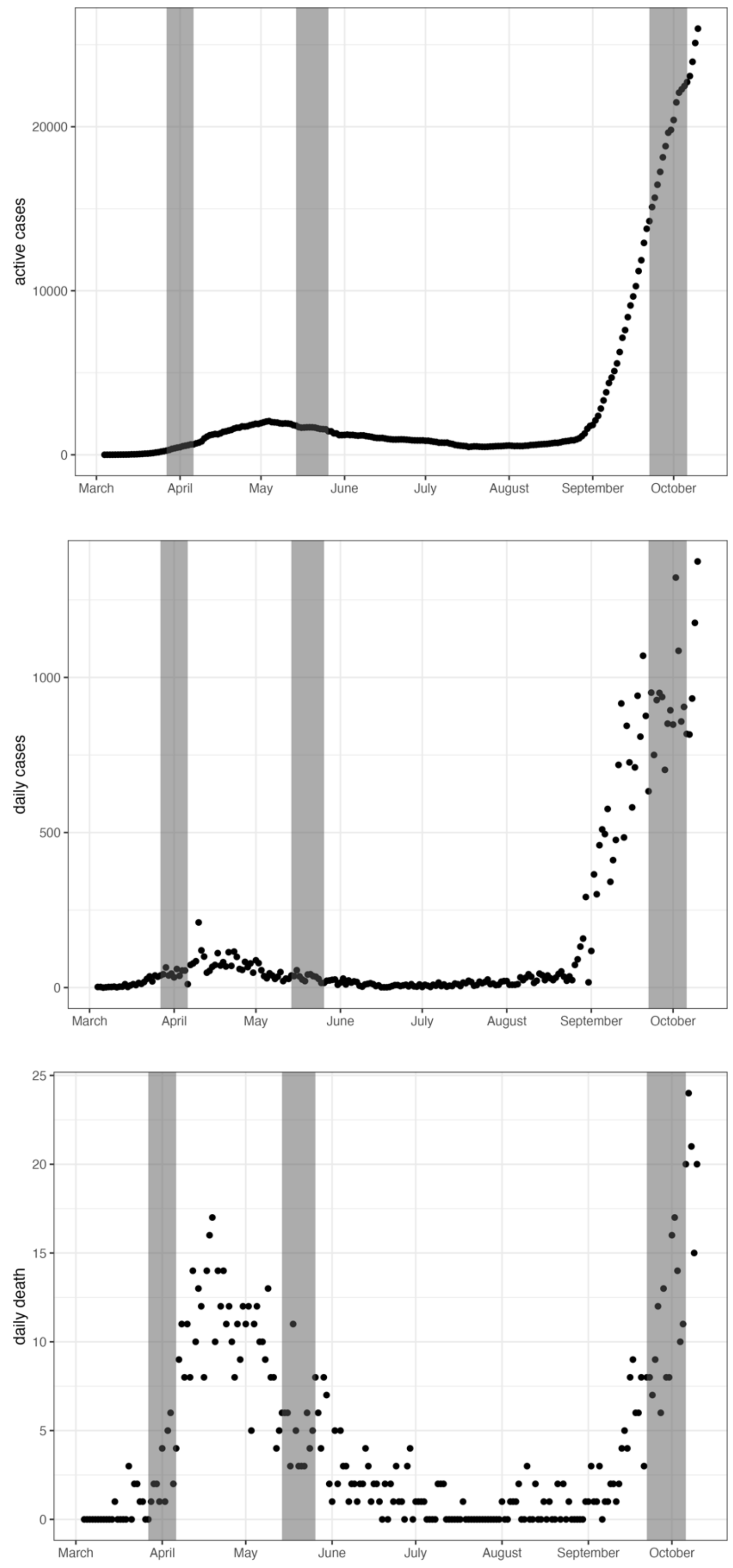

Figure S1. *The daily new infections, the active cases and the daily deaths in Hungary at the time of data collection. The date of data collection is indicated with grey bars. The source of the data is koronavirus.gov.hu, the official government website about the pandemic. The website was shut down on 1st January 2023.*

The change in mental well-being and health over time

The mental well-being of the participants improved over time. A series of Friedmann tests and, in case of significant difference, a pairwise Wilcoxon Rank Sum test with Bonferroni correction as a post hoc test were conducted on participants who participated in all three periods (N=2783) to study how mental well-being changes through time.

Mental well-being improved over time. Physical well-being (Self-reported health) was better in the 2nd period and was slightly worse in the 1st and the 3rd period, which is consistent with the number of infected people in Hungary, which was the highest during the 2nd period.

Table S2. *The mental well-being and health of the participants differ over time. N = 2,783.*

	Friedmann test		mean			Pairwise Wilcoxon Rank Sum test, post hoc					
						1st period – 2nd period		1st period – 3rd period		2nd period – 3rd period	
mental well-being	?^{2}	p value	1st period	2nd period	3rd period	mean difference	p value	mean difference	p value	mean difference	p value
cheerful	674.553	<0.001***	2.545	2.807	2.927	-0.262	<0.001***	-0.382	<0.001***	-0.120	<0.001***
calm	519.676	<0.001***	2.482	2.751	2.815	-0.269	<0.001***	-0.333	<0.001***	-0.063	0.002
active	235.220	<0.001***	2.639	2.780	2.863	-0.141	<0.001***	-0.224	<0.001***	-0.083	<0.001***
feeling fresh and rested	49.444	<0.001***	2.483	2.560	2.577	-0.077	0.003	-0.094	<0.001***	-0.017	1.00
have interesting days	240.307	<0.001***	2.446	2.509	2.683	-0.063	0.012	-0.237	<0.001***	-0.175	<0.001***
sad	132.753	<0.001***	3.347	3.045	2.740	0.302	<0.001***	0.608	<0.001***	0.306	<0.001***
anxiety	287.525	<0.001***	3.479	2.839	2.627	0.640	<0.001***	0.852	<0.001***	0.211	0.029
WHO5	471.692	<0.001***	12.600	13.405	13.867	-0.805	<0.001***	-1.267	<0.001***	-0.462	<0.001***
self-reported health	14.168	<0.001***	2.973	3.010	2.962	-0.037	0.39	0.011	1.00	0.048	0.17

Q1. Does acquiring a pet affect mental well-being and health? Figures about the interactions.

Note that in the Supplementary Material, "adopting" is used as a synonym of "acquisition". In all the following models (A-E), the reference values regarding gender were males, settlement type: village; data collection time: first period. Regarding acquisition status/adopting/losing a pet A-C: yes, D: before, E: after. In the case of pet species: A: -, B: non-other, C-E: dog. Period of acquisition and period when the pet was lost: earlier.

GLMM series A

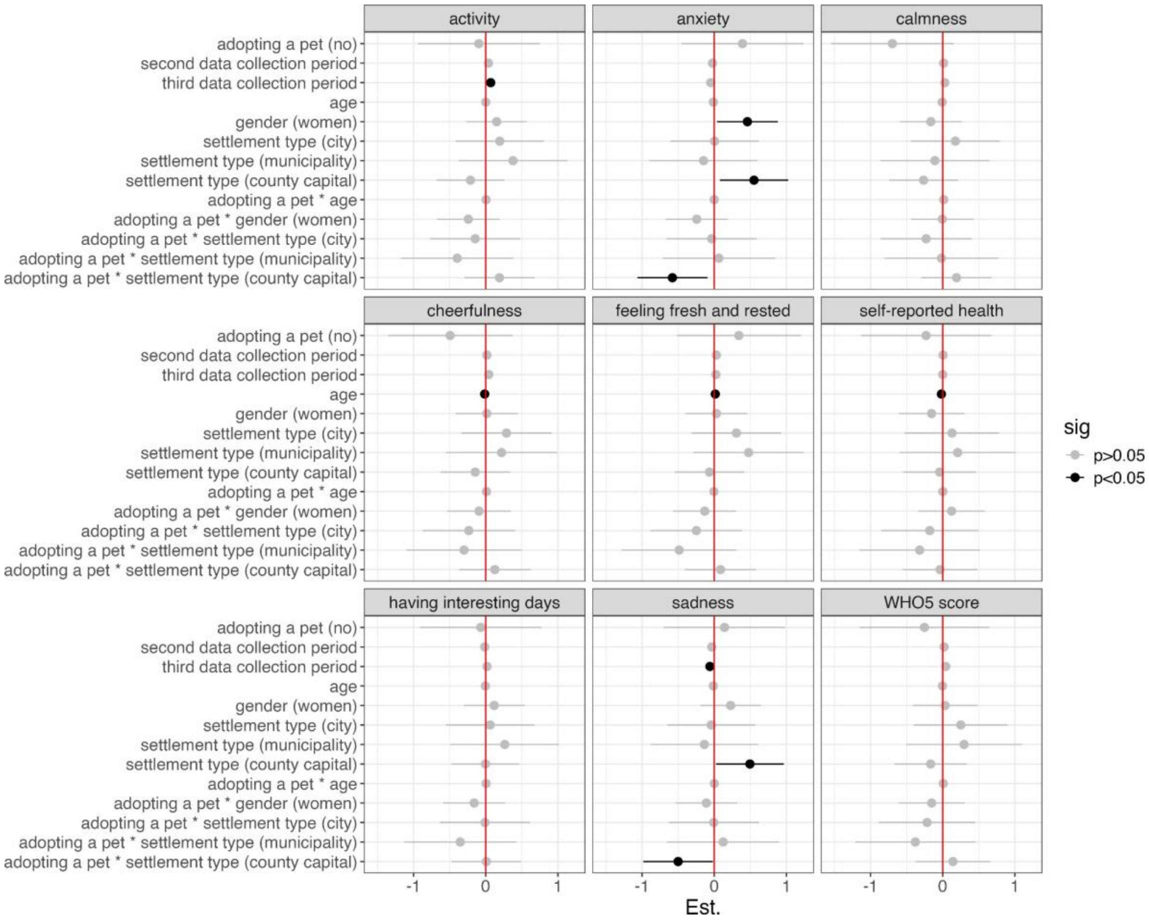

Figure S2. *Forest plot of GLMM series A, representing the standardized beta values and the confidence intervals. Separate models for the well-being measurements were carried out, which are indicated with colours. Significant independent variables are indicated by a full dot.*

Details of the models

Cheerfulness
Observations: 4177
Type: Mixed effects linear regression
MODEL FIT: AIC = 10908.97, BIC = 11010.37 Pseudo-R² (fixed effects) = 0.01 Pseudo-R² (total) = 0.52

Fixed effects:

	Est.	S.E.	t val.	d.f.	p
(Intercept)	0.78	0.43	1.82	1382.58	0.07

data collection period (2nd)	0.02	0.03	0.57	2772.10	0.57
data collection period (3rd)	0.04	0.03	1.58	2776.05	0.12
adopted a pet (no)	-0.49	0.44	-1.12	1379.33	0.26
age	-0.01	0.01	-2.08	1379.05	0.04
gender (woman)	0.01	0.22	0.07	1379.05	0.95
settlement type (city)	0.29	0.32	0.90	1379.05	0.37
settlement type (municipality)	0.22	0.39	0.56	1379.05	0.58
settlement type (county capital)	-0.15	0.25	-0.59	1379.05	0.55
adopted a pet (no):age	0.01	0.01	1.39	1379.36	0.16
adopted a pet (no):gender (woman)	-0.09	0.23	-0.41	1379.26	0.68
adopt:settlement type (city)	-0.24	0.33	-0.72	1379.16	0.47
adopt:settlement type (municipality)	-0.30	0.41	-0.74	1379.50	0.46
adopted a pet (no): settlement type (county capital)	0.13	0.25	0.50	1379.20	0.62

Random effects:

Group	Parameter	Std. Dev.
ID	(Intercept)	0.72
Residual		0.70

Grouping variable:

Group	# groups	ICC
ID	1402	0.51

Calmness

Observations: 4179

Type: Mixed effects linear regression

MODEL FIT: AIC = 11046.63, BIC = 11148.04 Pseudo-R^2 (fixed effects) = 0.02 Pseudo-R^2 (total) = 0.49

Fixed effects:

	Est.	S.E.	t val.	d.f.	p
(Intercept)	0.55	0.42	1.30	1383.26	0.19
data collection period (2nd)	0.01	0.03	0.46	2774.16	0.65
data collection period (3rd)	0.03	0.03	1.06	2778.65	0.29
adopted a pet (no)	-0.70	0.43	-1.61	1379.67	0.11
age	-0.01	0.01	-1.09	1379.38	0.27
gender (woman)	-0.16	0.22	-0.76	1379.38	0.45
settlement type (city)	0.18	0.31	0.56	1379.38	0.58
settlement type (municipality)	-0.11	0.39	-0.28	1379.38	0.78
settlement type (county capital)	-0.27	0.24	-1.09	1379.38	0.27
adopted a pet (no):age	0.01	0.01	1.84	1379.69	0.07
adopted a pet (no):gender (woman)	-0.01	0.22	-0.02	1379.59	0.98
adopt:settlement type (city)	-0.23	0.32	-0.72	1379.48	0.47
adopt:settlement type (municipality)	-0.02	0.40	-0.04	1379.87	0.97
adopted a pet (no) :settlement type (county capital)	0.19	0.25	0.76	1379.53	0.45

Random effects:

Group	Parameter	Std. Dev.
ID	(Intercept)	0.69
Residual		0.72

Grouping variable:

Group	# groups	ICC
ID	1402	0.48

Activity
Observations: 4176
Type: Mixed effects linear regression
MODEL FIT: AIC = 10973.07, BIC = 11074.47 Pseudo-R^2 (fixed effects) = 0.01 Pseudo-R^2 (total) = 0.49

Fixed effects:

	Est.	S.E.	t val.	d.f.	p
(Intercept)	-0.12	0.42	-0.28	1383.96	0.78
data collection period (2nd)	0.04	0.03	1.40	2773.50	0.16
data collection period (3rd)	0.07	0.03	2.53	2777.67	0.01
adopted a pet (no)	-0.10	0.43	-0.22	1380.37	0.82
age	-0.00	0.01	-0.04	1380.07	0.97
gender (woman)	0.15	0.22	0.70	1380.07	0.48
settlement type (city)	0.19	0.31	0.62	1380.07	0.53
settlement type (municipality)	0.38	0.38	0.99	1380.07	0.32
settlement type (county capital)	-0.21	0.24	-0.88	1380.07	0.38
adopted a pet (no):age	0.00	0.01	0.65	1380.40	0.52
adopted a pet (no):gender (woman)	-0.24	0.22	-1.09	1380.31	0.27
adopt:settlement type (city)	-0.15	0.32	-0.46	1380.22	0.65
adopt:settlement type (municipality)	-0.40	0.40	-0.99	1380.58	0.32
adopted a pet (no) :settlement type (county capital)	0.19	0.25	0.77	1380.27	0.44

Random effects:

Group	Parameter	Std. Dev.
ID	(Intercept)	0.69
Residual		0.71

Grouping variable:

Group	# groups	ICC
ID	1402	0.48

Feeling fresh and rested
Observations: 4170
Type: Mixed effects linear regression
MODEL FIT: AIC = 10639.41, BIC = 10740.78 Pseudo-R^2 (fixed effects) = 0.03 Pseudo-R^2 (total) = 0.55

Fixed effects:

	Est.	S.E.	t val.	d.f.	p
(Intercept)	-0.86	0.42	-2.01	1398.37	0.04
data collection period (2nd)	0.03	0.03	1.17	2769.22	0.24
data collection period (3rd)	0.02	0.03	0.92	2773.52	0.36
adopted a pet (no)	0.34	0.44	0.78	1394.67	0.44
age	0.01	0.01	2.05	1390.56	0.04
gender (woman)	0.03	0.22	0.14	1382.98	0.89
settlement type (city)	0.31	0.32	0.97	1384.36	0.33
settlement type (municipality)	0.48	0.39	1.22	1387.06	0.22
settlement type (county capital)	-0.07	0.24	-0.27	1390.91	0.79
adopted a pet (no):age	-0.00	0.01	-0.57	1390.46	0.57
adopted a pet (no):gender (woman)	-0.13	0.22	-0.59	1383.15	0.55

adopt:settlement type (city)	-0.25	0.32	-0.76	1384.40	0.44
adopt:settlement type (municipality)	-0.49	0.41	-1.20	1387.08	0.23
adopted a pet (no) :settlement type (county capital)	0.09	0.25	0.34	1390.58	0.73

Random effects:

Group	Parameter	Std. Dev.
ID	(Intercept)	0.72
Residual		0.67

Grouping variable:

Group	# groups	ICC
ID	1402	0.53

Having interesting days

Observations: 4168

Type: Mixed effects linear regression

MODEL FIT: AIC = 11167.55, BIC = 11268.91 Pseudo-R^2 (fixed effects) = 0.00 Pseudo-R^2 (total) = 0.45

Fixed effects:

	Est.	S.E.	t val.	d.f.	p
(Intercept)	0.08	0.42	0.19	1386.69	0.85
data collection period (2nd)	-0.01	0.03	-0.51	2770.57	0.61
data collection period (3rd)	0.02	0.03	0.61	2775.60	0.54
adopted a pet (no)	-0.07	0.43	-0.16	1382.80	0.87
age	-0.01	0.01	-0.74	1391.38	0.46
gender (woman)	0.12	0.22	0.54	1385.27	0.59
settlement type (city)	0.06	0.31	0.20	1390.40	0.84
settlement type (municipality)	0.26	0.38	0.69	1382.99	0.49
settlement type (county capital)	-0.00	0.24	-0.02	1392.86	0.99
adopted a pet (no):age	0.01	0.01	0.71	1391.24	0.48
adopted a pet (no):gender (woman)	-0.16	0.22	-0.73	1385.40	0.47
adopt:settlement type (city)	-0.01	0.32	-0.03	1390.17	0.97
adopt:settlement type (municipality)	-0.35	0.40	-0.89	1383.36	0.37
adopted a pet (no):settlement type (county capital)	0.01	0.25	0.03	1392.50	0.98

Random effects:

Group	Parameter	Std. Dev.
ID	(Intercept)	0.68
Residual		0.74

Grouping variable:

Group	# groups	ICC
ID	1402	0.45

Sadness

Observations: 4178

Type: Mixed effects linear regression

MODEL FIT: AIC = 10770.23, BIC = 10871.63 Pseudo-R^2 (fixed effects) = 0.04 Pseudo-R^2 (total) = 0.52

Fixed effects:

	Est.	S.E.	t val.	d.f.	p
(Intercept)	0.41	0.42	0.98	1400.02	0.33
data collection period (2nd)	-0.03	0.03	-1.32	2777.27	0.19
data collection period (3rd)	-0.06	0.03	-2.29	2778.83	0.02
adopted a pet (no)	0.14	0.43	0.33	1395.93	0.74
age	-0.01	0.01	-1.85	1391.45	0.06

gender (woman)	0.23	0.21	1.06	1383.19	0.29
settlement type (city)	-0.04	0.31	-0.13	1384.70	0.89
settlement type (municipality)	-0.14	0.38	-0.36	1387.64	0.72
settlement type (county capital)	0.49	0.24	2.07	1391.83	0.04
adopted a pet (no):age	0.00	0.01	0.28	1391.26	0.78
adopted a pet (no):gender (woman)	-0.11	0.22	-0.50	1383.32	0.62
adopt:settlement type (city)	-0.01	0.32	-0.02	1384.75	0.98
adopt:settlement type (municipality)	0.12	0.40	0.31	1387.70	0.76
adopted a pet (no):settlement type (county capital)	-0.50	0.25	-2.03	1391.53	0.04

Random effects:

Group	Parameter	Std. Dev.
ID	(Intercept)	0.69
Residual		0.69

Grouping variable:

Group	# groups	ICC
ID	1402	0.50

Anxiety

Observations: 4176

Type: Mixed effects linear regression

MODEL FIT: AIC = 10581.86, BIC = 10683.25 Pseudo-R^2 (fixed effects) = 0.05 Pseudo-R^2 (total) = 0.55

Fixed effects:

	Est.	S.E.	t val.	d.f.	p
(Intercept)	0.15	0.42	0.36	1401.48	0.72
data collection period (2nd)	-0.02	0.03	-0.94	2777.63	0.35
data collection period (3rd)	-0.05	0.03	-1.90	2778.83	0.06
adopted a pet (no)	0.39	0.43	0.91	1397.77	0.36
age	-0.01	0.01	-1.76	1393.51	0.08
gender (woman)	0.46	0.22	2.13	1385.78	0.03
settlement type (city)	0.00	0.31	0.01	1387.20	0.99
settlement type (municipality)	-0.15	0.38	-0.39	1389.95	0.70
settlement type (county capital)	0.55	0.24	2.29	1393.87	0.02
adopted a pet (no):age	0.00	0.01	0.08	1393.35	0.94
adopted a pet (no):gender (woman)	-0.24	0.22	-1.10	1385.94	0.27
adopt:settlement type (city)	-0.04	0.32	-0.11	1387.28	0.91
adopt:settlement type (municipality)	0.06	0.40	0.16	1390.02	0.87
adopted a pet (no):settlement type (county capital)	-0.58	0.25	-2.34	1393.64	0.02

Random effects:

Group	Parameter	Std. Dev.
ID	(Intercept)	0.70
Residual		0.67

Grouping variable:

Group	# groups	ICC
ID	1402	0.53

WHO-5 score

Observations: 4154

Type: Mixed effects linear regression

MODEL FIT: AIC = 10314.24, BIC = 10415.55 Pseudo-R^2 (fixed effects) = 0.01 Pseudo-R^2 (total) = 0.61

Fixed effects:

	Est.	S.E.	t val.	d.f.	p
(Intercept)	0.11	0.44	0.24	1393.55	0.81
data collection period (2nd)	0.02	0.02	0.83	2754.13	0.41
data collection period (3rd)	0.04	0.02	1.76	2757.31	0.08
adopted a pet (no)	-0.25	0.46	-0.55	1390.69	0.58
age	-0.00	0.01	-0.44	1393.24	0.66
gender (woman)	0.03	0.23	0.15	1383.01	0.88
settlement type (city)	0.25	0.33	0.75	1387.62	0.45
settlement type (municipality)	0.30	0.41	0.72	1384.82	0.47
settlement type (county capital)	-0.17	0.26	-0.66	1394.67	0.51
adopted a pet (no):age	0.01	0.01	0.95	1392.92	0.34
adopted a pet (no):gender (woman)	-0.15	0.23	-0.66	1383.14	0.51
adopt:settlement type (city)	-0.22	0.34	-0.64	1387.43	0.52
adopt:settlement type (municipality)	-0.38	0.42	-0.90	1384.75	0.37
adopted a pet (no):settlement type (county capital)	0.14	0.26	0.54	1394.11	0.59

Random effects:

Group	Parameter	Std. Dev.
ID	(Intercept)	0.77
Residual		0.62

Grouping variable:

Group	# groups	ICC
ID	1402	0.61

Self-reported health

Observations: 4196

Type: Mixed effects linear regression

MODEL FIT: AIC = 9549.21, BIC = 9650.68 Pseudo-R^2 (fixed effects) = 0.06 Pseudo-R^2 (total) = 0.72

Fixed effects:

	Est.	S.E.	t val.	d.f.	p
(Intercept)	1.25	0.45	2.80	1390.68	0.01
data collection period (2nd)	0.00	0.02	0.22	2792.78	0.82
data collection period (3rd)	0.00	0.02	0.02	2793.47	0.99
adopted a pet (no)	-0.23	0.46	-0.50	1388.83	0.62
age	-0.02	0.01	-2.49	1389.24	0.01
gender (woman)	-0.15	0.23	-0.67	1390.08	0.50
settlement type (city)	0.13	0.33	0.38	1398.03	0.70
settlement type (municipality)	0.21	0.41	0.50	1388.80	0.62
settlement type (county capital)	-0.04	0.26	-0.16	1388.79	0.87
adopted a pet (no):age	0.00	0.01	0.19	1389.24	0.85
adopted a pet (no):gender (woman)	0.12	0.24	0.52	1390.06	0.60
adopt:settlement type (city)	-0.18	0.34	-0.53	1397.66	0.60
adopt:settlement type (municipality)	-0.32	0.43	-0.75	1388.93	0.45
adopted a pet (no):settlement type (county capital)	-0.04	0.26	-0.14	1388.81	0.89

Random effects:

Group	Parameter	Std. Dev.
ID	(Intercept)	0.80
Residual		0.53

Grouping variable:

Group	# groups	ICC
ID	1402	0.70

GLMM series B

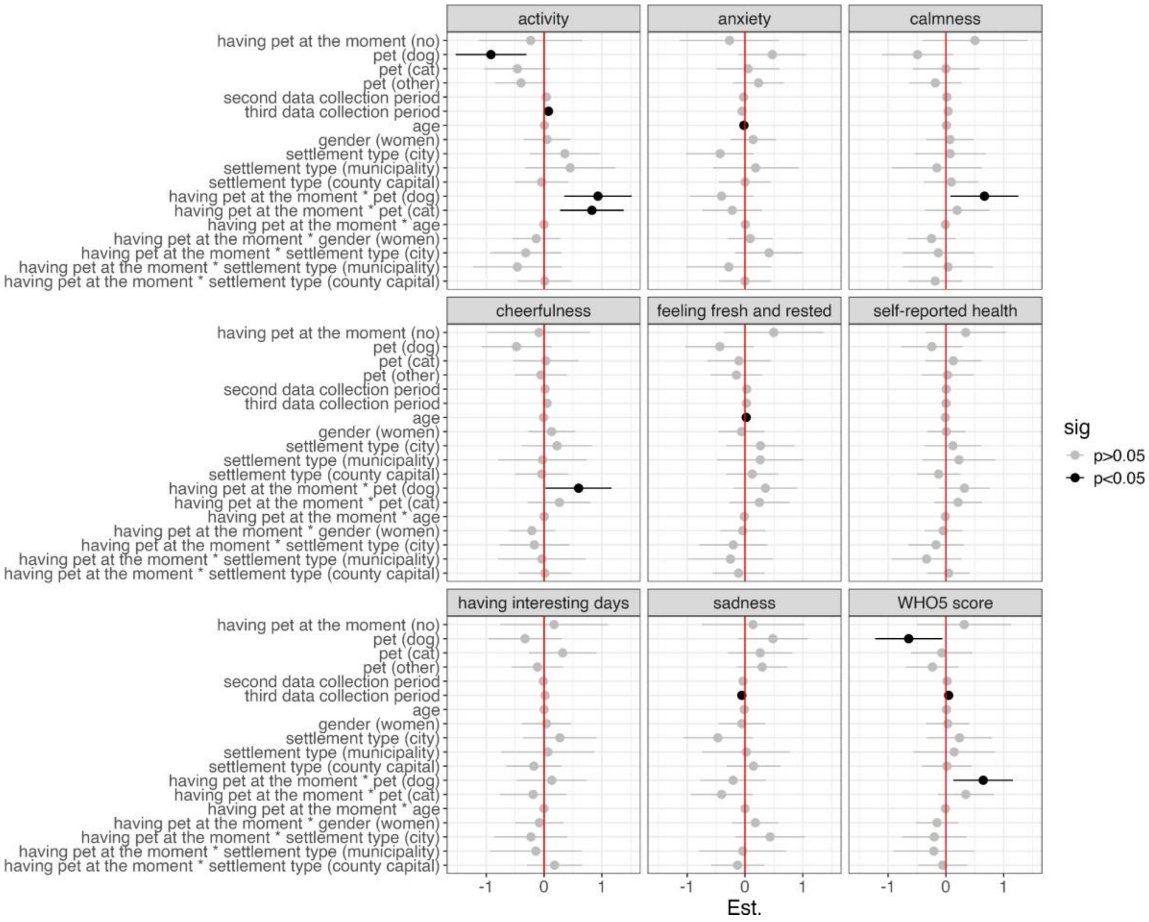

Figure S3. *Forest plot of GLMM series B, representing the standardized beta values and the confidence intervals. Separate models for the well-being measurements were carried out. The level of significance is indicated with different colours.*

Cheerfulness

Observations: 4177

Type: Mixed effects linear regression

MODEL FIT: AIC = 10920.10, BIC = 11053.18 Pseudo-R² (fixed effects) = 0.01 Pseudo-R² (total) = 0.52

Fixed effects:

	Est.	S.E.	t val.	d.f.	p
(Intercept)	0.40	0.46	0.88	3817.87	0.38
data collection period (2nd)	0.02	0.03	0.66	2765.16	0.51
data collection period (3rd)	0.05	0.03	1.89	2772.08	0.06
pet (dog)	-0.48	0.31	-1.54	4136.29	0.12
pet (cat)	0.03	0.29	0.11	4156.20	0.92
pet (other)	-0.06	0.23	-0.24	2038.69	0.81
have a pet at the moment (no)	-0.09	0.45	-0.19	3605.18	0.85
age	-0.01	0.01	-1.18	3879.66	0.24
gender (woman)	0.13	0.21	0.63	3988.08	0.53
settlement type (city)	0.22	0.31	0.73	3955.27	0.47
settlement type (municipality)	-0.02	0.39	-0.06	3995.99	0.95
settlement type (county capital)	-0.04	0.23	-0.17	3961.92	0.86
pet (dog):have a pet at the moment (no)	0.60	0.29	2.05	2946.09	0.04
pet (cat):have a pet at the moment (no)	0.26	0.28	0.96	2947.12	0.34
have a pet at the moment (no) : age	0.00	0.01	0.43	3688.35	0.66
have a pet at the moment (no) :gender (woman)	-0.21	0.21	-1.02	3842.39	0.31
have a pet at the moment (no) :settlement type	-0.17	0.31	-0.55	3799.59	0.58

(city)					
have a pet at the moment (no) settlement type (municipality)	-0.04	0.39	-0.10	3748.91	0.92
have a pet at the moment (no) :settlement type (county capital)	0.01	0.23	0.06	3756.67	0.95

Random effects:

Group	Parameter	Std. Dev.
ID	(Intercept)	0.72
Residual		0.70

Grouping variable:

Group	# groups	ICC
ID	1402	0.51

Calmness

Observations: 4179 Type: Mixed effects linear regression

MODEL FIT: AIC = 11053.17, BIC = 11186.27 Pseudo-R^2 (fixed effects) = 0.02 Pseudo-R^2 (total) = 0.49

Fixed effects:

	Est.	S.E.	t val.	d.f.	p
(Intercept)	-0.60	0.47	-1.28	3851.47	0.20
data collection period (2nd)	0.02	0.03	0.56	2765.91	0.57
data collection period (3rd)	0.04	0.03	1.39	2773.61	0.16
pet (dog)	-0.49	0.31	-1.56	4153.37	0.12
pet (cat)	-0.00	0.29	-0.00	4149.25	1.00
pet (other)	-0.18	0.23	-0.80	2091.69	0.42
have a pet at the moment (no)	0.50	0.46	1.09	3662.46	0.28
age	0.01	0.01	1.60	3917.66	0.11
gender (woman)	0.07	0.21	0.33	4025.05	0.74
settlement type (city)	0.07	0.31	0.23	3994.09	0.82
settlement type (municipality)	-0.16	0.40	-0.39	4024.44	0.69
settlement type (county capital)	0.09	0.24	0.40	3995.93	0.69
pet (dog):have a pet at the moment (no)	0.67	0.30	2.23	2961.79	0.03
pet (cat):have a pet at the moment (no)	0.19	0.28	0.69	2963.51	0.49
have a pet at the moment (no) age	-0.01	0.01	-0.94	3749.74	0.35
have a pet at the moment (no) gender (woman)	-0.25	0.21	-1.17	3902.38	0.24
have a pet at the moment (no) :settlement type (city)	-0.13	0.31	-0.41	3861.14	0.68
have a pet at the moment (no) :settlement type (municipality)	0.04	0.40	0.09	3811.00	0.93
have a pet at the moment (no) :settlement type (county capital)	-0.19	0.24	-0.78	3818.83	0.44

Random effects:

Group	Parameter	Std. Dev.
ID	(Intercept)	0.70
Residual		0.72

Grouping variable:

Group	# groups	ICC
ID	1402	0.48

Activity

Observations: 4176
Type: Mixed effects linear regression
MODEL FIT: AIC = 10975.84, BIC = 11108.92 Pseudo-R^2 (fixed effects) = 0.01 Pseudo-R^2 (total) = 0.49

Fixed effects:

	Est.	S.E.	t val.	d.f.	p
(Intercept)	0.04	0.46	0.09	3852.08	0.93
data collection period (2nd)	0.04	0.03	1.44	2767.77	0.15
data collection period (3rd)	0.08	0.03	2.75	2775.20	0.01
pet (dog)	-0.92	0.31	-2.94	4151.40	0.00
pet (cat)	-0.46	0.29	-1.59	4145.04	0.11
pet (other)	-0.40	0.23	-1.75	2100.03	0.08
have a pet at the moment (no)	-0.23	0.46	-0.51	3665.75	0.61
age	0.01	0.01	0.82	3918.41	0.41
gender (woman)	0.05	0.21	0.24	4025.26	0.81
settlement type (city)	0.36	0.31	1.15	3994.60	0.25
settlement type (municipality)	0.45	0.40	1.14	4023.97	0.25
settlement type (county capital)	-0.04	0.24	-0.19	3996.05	0.85
pet (dog):have a pet at the moment (no)	0.93	0.30	3.13	2963.54	0.00
pet (cat):have a pet at the moment (no)	0.83	0.28	2.95	2965.33	0.00
have a pet at the moment (no) age	-0.00	0.01	-0.26	3753.06	0.80
have a pet at the moment (no) :gender (woman)	-0.14	0.21	-0.65	3904.93	0.52
have a pet at the moment (no) :settlement type (city)	-0.32	0.31	-1.02	3864.00	0.31
have a pet at the moment (no) :settlement type (municipality)	-0.46	0.39	-1.18	3814.14	0.24
have a pet at the moment (no) :settlement type (county capital)	0.01	0.24	0.05	3821.93	0.96

Random effects:

Group	Parameter	Std. Dev.
ID	(Intercept)	0.69
Residual		0.71

Grouping variable:

Group	# groups	ICC
ID	1402	0.48

Feeling fresh and rested
Observations: 4170
Type: Mixed effects linear regression
MODEL FIT: AIC = 10653.59, BIC = 10786.64 Pseudo-R^2 (fixed effects) = 0.03 Pseudo-R^2 (total) = 0.55

Fixed effects:

	Est.	S.E.	t val.	d.f.	p
(Intercept)	-1.01	0.45	-2.26	3796.43	0.02
data collection period (2nd)	0.03	0.03	1.17	2762.93	0.24
data collection period (3rd)	0.02	0.03	0.94	2769.75	0.35
pet (dog)	-0.43	0.30	-1.43	4117.18	0.15
pet (cat)	-0.10	0.28	-0.37	4150.95	0.71
pet (other)	-0.15	0.23	-0.65	2001.27	0.51
have a pet at the moment (no)	0.50	0.44	1.13	3572.60	0.26
age	0.02	0.01	3.19	3849.75	0.00
gender (woman)	-0.06	0.20	-0.30	3949.67	0.77
settlement type (city)	0.27	0.30	0.89	3917.25	0.37
settlement type (municipality)	0.26	0.38	0.69	3970.65	0.49
settlement type (county capital)	0.13	0.23	0.54	3933.97	0.59
pet (dog):have a pet at the moment (no)	0.35	0.28	1.26	2941.64	0.21
pet (cat):have a pet at the moment (no)	0.25	0.26	0.94	2932.65	0.35
have a pet at the moment (no) :age	-0.01	0.01	-1.69	3646.27	0.09
have a pet at the moment (no) :gender (woman)	-0.04	0.20	-0.19	3785.94	0.85
have a pet at the moment (no) :settlement type (city)	-0.20	0.30	-0.68	3746.90	0.50
have a pet at the moment (no) :settlement type (municipality)	-0.25	0.38	-0.67	3703.22	0.51

have a pet at the moment (no) settlement type (county capital)	-0.11	0.23	-0.48	3716.65	0.63

Random effects:

Group	Parameter	Std. Dev.
ID	(Intercept)	0.72
Residual		0.67

Grouping variable:

Group	# groups	ICC
ID	1402	0.53

Having interesting days

Observations: 4168 Dependent Variable: value Type: Mixed effects linear regression

MODEL FIT: AIC = 11177.64, BIC = 11310.68 Pseudo-R^2 (fixed effects) = 0.00 Pseudo-R^2 (total) = 0.45

Fixed effects:

	Est.	S.E.	t val.	d.f.	p
(Intercept)	-0.14	0.48	-0.29	3881.98	0.77
data collection period (2nd)	-0.01	0.03	-0.51	2765.47	0.61
data collection period (3rd)	0.02	0.03	0.63	2773.84	0.53
pet (dog)	-0.33	0.32	-1.03	4148.91	0.30
pet (cat)	0.32	0.30	1.07	4120.51	0.28
pet (other)	-0.12	0.23	-0.50	2159.55	0.61
have a pet at the moment (no)	0.17	0.48	0.37	3720.00	0.71
age	0.00	0.01	0.18	3957.07	0.85
gender (woman)	0.04	0.22	0.20	4053.89	0.85
settlement type (city)	0.27	0.32	0.85	4031.56	0.40
settlement type (municipality)	0.06	0.41	0.16	4046.75	0.87
settlement type (county capital)	-0.18	0.24	-0.72	4031.67	0.47
pet (dog):have a pet at the moment (no)	0.13	0.31	0.43	2975.44	0.67
pet (cat):have a pet at the moment (no)	-0.19	0.29	-0.65	2980.10	0.52
have a pet at the moment (no) :age	-0.00	0.01	-0.26	3818.44	0.79
have a pet at the moment (no) pet:gender (woman)	-0.08	0.22	-0.38	3957.45	0.70
have a pet at the moment (no) :settlement type (city)	-0.23	0.32	-0.70	3927.33	0.48
have a pet at the moment (no) :settlement type (municipality)	-0.14	0.41	-0.35	3873.32	0.73
have a pet at the moment (no) :settlement type (county capital)	0.18	0.24	0.74	3892.04	0.46

Random effects:

Group	Parameter	Std. Dev.
ID	(Intercept)	0.67
Residual		0.74

Grouping variable:

Group	# groups	ICC
ID	1402	0.45

Sadness

Observations: 4178 Dependent Variable: value Type: Mixed effects linear regression

MODEL FIT: AIC = 10784.54, BIC = 10917.63 Pseudo-R^2 (fixed effects) = 0.04 Pseudo-R^2 (total) = 0.52

Fixed effects:

	Est.	S.E.	t val.	d.f.	p

(Intercept)	0.39	0.46	0.85	3842.57	0.40
data collection period (2nd)	-0.03	0.03	-1.26	2770.98	0.21
data collection period (3rd)	-0.06	0.03	-2.07	2775.24	0.04
pet (dog)	0.48	0.31	1.57	4148.00	0.12
pet (cat)	0.26	0.28	0.93	4154.31	0.35
pet (other)	0.30	0.22	1.32	2064.04	0.19
have a pet at the moment (no)	0.14	0.45	0.31	3647.89	0.76
age	-0.01	0.01	-1.75	3901.40	0.08
gender (woman)	-0.05	0.20	-0.26	4001.33	0.79
settlement type (city)	-0.47	0.31	-1.53	3970.91	0.13
settlement type (municipality)	0.02	0.39	0.06	4010.82	0.95
settlement type (county capital)	0.15	0.23	0.62	3981.60	0.53
pet (dog):have a pet at the moment (no)	-0.21	0.29	-0.71	2967.01	0.48
pet (cat):have a pet at the moment (no)	-0.40	0.27	-1.48	2957.79	0.14
have a pet at the moment (no) :age	0.00	0.01	0.21	3726.61	0.83
have a pet at the moment (no) pet:gender (woman)	0.18	0.20	0.88	3866.23	0.38
have a pet at the moment (no) :settlement type (city)	0.43	0.31	1.42	3828.42	0.16
have a pet at the moment (no) :settlement type (municipality)	-0.04	0.39	-0.10	3784.78	0.92
have a pet at the moment (no) settlement type (county	-0.13	0.23	-0.54	3798.38	0.59

Random effects:

Group	Parameter	Std. Dev.
ID	(Intercept)	0.69
Residual		0.69

Grouping variable:

Group	# groups	ICC
ID	1402	0.50

Anxiety

Observations: 4176

Type: Mixed effects linear regression

MODEL FIT: AIC = 10599.26, BIC = 10732.34 Pseudo-R^2 (fixed effects) = 0.05 Pseudo-R^2 (total) = 0.55

Fixed effects:

	Est.	S.E.	t val.	d.f.	p
(Intercept)	0.77	0.44	1.73	3809.03	0.08
data collection period (2nd)	-0.02	0.03	-0.94	2769.17	0.35
data collection period (3rd)	-0.05	0.03	-1.92	2772.85	0.06
pet (dog)	0.47	0.30	1.57	4127.41	0.12
pet (cat)	0.05	0.28	0.19	4156.98	0.85
pet (other)	0.23	0.22	1.04	2012.10	0.30
have a pet at the moment (no)	-0.27	0.44	-0.61	3589.96	0.54
age	-0.02	0.01	-2.76	3863.32	0.01
gender (woman)	0.14	0.20	0.71	3963.40	0.48
settlement type (city)	-0.43	0.30	-1.43	3931.32	0.15
settlement type (municipality)	0.19	0.38	0.49	3982.39	0.62
settlement type (county capital)	0.00	0.23	0.01	3947.03	0.99
pet (dog):have a pet at the moment (no)	-0.40	0.28	-1.44	2950.33	0.15
pet (cat):have a pet at the moment (no)	-0.22	0.26	-0.84	2941.29	0.40
have a pet at the moment (no) :age	0.01	0.01	1.08	3664.61	0.28
have a pet at the moment (no) pet:gender (woman)	0.09	0.20	0.45	3804.47	0.65
have a pet at the moment (no) :settlement type (city)	0.41	0.30	1.39	3765.55	0.16
have a pet at the moment (no) :settlement type (municipality)	-0.28	0.37	-0.74	3721.81	0.46
have a pet at the moment (no) :settlement type (county capital)	0.00	0.23	0.00	3735.26	1.00

Random effects:

Group	Parameter	Std. Dev.
ID	(Intercept)	0.71
Residual		0.67

Grouping variable:

Group	# groups	ICC
ID	1402	0.53

WHO-5 score
Observations: 4154
Type: Mixed effects linear regression
MODEL FIT: AIC = 10323.21, BIC = 10456.18 Pseudo-R^2 (fixed effects) = 0.01 Pseudo-R^2 (total) = 0.61

Fixed effects:

	Est.	S.E.	t val.	d.f.	p
(Intercept)	-0.43	0.42	-1.01	3711.83	0.31
data collection period (2nd)	0.02	0.02	0.89	2747.21	0.38
data collection period (3rd)	0.05	0.02	1.97	2752.21	0.05
pet (dog)	-0.64	0.30	-2.17	3990.65	0.03
pet (cat)	-0.07	0.27	-0.26	4094.95	0.79
pet (other)	-0.23	0.23	-1.00	1871.61	0.32
have a pet at the moment (no)	0.32	0.42	0.76	3413.47	0.45
age	0.01	0.01	1.65	3756.17	0.10
gender (woman)	0.03	0.19	0.17	3842.66	0.87
settlement type (city)	0.24	0.29	0.82	3815.46	0.41
settlement type (municipality)	0.14	0.36	0.39	3898.23	0.69
settlement type (county capital)	0.02	0.22	0.08	3851.49	0.94
pet (dog):have a pet at the moment (no)	0.65	0.26	2.46	2888.67	0.01
pet (cat):have a pet at the moment (no)	0.34	0.25	1.40	2882.03	0.16
have a pet at the moment (no) : age	-0.01	0.01	-1.15	3482.87	0.25
have a pet at the moment (no) :gender (woman)	-0.15	0.19	-0.80	3607.81	0.42
have a pet at the moment (no) :settlement type (city)	-0.20	0.28	-0.71	3578.77	0.48
have a pet at the moment (no) :settlement type (municipality)	-0.21	0.36	-0.59	3530.13	0.56
have a pet at the moment (no): settlement type (county capital)	-0.05	0.22	-0.25	3554.82	0.80

Random effects:

Group	Parameter	Std. Dev.
ID	(Intercept)	0.77
Residual		0.62

Grouping variable:

Group	# groups	ICC
ID	1402	0.61

Self-reported health
Observations: 4196
Type: Mixed effects linear regression
MODEL FIT: AIC = 9562.56, BIC = 9695.74 Pseudo-R^2 (fixed effects) = 0.06 Pseudo-R^2 (total) = 0.71

Fixed effects:

	Est.	S.E.	t val.	d.f.	p
(Intercept)	0.69	0.37	1.89	3717.83	0.06
data collection period (2nd)	0.00	0.02	0.25	2786.22	0.81
data collection period (3rd)	0.00	0.02	0.16	2788.33	0.87

pet (dog)	-0.24	0.27	-0.90	3704.24	0.37
pet (cat)	0.13	0.25	0.52	3924.68	0.60
pet (other)	0.03	0.23	0.12	1751.40	0.90
have a pet at the moment (no)	0.34	0.36	0.96	3282.52	0.34
age	-0.01	0.01	-1.60	3738.24	0.11
gender (woman)	0.01	0.17	0.04	3808.56	0.97
settlement type (city)	0.12	0.25	0.48	3788.78	0.63
settlement type (municipality)	0.23	0.32	0.71	3917.43	0.48
settlement type (county capital)	-0.13	0.19	-0.66	3837.73	0.51
pet (dog):have a pet at the moment (no)	0.32	0.22	1.44	2886.64	0.15
pet (cat):have a pet at the moment (no)	0.21	0.21	1.00	2888.68	0.32
have a pet at the moment (no) :age	-0.01	0.01	-1.52	3332.42	0.13
have a pet at the moment (no) pet:gender (woman)	-0.05	0.16	-0.28	3455.80	0.78
have a pet at the moment (no) :settlement type (city)	-0.17	0.25	-0.69	3434.33	0.49
have a pet at the moment (no) :settlement type (municipality)	-0.33	0.31	-1.09	3375.09	0.28
have a pet at the moment (no) :settlement type (county capital)	0.05	0.19	0.26	3380.56	0.80

Random effects:

Group	Parameter	Std. Dev.
ID	(Intercept)	0.80
Residual		0.53

Grouping variable:

Group	# groups	ICC
ID	1402	0.70

GLMM series C

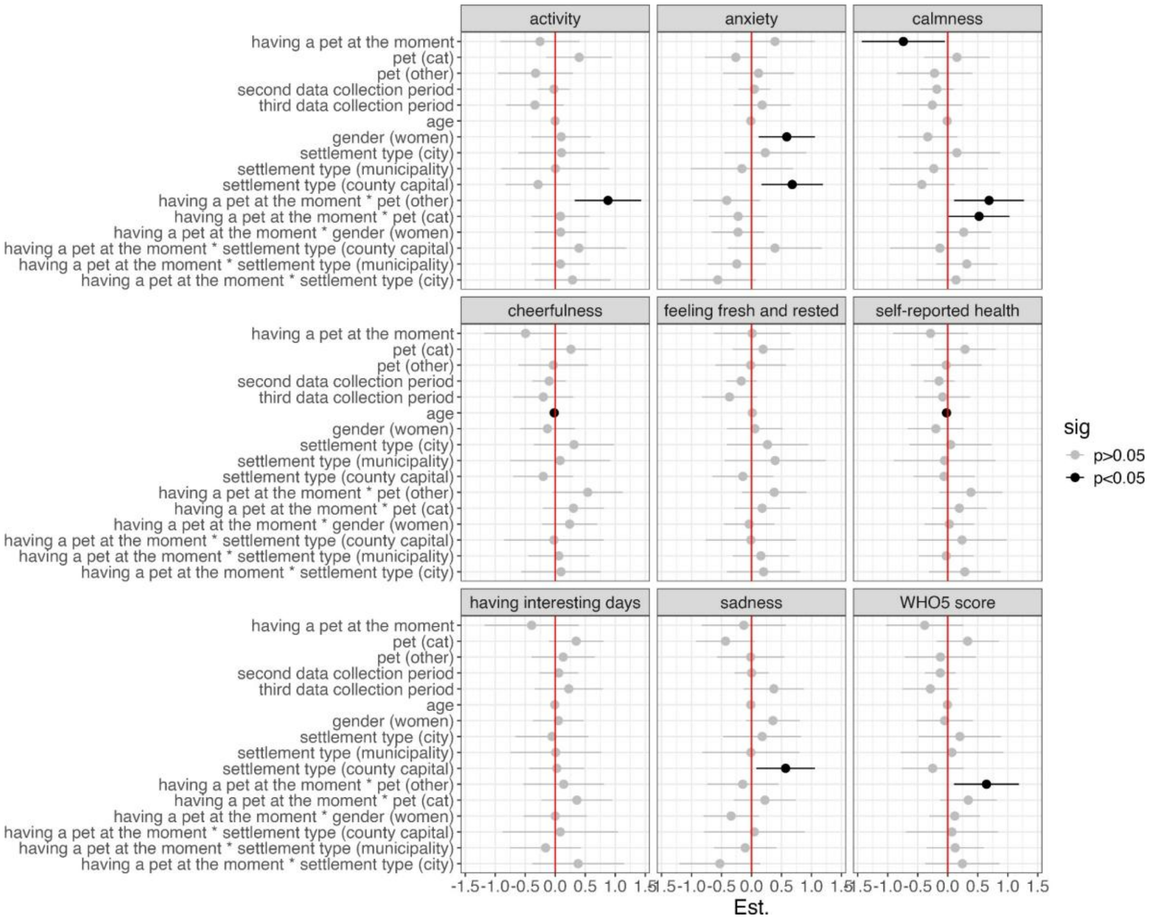

Figure S4. *Forest plot of GLMM series C, representing the standardized beta values and the confidence intervals. Separate models for the well-being measurements were carried out. The level of significance is indicated with different colours.*

Details of the models

Cheerfulness

Observations: 195 Dependent Variable: value Type: Mixed effects linear regression

MODEL FIT: AIC = 551.84, BIC = 614.02 Pseudo-R^2 (fixed effects) = 0.11 Pseudo-R^2 (total) = 0.51

Fixed effects:

	Est.	S.E.	t val.	d.f.	p
(Intercept)	1.05	0.43	2.45	68.72	0.02
data collection period (2nd)	-0.10	0.14	-0.71	125.35	0.48
data collection period (3rd)	-0.20	0.26	-0.77	139.33	0.44
pet (cat)	0.26	0.26	1.02	77.59	0.31
pet (other)	-0.03	0.30	-0.11	81.57	0.91
have pet at the moment (yes)	-0.50	0.35	-1.40	133.53	0.16
age	-0.02	0.01	-2.53	54.68	0.01
gender (woman)	-0.13	0.23	-0.55	86.72	0.58
settlement type (city)	0.31	0.34	0.92	81.66	0.36
settlement type (municipality)	0.08	0.43	0.19	80.73	0.85
settlement type (county capital)	-0.20	0.26	-0.78	81.53	0.44
pet (cat):have pet at the moment (yes)	0.30	0.26	1.17	124.32	0.25
pet (other):have pet at the moment (yes)	0.54	0.30	1.81	125.46	0.07
have pet at the moment (yes)	0.24	0.23	1.02	126.08	0.31
pet:gender (woman)					
have pet at the moment (yes):settlement type (city)	0.10	0.34	0.28	126.33	0.78

have pet at the moment (yes):settlement type (municipality)	-0.02	0.42	-0.05	126.53	0.96
have pet at the moment (yes):settlement type (county capital)	0.06	0.26	0.22	126.46	0.82

Random effects:

Group	Parameter	Std. Dev.
ID	(Intercept)	0.64
Residual		0.72

Grouping variable:

Group	# groups	ICC
ID	65	0.45

Calmness

Observations: 195

Type: Mixed effects linear regression

MODEL FIT: AIC = 561.70, BIC = 623.89 Pseudo-R² (fixed effects) = 0.10 Pseudo-R² (total) = 0.56

Fixed effects:

	Est.	S.E.	t val.	d.f.	p
(Intercept)	1.01	0.47	2.15	67.15	0.03
data collection period (2nd)	-0.18	0.14	-1.26	125.16	0.21
data collection period (3rd)	-0.26	0.26	-1.00	136.62	0.32
pet (cat)	0.15	0.28	0.55	74.47	0.58
pet (other)	-0.22	0.32	-0.69	77.81	0.49
have pet at the moment (yes)	-0.74	0.35	-2.09	131.77	0.04
age	-0.01	0.01	-1.31	55.58	0.19
gender (woman)	-0.33	0.25	-1.32	82.15	0.19
settlement type (city)	0.15	0.37	0.41	77.88	0.68
settlement type (municipality)	-0.23	0.46	-0.51	77.10	0.61
settlement type (county capital)	-0.43	0.28	-1.55	77.77	0.12
pet (cat):have pet at the moment (yes)	0.52	0.26	2.02	124.16	0.05
pet (other):have pet at the moment (yes)	0.69	0.30	2.32	125.08	0.02
have pet at the moment (yes)	0.27	0.23	1.14	125.58	0.26
pet:gender (woman)					
have pet at the moment (yes) :settlement type (city)	0.14	0.34	0.41	125.78	0.68
have pet at the moment (yes):settlement type (municipality)	-0.13	0.42	-0.31	125.94	0.76
have pet at the moment (yes):settlement type (county capital)	0.32	0.26	1.23	125.89	0.22

Random effects:

Group	Parameter	Std. Dev.
ID	(Intercept)	0.74
Residual		0.71

Grouping variable:

Group	# groups	ICC
ID	65	0.52

Activity

Observations: 195

Type: Mixed effects linear regression
MODEL FIT: AIC = 549.81, BIC = 612.00 Pseudo-R^2 (fixed effects) = 0.11 Pseudo-R^2 (total) = 0.60

Fixed effects:

	Est.	S.E.	t val.	d.f.	p
(Intercept)	0.07	0.47	0.15	66.46	0.88
data collection period (2nd)	-0.02	0.14	-0.17	125.13	0.86
data collection period (3rd)	-0.34	0.25	-1.38	135.35	0.17
pet (cat)	0.40	0.28	1.43	73.02	0.16
pet (other)	-0.33	0.32	-1.03	76.03	0.31
have pet at the moment (yes)	-0.25	0.34	-0.76	130.99	0.45
age	-0.00	0.01	-0.29	56.11	0.78
gender (woman)	0.10	0.25	0.39	79.94	0.69
settlement type (city)	0.10	0.37	0.28	76.08	0.78
settlement type (municipality)	0.00	0.46	0.00	75.39	1.00
settlement type (county capital)	-0.29	0.28	-1.03	75.99	0.31
pet (cat):have pet at the moment (yes)	0.09	0.25	0.35	124.18	0.73
pet (other):have pet at the moment (yes)	0.88	0.28	3.11	124.99	0.00
have pet at the moment (yes)<br>pet:gender (woman)	0.09	0.22	0.40	125.44	0.69
have pet at the moment (yes):settlement type (city)	0.29	0.32	0.90	125.61	0.37
have pet at the moment (yes):settlement type (municipality)	0.40	0.40	0.98	125.75	0.33
have pet at the moment (yes):settlement type (county capital)	0.09	0.25	0.35	125.70	0.72

Random effects:

Group	Parameter	Std. Dev.
ID	(Intercept)	0.75
Residual		0.68

Grouping variable:

Group	# groups	ICC
ID	65	0.55

Feeling fresh and rested
Observations: 194
Type: Mixed effects linear regression
MODEL FIT: AIC = 528.87, BIC = 590.96 Pseudo-R^2 (fixed effects) = 0.10 Pseudo-R^2 (total) = 0.59

Fixed effects:

	Est.	S.E.	t val.	d.f.	p
(Intercept)	-0.77	0.44	-1.75	67.24	0.08
data collection period (2nd)	-0.17	0.13	-1.30	124.65	0.20
data collection period (3rd)	-0.37	0.23	-1.56	135.23	0.12
pet (cat)	0.19	0.26	0.74	74.01	0.46
pet (other)	-0.01	0.30	-0.05	77.16	0.96
have pet at the moment (yes)	0.01	0.33	0.04	131.91	0.97
age	0.01	0.01	1.89	56.50	0.06
gender (woman)	0.06	0.24	0.24	81.23	0.81
settlement type (city)	0.27	0.34	0.77	77.23	0.44
settlement type (municipality)	0.39	0.43	0.92	76.47	0.36
settlement type (county capital)	-0.14	0.26	-0.55	77.10	0.58
pet (cat):have pet at the moment (yes)	0.18	0.24	0.75	124.44	0.45
pet (other):have pet at the moment (yes)	0.38	0.27	1.38	125.27	0.17
have pet at the moment (yes):gender (woman)	-0.04	0.21	-0.18	125.26	0.86
have pet at the moment (yes):settlement type (city)	0.20	0.31	0.65	126.28	0.52
have pet at the moment (yes):settlement type (municipality)	-0.01	0.39	-0.03	125.82	0.98
have pet at the moment (yes) : settlement type (county capital)	0.16	0.24	0.65	126.72	0.52

Random effects:

Group	Parameter	Std. Dev.
ID	(Intercept)	0.70
Residual		0.65

Grouping variable:

Group	# groups	ICC
ID	65	0.54

Having interesting days
Observations: 194
Type: Mixed effects linear regression
MODEL FIT: AIC = 565.94, BIC = 628.03 Pseudo-R² (fixed effects) = 0.08 Pseudo-R² (total) = 0.26

Fixed effects:

	Est.	S.E.	t val.	d.f.	p
(Intercept)	0.12	0.38	0.31	81.39	0.76
data collection period (2nd)	0.06	0.17	0.35	130.22	0.73
data collection period (3rd)	0.23	0.29	0.78	153.67	0.44
pet (cat)	0.35	0.23	1.51	98.11	0.14
pet (other)	0.13	0.27	0.49	103.43	0.62
have pet at the moment (yes)	-0.39	0.40	-0.98	144.59	0.33
age	-0.01	0.01	-1.45	55.70	0.15
gender (woman)	0.05	0.21	0.24	111.96	0.81
settlement type (city)	-0.05	0.31	-0.17	105.27	0.86
settlement type (municipality)	0.01	0.39	0.02	103.23	0.98
settlement type (county capital)	0.03	0.23	0.11	106.17	0.92
pet (cat):have pet at the moment (yes)	0.36	0.30	1.20	129.01	0.23
pet (other):have pet at the moment (yes)	0.14	0.34	0.41	131.35	0.68
have pet at the moment (yes):gender (woman)	-0.00	0.27	-0.00	132.50	1.00
have pet at the moment (yes):settlement type (city)	0.38	0.39	0.98	132.94	0.33
have pet at the moment (yes):settlement type (municipality)	0.08	0.49	0.17	133.37	0.87
have pet at the moment (yes):settlement type (county capital)	-0.16	0.30	-0.53	133.11	0.59

Random effects:

Group	Parameter	Std. Dev.
ID	(Intercept)	0.42
Residual		0.84

Grouping variable:

Group	# groups	ICC
ID	65	0.20

Sadness
Observations: 194
Type: Mixed effects linear regression
MODEL FIT: AIC = 546.33, BIC = 608.41 Pseudo-R² (fixed effects) = 0.18 Pseudo-R² (total) = 0.52

Fixed effects:

	Est.	S.E.	t val.	d.f.	p
(Intercept)	0.39	0.42	0.95	70.07	0.35
data collection period (2nd)	0.00	0.14	0.01	125.08	0.99
data collection period (3rd)	0.37	0.26	1.45	139.95	0.15
pet (cat)	-0.43	0.25	-1.73	79.47	0.09

pet (other)	-0.01	0.29	-0.05	83.72	0.96
have pet at the moment (yes)	-0.13	0.36	-0.36	135.41	0.72
age	-0.01	0.01	-1.76	55.04	0.08
gender (woman)	0.36	0.23	1.57	89.19	0.12
settlement type (city)	0.18	0.33	0.54	83.82	0.59
settlement type (municipality)	-0.01	0.41	-0.02	82.81	0.98
settlement type (county capital)	0.57	0.25	2.28	83.66	0.03
pet (cat):have pet at the moment (yes)	0.22	0.26	0.85	125.07	0.40
pet (other):have pet at the moment (yes)	-0.15	0.30	-0.48	126.28	0.63
have pet at the moment (yes):gender (woman)	-0.34	0.24	-1.43	126.32	0.15
have pet at the moment (yes):settlement type (city)	-0.53	0.34	-1.53	127.69	0.13
have pet at the moment (yes):settlement type (municipality)	0.05	0.43	0.11	127.10	0.91
have pet at the moment (yes):settlement type (county capital)	-0.11	0.26	-0.40	128.29	0.69

Random effects:

Group	Parameter	Std. Dev.
ID	(Intercept)	0.61
Residual		0.72

Grouping variable:

Group	# groups	ICC
ID	65	0.42

Anxiety

Observations: 194

Type: Mixed effects linear regression

MODEL FIT: AIC = 538.39, BIC = 600.48 Pseudo-R^2 (fixed effects) = 0.20 Pseudo-R^2 (total) = 0.61

Fixed effects:

	Est.	S.E.	t val.	d.f.	p
(Intercept)	-0.06	0.44	-0.13	67.81	0.90
data collection period (2nd)	0.05	0.14	0.35	124.82	0.73
data collection period (3rd)	0.18	0.24	0.73	136.09	0.47
pet (cat)	-0.26	0.26	-1.00	75.02	0.32
pet (other)	0.12	0.30	0.39	78.36	0.70
have pet at the moment (yes)	0.39	0.34	1.16	132.57	0.25
age	-0.01	0.01	-1.53	56.35	0.13
gender (woman)	0.59	0.24	2.46	82.68	0.02
settlement type (city)	0.23	0.35	0.66	78.44	0.51
settlement type (municipality)	-0.16	0.43	-0.37	77.64	0.72
settlement type (county capital)	0.68	0.26	2.59	78.30	0.01
pet (cat):have pet at the moment (yes)	-0.22	0.25	-0.90	124.63	0.37
pet (other):have pet at the moment (yes)	-0.41	0.28	-1.45	125.52	0.15
have pet at the moment (yes):gender (woman)	-0.22	0.22	-1.01	125.52	0.31
have pet at the moment (yes):settlement type (city)	-0.56	0.32	-1.74	126.59	0.08
have pet at the moment (yes):settlement type (municipality)	0.39	0.40	0.97	126.11	0.33
have pet at the moment (yes):settlement type (county capital)	-0.24	0.25	-0.98	127.06	0.33

Random effects:

Group	Parameter	Std. Dev.
ID	(Intercept)	0.69
Residual		0.67

Grouping variable:

Group	# groups	ICC
ID	65	0.52

WHO-5 score
Observations: 193
Type: Mixed effects linear regression
MODEL FIT: AIC = 530.35, BIC = 592.34 Pseudo-R^2 (fixed effects) = 0.09 Pseudo-R^2 (total) = 0.58

Fixed effects:

	Est.	S.E.	t val.	d.f.	p
(Intercept)	0.33	0.45	0.75	66.18	0.46
data collection period (2nd)	-0.12	0.13	-0.93	123.26	0.35
data collection period (3rd)	-0.29	0.24	-1.22	133.73	0.22
pet (cat)	0.33	0.26	1.25	73.30	0.21
pet (other)	-0.12	0.30	-0.40	76.03	0.69
have pet at the moment (yes)	-0.38	0.33	-1.17	130.12	0.25
age	-0.01	0.01	-0.76	55.90	0.45
gender (woman)	-0.05	0.24	-0.21	80.50	0.83
settlement type (city)	0.20	0.35	0.58	77.00	0.57
settlement type (municipality)	0.07	0.44	0.16	75.94	0.87
settlement type (county capital)	-0.25	0.26	-0.95	77.37	0.34
pet (cat):have pet at the moment (yes)	0.34	0.24	1.42	122.56	0.16
pet (other):have pet at the moment (yes)	0.65	0.28	2.34	123.50	0.02
have pet at the moment (yes):gender (woman)	0.12	0.22	0.54	123.50	0.59
have pet at the moment (yes):settlement type (city)	0.24	0.32	0.77	124.50	0.44
have pet at the moment (yes):settlement type (municipality)	0.07	0.39	0.18	124.05	0.86
have pet at the moment (yes):settlement type (county capital)	0.12	0.24	0.51	124.92	0.61

Random effects:

Group	Parameter	Std. Dev.
ID	(Intercept)	0.71
Residual		0.65

Grouping variable:

Group	# groups	ICC
ID	65	0.54

Self-reported health
Observations: 194
Type: Mixed effects linear regression
MODEL FIT: AIC = 526.95, BIC = 589.04 Pseudo-R^2 (fixed effects) = 0.12 Pseudo-R^2 (total) = 0.60

Fixed effects:

	Est.	S.E.	t val.	d.f.	p
(Intercept)	1.40	0.44	3.15	66.03	0.00
data collection period (2nd)	-0.14	0.13	-1.12	124.17	0.27
data collection period (3rd)	-0.08	0.23	-0.36	134.21	0.72
pet (cat)	0.29	0.26	1.09	72.93	0.28
pet (other)	-0.03	0.30	-0.09	75.76	0.93
have pet at the moment (yes)	-0.29	0.32	-0.90	129.80	0.37
age	-0.02	0.01	-2.89	55.66	0.01
gender (woman)	-0.20	0.24	-0.83	79.73	0.41
settlement type (city)	0.05	0.35	0.14	77.37	0.89
settlement type (municipality)	-0.05	0.43	-0.12	75.00	0.90
settlement type (county capital)	-0.06	0.26	-0.23	75.60	0.82
pet (cat):have pet at the moment (yes)	0.19	0.23	0.83	122.93	0.41
pet (other):have pet at the moment (yes)	0.39	0.27	1.45	123.70	0.15
have pet at the moment (yes):gender (woman)	0.03	0.21	0.12	124.16	0.91
have pet at the moment (yes):settlement type (city)	0.28	0.30	0.93	124.22	0.35
have pet at the moment (yes):settlement type (municipality)	0.24	0.38	0.63	124.48	0.53
have pet at the moment (yes):settlement type (county capital)	-0.03	0.23	-0.11	124.43	0.91

Random effects:

Group	Parameter	Std. Dev.
ID	(Intercept)	0.71
Residual		0.64

Grouping variable:

Group	# groups	ICC
ID	65	0.55

GLMM series D

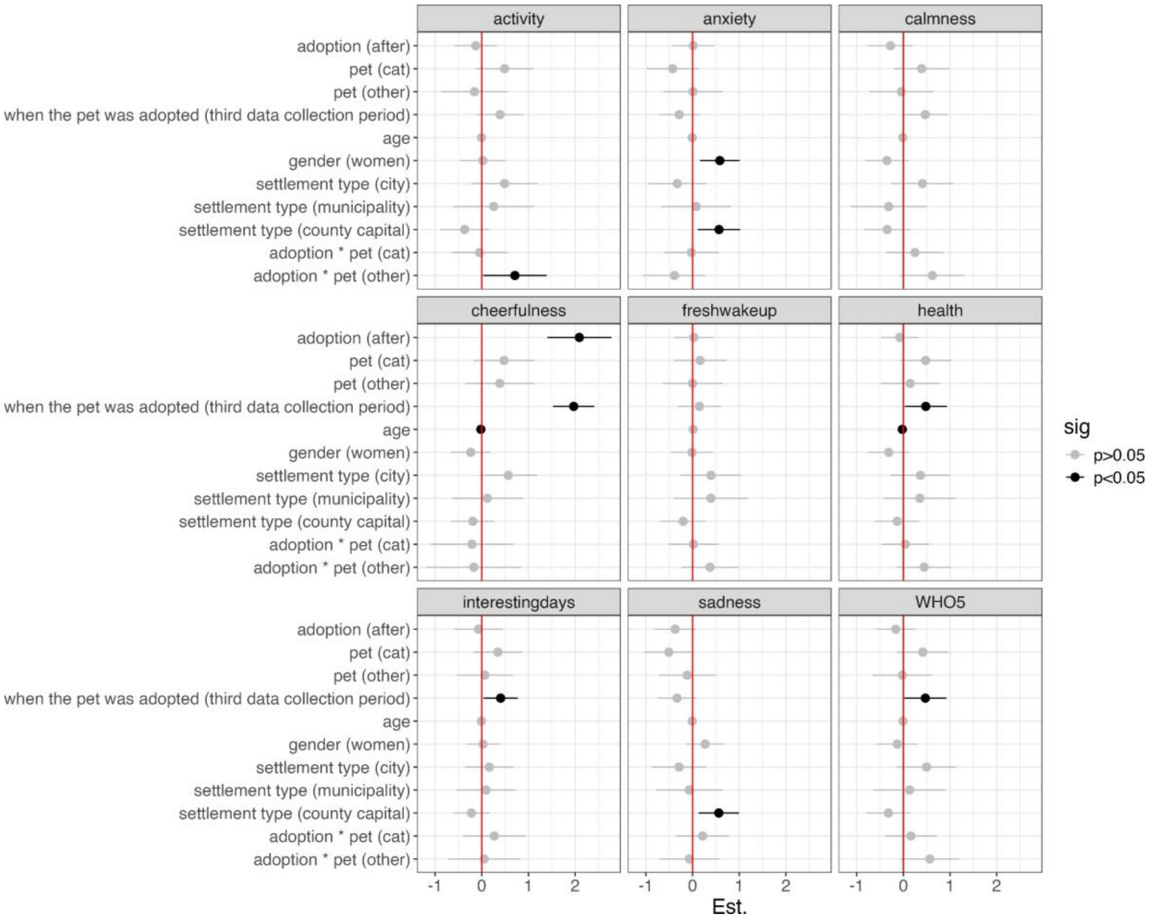

Figure S5. Forest plot of GLMM series D, representing the standardized beta values and the confidence intervals. Separate models for the well-being measurement were carried out. The level of significance is indicated with different colours.

Details of the models

Cheerfulness

Observations: 126

Type: Mixed effects linear regression

MODEL FIT: AIC = 412.51, BIC = 452.22 Pseudo-R² (fixed effects) = 0.60 Pseudo-R² (total) = 0.60

Fixed effects:

	Est.	S.E.	t val.	d.f.	p
(Intercept)	-0.23	0.56	-0.40	114.00	0.69
before or after adoption (after)	2.09	0.35	5.95	114.00	0.00
pet (cat)	0.48	0.33	1.44	114.00	0.15
pet (other)	0.39	0.38	1.02	114.00	0.31
when did adopt (3rd data collection period)	1.97	0.23	8.71	114.00	0.00
age	-0.02	0.01	-2.79	114.00	0.01
sex	-0.24	0.22	-1.07	114.00	0.28
settlement type (city)	0.57	0.32	1.79	114.00	0.08
settlement type (municipality)	0.12	0.39	0.31	114.00	0.76
settlement type (county capital)	-0.19	0.24	-0.81	114.00	0.42
before or after adoption (after) :pet (cat)	-0.21	0.46	-0.46	114.00	0.65
before or after adoption (after) :pet (other)	-0.17	0.52	-0.33	114.00	0.74

Random effects:

Group	Parameter	Std. Dev.
ID	(Intercept)	0.00
Residual		1.08

Grouping variable:

Group	# groups	ICC
ID	63	0.00

Calmness

Observations: 126

Type: Mixed effects linear regression

MODEL FIT: AIC = 375.05, BIC = 414.76 Pseudo-R^2 (fixed effects) = 0.13 Pseudo-R^2 (total) = 0.50

Fixed effects:

	Est.	S.E.	t val.	d.f.	p
(Intercept)	0.74	0.59	1.26	58.75	0.21
before or after adoption (after)	-0.27	0.24	-1.14	60.00	0.26
pet (cat)	0.39	0.30	1.30	89.26	0.20
pet (other)	-0.04	0.35	-0.12	89.15	0.90
when did adopt (3rd data collection period)	0.47	0.24	1.93	54.00	0.06
age	-0.01	0.01	-1.42	54.00	0.16
sex	-0.35	0.24	-1.48	54.00	0.14
settlement type (city)	0.41	0.34	1.19	54.00	0.24
settlement type (municipality)	-0.32	0.42	-0.76	54.00	0.45
settlement type (county capital)	-0.35	0.25	-1.38	54.00	0.17
before or after adoption (after) :pet (cat)	0.25	0.31	0.80	60.00	0.43
before or after adoption (after) :pet (other)	0.62	0.36	1.75	60.00	0.08

Random effects:

Group	Parameter	Std. Dev.
ID	(Intercept)	0.64
Residual		0.74

Grouping variable:

Group	# groups	ICC
ID	63	0.43

Activity

Observations: 126

Type: Mixed effects linear regression

MODEL FIT: AIC = 378.08, BIC = 417.78 Pseudo-R^2 (fixed effects) = 0.15 Pseudo-R^2 (total) = 0.57

Fixed effects:

	Est.	S.E.	t val.	d.f.	p
(Intercept)	-0.15	0.62	-0.25	58.04	0.81
before or after adoption (after)	-0.13	0.23	-0.56	60.00	0.58
pet (cat)	0.49	0.31	1.55	84.78	0.12
pet (other)	-0.16	0.36	-0.44	84.67	0.66
when did adopt (3rd data collection period)	0.39	0.26	1.52	54.00	0.13
age	-0.01	0.01	-0.73	54.00	0.47
sex	0.02	0.25	0.10	54.00	0.92
settlement type (city)	0.49	0.36	1.35	54.00	0.18
settlement type (municipality)	0.26	0.44	0.58	54.00	0.56
settlement type (county capital)	-0.37	0.27	-1.36	54.00	0.18
before or after adoption (after) :pet (cat)	-0.05	0.30	-0.15	60.00	0.88
before or after adoption (after) :pet (other)	0.71	0.35	2.05	60.00	0.04

Random effects:

Group	Parameter	Std. Dev.
ID	(Intercept)	0.71
Residual		0.72

Grouping variable:

Group	# groups	ICC
ID	63	0.49

Feeling fresh and rested

Observations: 126

Type: Mixed effects linear regression

MODEL FIT: AIC = 356.44, BIC = 396.15 Pseudo-R^2 (fixed effects) = 0.13 Pseudo-R^2 (total) = 0.57

Fixed effects:

	Est.	S.E.	t val.	d.f.	p
(Intercept)	-0.93	0.57	-1.64	57.90	0.11
before or after adoption (after)	0.03	0.21	0.12	60.00	0.90
pet (cat)	0.16	0.29	0.56	83.86	0.58
pet (other)	-0.00	0.33	-0.00	83.76	1.00
when did adopt (3rd data collection period)	0.15	0.24	0.61	54.00	0.54
age	0.01	0.01	1.90	54.00	0.06
sex	-0.01	0.23	-0.05	54.00	0.96
settlement type (city)	0.39	0.33	1.19	54.00	0.24
settlement type (municipality)	0.39	0.40	0.97	54.00	0.34
settlement type (county capital)	-0.20	0.25	-0.82	54.00	0.42
before or after adoption (after) :pet (cat)	0.02	0.27	0.07	60.00	0.95
before or after adoption (after) :pet (other)	0.37	0.31	1.20	60.00	0.24

Random effects:

Group	Parameter	Std. Dev.
ID	(Intercept)	0.66
Residual		0.65

Grouping variable:

Group	# groups	ICC
ID	63	0.50

Having interesting days

Observations: 126

Type: Mixed effects linear regression

MODEL FIT: AIC = 359.59, BIC = 399.30 Pseudo-R^2 (fixed effects) = 0.13 Pseudo-R^2 (total) = 0.22

Fixed effects:

	Est.	S.E.	t val.	d.f.	p
(Intercept)	-0.14	0.46	-0.31	63.60	0.76
before or after adoption (after)	-0.07	0.27	-0.26	60.00	0.79
pet (cat)	0.34	0.27	1.29	109.08	0.20
pet (other)	0.07	0.30	0.22	108.99	0.83
when did adopt (3rd data collection period)	0.40	0.19	2.14	54.00	0.04
age	-0.01	0.01	-1.21	54.00	0.23
sex	0.03	0.18	0.14	54.00	0.89
settlement type (city)	0.16	0.27	0.60	54.00	0.55

settlement type (municipality)	0.09	0.32	0.28	54.00	0.78
settlement type (county capital)	-0.22	0.20	-1.13	54.00	0.27
before or after adoption (after) :pet (cat)	0.27	0.34	0.78	60.00	0.44
before or after adoption (after) :pet (other)	0.05	0.39	0.13	60.00	0.89

Random effects:

Group	Parameter	Std. Dev.
ID	(Intercept)	0.27
Residual		0.82

Grouping variable:

Group	# groups	ICC
ID	63	0.10

Sadness

Observations: 126

Type: Mixed effects linear regression

MODEL FIT: AIC = 350.21, BIC = 389.92 Pseudo-R^2 (fixed effects) = 0.23 Pseudo-R^2 (total) = 0.52

Fixed effects:

	Est.	S.E.	t val.	d.f.	p
(Intercept)	0.37	0.51	0.72	59.37	0.47
before or after adoption (after)	-0.37	0.22	-1.69	60.00	0.10
pet (cat)	-0.51	0.27	-1.90	92.82	0.06
pet (other)	-0.12	0.31	-0.38	92.70	0.70
when did adopt (3rd data collection period)	-0.33	0.21	-1.57	54.00	0.12
age	-0.01	0.01	-1.04	54.00	0.30
sex	0.27	0.21	1.31	54.00	0.19
settlement type (city)	-0.29	0.30	-0.98	54.00	0.33
settlement type (municipality)	-0.07	0.36	-0.19	54.00	0.85
settlement type (county capital)	0.56	0.22	2.56	54.00	0.01
before or after adoption (after) :pet (cat)	0.22	0.29	0.75	60.00	0.45
before or after adoption (after) :pet (other)	-0.07	0.33	-0.21	60.00	0.84

Random effects:

Group	Parameter	Std. Dev.
ID	(Intercept)	0.53
Residual		0.68

Grouping variable:

Group	# groups	ICC
ID	63	0.37

Anxiety

Observations: 126

Type: Mixed effects linear regression

MODEL FIT: AIC = 360.77, BIC = 400.47 Pseudo-R^2 (fixed effects) = 0.24 Pseudo-R^2 (total) = 0.53

Fixed effects:

	Est.	S.E.	t val.	d.f.	p
(Intercept)	-0.40	0.54	-0.75	59.25	0.46
before or after adoption (after)	0.01	0.23	0.06	60.00	0.95
pet (cat)	-0.43	0.28	-1.52	92.15	0.13
pet (other)	0.01	0.32	0.02	92.04	0.98
when did adopt (3rd data collection period)	-0.29	0.22	-1.29	54.00	0.20
age	-0.01	0.01	-0.95	54.00	0.34
sex	0.58	0.22	2.69	54.00	0.01

settlement type (city)	-0.33	0.31	-1.04	54.00	0.30
settlement type (municipality)	0.08	0.38	0.20	54.00	0.84
settlement type (county capital)	0.57	0.23	2.44	54.00	0.02
before or after adoption (after) :pet (cat)	-0.02	0.30	-0.08	60.00	0.93
before or after adoption (after) :pet (other)	-0.39	0.34	-1.15	60.00	0.26

Random effects:

Group	Parameter	Std. Dev.
ID	(Intercept)	0.56
Residual		0.71

Grouping variable:

Group	# groups	ICC
ID	63	0.38

WHO5 score

Observations: 126

Type: Mixed effects linear regression

MODEL FIT: AIC = 355.07, BIC = 394.78 Pseudo-R^2 (fixed effects) = 0.16 Pseudo-R^2 (total) = 0.55

Fixed effects:

	Est.	S.E.	t val.	d.f.	p
(Intercept)	0.10	0.55	0.17	58.30	0.86
before or after adoption (after)	-0.16	0.22	-0.76	60.00	0.45
pet (cat)	0.42	0.28	1.48	86.48	0.14
pet (other)	-0.02	0.32	-0.07	86.37	0.95
when did adopt (3rd data collection period)	0.47	0.23	2.06	54.00	0.04
age	-0.01	0.01	-1.01	54.00	0.32
sex	-0.13	0.22	-0.59	54.00	0.56
settlement type (city)	0.50	0.32	1.54	54.00	0.13
settlement type (municipality)	0.13	0.39	0.34	54.00	0.73
settlement type (county capital)	-0.32	0.24	-1.34	54.00	0.18
before or after adoption (after) :pet (cat)	0.16	0.28	0.57	60.00	0.57
before or after adoption (after) :pet (other)	0.57	0.32	1.78	60.00	0.08

Random effects:

Group	Parameter	Std. Dev.
ID	(Intercept)	0.62
Residual		0.66

Grouping variable:

Group	# groups	ICC
ID	63	0.47

Self-reported health

Observations: 126

Type: Mixed effects linear regression

MODEL FIT: AIC = 348.25, BIC = 387.96 Pseudo-R^2 (fixed effects) = 0.20 Pseudo-R^2 (total) = 0.62

Fixed effects:

	Est.	S.E.	t val.	d.f.	p
(Intercept)	1.32	0.55	2.38	57.70	0.02
before or after adoption (after)	-0.08	0.20	-0.38	60.00	0.71
pet (cat)	0.48	0.28	1.70	82.48	0.09
pet (other)	0.15	0.32	0.46	82.38	0.65
when did adopt (3rd data collection period)	0.48	0.23	2.07	54.00	0.04
age	-0.02	0.01	-3.25	54.00	0.00
sex	-0.31	0.23	-1.39	54.00	0.17
settlement type (city)	0.36	0.33	1.12	54.00	0.27

settlement type (municipality)	0.35	0.40	0.89	54.00	0.38
settlement type (county capital)	-0.13	0.24	-0.55	54.00	0.59
before or after adoption (after) :pet (cat)	0.04	0.26	0.16	60.00	0.88
before or after adoption (after) :pet (other)	0.45	0.30	1.50	60.00	0.14

Random effects:

Group	Parameter	Std. Dev.
ID	(Intercept)	0.65
Residual		0.62

Grouping variable:

Group	# groups	ICC
ID	63	0.52

Q2. Does losing a pet affect well-being?

GLMM series E

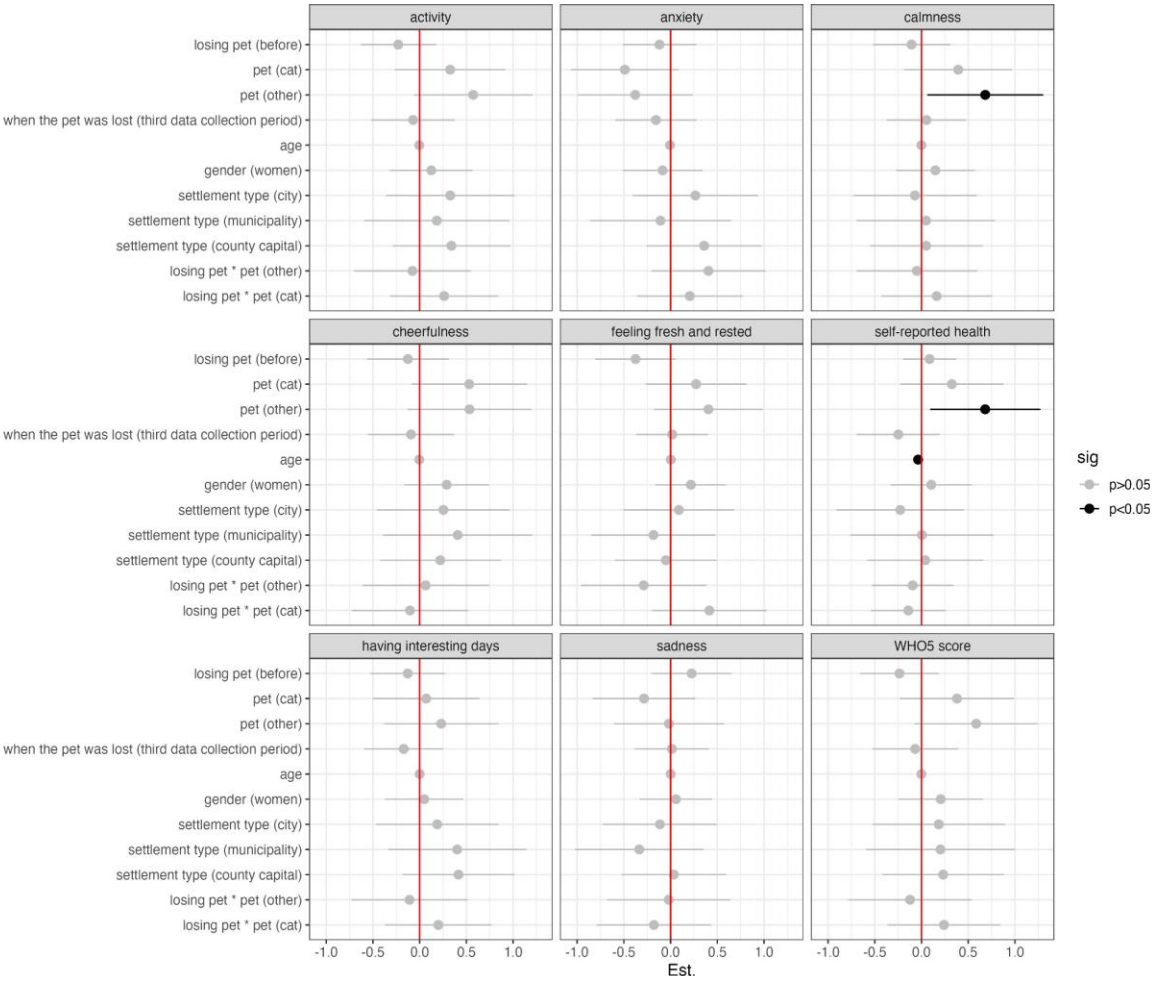

Figure S6. Forest plot of GLMM series E, representing the standardized beta values and the confidence intervals. Separate models for well-being measurements were carried out. The level of significance is indicated with different colours.

Details of the models

Cheerfulness

Observations: 150

Type: Mixed effects linear regression

MODEL FIT: AIC = 473.27, BIC = 515.42 Pseudo-R^2 (fixed effects) = 0.07 Pseudo-R^2 (total) = 0.46

Fixed effects:

	Est.	S.E.	t val.	d.f.	p
(Intercept)	-0.33	0.52	-0.63	72.23	0.53
pet losing state (not lost yet)	-0.13	0.22	-0.57	72.00	0.57
pet (cat)	0.53	0.31	1.68	107.32	0.09
pet (other)	0.53	0.34	1.58	108.25	0.12
when the pet was lost (3rd data collection period)	-0.09	0.24	-0.40	66.00	0.69
age	-0.00	0.01	-0.49	66.00	0.63
gender (woman)	0.29	0.23	1.25	66.00	0.22
settlement type (city)	0.25	0.36	0.70	66.00	0.49
settlement type (municipality)	0.41	0.41	1.00	66.00	0.32
settlement type (county capital)	0.22	0.33	0.67	66.00	0.51
pet losing state (not lost yet) : pet (cat)	-0.10	0.32	-0.33	72.00	0.75
pet losing state (not lost yet) :pet (other)	0.06	0.34	0.18	72.00	0.85

Random effects:

Group	Parameter	Std. Dev.
ID	(Intercept)	0.71
Residual		0.83

Grouping variable:

Group	# groups	ICC
ID	75	0.42

Calmness

Observations: 150

Type: Mixed effects linear regression

MODEL FIT: AIC = 456.65, BIC = 498.80 Pseudo-R² (fixed effects) = 0.07 Pseudo-R² (total) = 0.44

Fixed effects:

	Est.	S.E.	t val.	d.f.	p
(Intercept)	-0.27	0.49	-0.55	72.56	0.59
pet losing state (not lost yet)	-0.11	0.21	-0.50	72.00	0.62
pet (cat)	0.39	0.29	1.33	109.08	0.19
pet (other)	0.68	0.32	2.16	110.03	0.03
when the pet was lost (3rd data collection period)	0.05	0.22	0.24	66.00	0.81
age	-0.00	0.01	-0.16	66.00	0.87
gender (woman)	0.15	0.22	0.69	66.00	0.49
settlement type (city)	-0.07	0.34	-0.21	66.00	0.83
settlement type (municipality)	0.05	0.38	0.13	66.00	0.90
settlement type (county capital)	0.05	0.31	0.16	66.00	0.87
pet losing state (not lost yet) :pet (cat)	0.16	0.30	0.54	72.00	0.59
pet losing state (not lost yet) :pet (other)	-0.05	0.33	-0.15	72.00	0.88

Random effects:

Group	Parameter	Std. Dev.
ID	(Intercept)	0.64
Residual		0.79

Grouping variable:

Group	# groups	ICC
ID	75	0.40

Activity

Observations: 150

Type: Mixed effects linear regression

MODEL FIT: AIC = 458.40, BIC = 500.55 Pseudo-R² (fixed effects) = 0.06 Pseudo-R² (total) = 0.49

Fixed effects:

	Est.	S.E.	t val.	d.f.	p
(Intercept)	-0.22	0.51	-0.44	71.67	0.66
pet losing state (not lost yet)	-0.23	0.21	-1.11	72.00	0.27
pet (cat)	0.33	0.30	1.08	104.16	0.28
pet (other)	0.57	0.32	1.76	105.05	0.08
when the pet was lost (3rd data collection period)	-0.07	0.23	-0.31	66.00	0.76
age	-0.00	0.01	-0.46	66.00	0.65
gender (woman)	0.12	0.23	0.55	66.00	0.58
settlement type (city)	0.33	0.35	0.93	66.00	0.36
settlement type (municipality)	0.18	0.40	0.46	66.00	0.65
settlement type (county capital)	0.34	0.32	1.05	66.00	0.30

pet losing state (not lost yet) : pet (cat)	0.26	0.29	0.89	72.00	0.38
pet losing state (not lost yet) :pet (other)	-0.08	0.32	-0.24	72.00	0.81

Random effects:

Group	Parameter	Std. Dev.
ID	(Intercept)	0.71
Residual		0.77

Grouping variable:

Group	# groups	ICC
ID	75	0.46

Feeling fresh and rested

Observations: 149

Type: Mixed effects linear regression

MODEL FIT: AIC = 443.86, BIC = 485.91 Pseudo-R^2 (fixed effects) = 0.10 Pseudo-R^2 (total) = 0.34

Fixed effects:

	Est.	S.E.	t val.	d.f.	p
(Intercept)	-0.22	0.44	-0.49	74.62	0.62
pet losing state (not lost yet)	-0.37	0.22	-1.71	71.48	0.09
pet (cat)	0.27	0.27	1.00	118.87	0.32
pet (other)	0.41	0.30	1.36	120.64	0.18
when the pet was lost (3rd data collection period)	0.02	0.20	0.09	65.81	0.93
age	0.00	0.01	0.32	65.82	0.75
gender (woman)	0.22	0.19	1.12	66.20	0.27
settlement type (city)	0.09	0.30	0.29	66.78	0.77
settlement type (municipality)	-0.18	0.34	-0.54	66.33	0.59
settlement type (county capital)	-0.05	0.28	-0.18	67.09	0.86
pet losing state (not lost yet) : pet (cat)	0.42	0.31	1.33	71.48	0.19
pet losing state (not lost yet) :pet (other)	-0.29	0.34	-0.84	72.46	0.40

Random effects:

Group	Parameter	Std. Dev.
ID	(Intercept)	0.49
Residual		0.82

epo

Grouping variable:

Group	# groups	ICC
ID	75	0.27

Having interesting days

Observations: 150

Type: Mixed effects linear regression

MODEL FIT: AIC = 450.53, BIC = 492.67 Pseudo-R^2 (fixed effects) = 0.03 Pseudo-R^2 (total) = 0.44

Fixed effects:

	Est.	S.E.	t val.	d.f.	p
(Intercept)	-0.10	0.48	-0.21	72.14	0.83
pet losing state (not lost yet)	-0.13	0.20	-0.63	72.00	0.53
pet (cat)	0.07	0.29	0.24	106.82	0.81
pet (other)	0.23	0.31	0.74	107.75	0.46
when the pet was lost (3rd data collection period)	-0.17	0.22	-0.79	66.00	0.44
age	0.00	0.01	0.09	66.00	0.93

gender (woman)	0.05	0.21	0.23	66.00	0.82
settlement type (city)	0.19	0.33	0.56	66.00	0.58
settlement type (municipality)	0.40	0.38	1.07	66.00	0.29
settlement type (county capital)	0.42	0.31	1.36	66.00	0.18
pet losing state (not lost yet) : pet (cat)	0.20	0.29	0.68	72.00	0.50
pet losing state (not lost yet) : pet (other)	-0.11	0.32	-0.34	72.00	0.73

Random effects:

Group	Parameter	Std. Dev.
ID	(Intercept)	0.66
Residual		0.76

Grouping variable:

Group	# groups	ICC
ID	75	0.42

Sadness

Observations: 149

Type: Mixed effects linear regression

MODEL FIT: AIC = 446.50, BIC = 488.56 Pseudo-R^2 (fixed effects) = 0.06 Pseudo-R^2 (total) = 0.35

Fixed effects:

	Est.	S.E.	t val.	d.f.	p
(Intercept)	-0.05	0.45	-0.12	73.99	0.91
pet losing state (not lost yet)	0.23	0.22	1.04	71.43	0.30
pet (cat)	-0.28	0.28	-1.02	116.10	0.31
pet (other)	-0.02	0.30	-0.06	117.19	0.95
when the pet was lost (3rd data collection period)	0.01	0.20	0.07	66.08	0.95
age	0.00	0.01	0.19	66.46	0.85
gender (woman)	0.06	0.20	0.29	65.88	0.77
settlement type (city)	-0.11	0.31	-0.37	66.86	0.72
settlement type (municipality)	-0.33	0.35	-0.95	67.13	0.34
settlement type (county capital)	0.03	0.29	0.12	67.21	0.90
pet losing state (not lost yet) : pet (cat)	-0.18	0.31	-0.57	72.04	0.57
pet losing state (not lost yet) :pet (other)	-0.02	0.34	-0.06	71.43	0.95

Random effects:

Group	Parameter	Std. Dev.
ID	(Intercept)	0.54
Residual		0.81

Grouping variable:

Group	# groups	ICC
ID	75	0.30

Anxiety

Observations: 149

Type: Mixed effects linear regression

MODEL FIT: AIC = 447.84, BIC = 489.90 Pseudo-R^2 (fixed effects) = 0.08 Pseudo-R^2 (total) = 0.49

Fixed effects:

	Est.	S.E.	t val.	d.f.	p
(Intercept)	0.52	0.49	1.05	71.77	0.30
pet losing state (not lost yet)	-0.12	0.20	-0.59	71.42	0.56

pet (cat)	-0.49	0.29	-1.67	104.37	0.10
pet (other)	-0.38	0.32	-1.20	105.41	0.23
when the pet was lost (3rd data collection period)	-0.16	0.22	-0.71	66.17	0.48
age	-0.01	0.01	-0.83	66.50	0.41
gender (woman)	-0.09	0.22	-0.39	66.00	0.70
settlement type (city)	0.27	0.34	0.78	66.85	0.44
settlement type (municipality)	-0.11	0.39	-0.28	67.08	0.78
settlement type (county capital)	0.36	0.31	1.15	67.15	0.26
pet losing state (not lost yet) : pet (cat)	0.21	0.29	0.71	71.94	0.48
pet losing state (not lost yet) :pet (other)	0.41	0.31	1.30	71.42	0.20

Random effects:

Group	Parameter	Std. Dev.
ID	(Intercept)	0.68
Residual		0.75

Grouping variable:

Group	# groups	ICC
ID	75	0.45

WHO-5 score
Observations: 149
Type: Mixed effects linear regression
MODEL FIT: AIC = 464.84, BIC = 506.90 Pseudo-R^2 (fixed effects) = 0.06 Pseudo-R^2 (total) = 0.47

Fixed effects:

	Est.	S.E.	t val.	d.f.	p
(Intercept)	-0.27	0.52	-0.51	71.87	0.61
pet losing state (not lost yet)	-0.24	0.22	-1.10	71.44	0.28
pet (cat)	0.38	0.31	1.22	105.11	0.22
pet (other)	0.58	0.34	1.74	107.20	0.08
when the pet was lost (3rd data collection period)	-0.07	0.23	-0.29	65.94	0.77
age	-0.00	0.01	-0.18	65.94	0.85
gender (woman)	0.21	0.23	0.89	66.27	0.38
settlement type (city)	0.18	0.36	0.51	66.77	0.61
settlement type (municipality)	0.20	0.41	0.50	66.38	0.62
settlement type (county capital)	0.23	0.33	0.70	67.04	0.49
pet losing state (not lost yet) : pet (cat)	0.24	0.31	0.78	71.44	0.44
pet losing state (not lost yet) :pet (other)	-0.12	0.34	-0.36	72.28	0.72

Random effects:

Group	Parameter	Std. Dev.
ID	(Intercept)	0.71
Residual		0.81

Grouping variable:

Group	# groups	ICC
ID	75	0.44

Self-reported health
Observations: 150
Type: Mixed effects linear regression
MODEL FIT: AIC = 405.17, BIC = 447.32 Pseudo-R^2 (fixed effects) = 0.27 Pseudo-R^2 (total) = 0.77

Fixed effects:

	Est.	S.E.	t val.	d.f.	p
(Intercept)	1.71	0.50	3.44	68.83	0.00
pet losing state (not lost yet)	0.09	0.14	0.60	72.00	0.55
pet (cat)	0.32	0.28	1.16	86.18	0.25
pet (other)	0.68	0.30	2.26	86.72	0.03
when the pet was lost (3rd data collection period)	-0.25	0.23	-1.10	66.00	0.28
age	-0.04	0.01	-4.81	66.00	0.00
gender (woman)	0.10	0.22	0.46	66.00	0.64
settlement type (city)	-0.23	0.35	-0.66	66.00	0.51
settlement type (municipality)	0.00	0.39	0.01	66.00	0.99
settlement type (county capital)	0.04	0.32	0.12	66.00	0.90
pet losing state (not lost yet) : pet (cat)	-0.14	0.21	-0.69	72.00	0.49
pet losing state (not lost yet) :pet (other)	-0.09	0.22	-0.42	72.00	0.67

Random effects:

Group	Parameter	Std. Dev.
ID	(Intercept)	0.80
Residual		0.54

Grouping variable:

Group	# groups	ICC
ID	75	0.69

Q3. Is there a difference between the well-being of participants who were about to acquire a pet and those who have no pets?

Table S3 *The difference between future pet owner and non-pet owner, Dunn post hoc test, done after the significant Kruskal-Wallis tests.*

variable	group1	group2	n1	n2	statistic	p	p.adj	p.adj.signif
WHO5 score	cat	dog	9	4	-1.0501391	0.2936542	1.0000000	ns
	cat	none	9	1320	-0.6374388	0.5238391	1.0000000	ns
	cat	other	9	7	-2.0690786	0.0385387	0.1926936	ns
	dog	none	4	1320	0.8344413	0.4040323	1.0000000	ns
	dog	other	4	7	-0.6567862	0.5113184	1.0000000	ns
	none	other	1320	7	-2.1888938	0.0286046	0.1716273	ns
**activity**	cat	dog	9	4	-1.5628040	0.1180987	0.4723948	ns
	cat	none	9	1326	-1.1581548	0.2468009	0.7404026	ns
	**cat**	**other**	**9**	**7**	**-2.8661292**	**0.0041552**	**0.0249315**	*****
	dog	none	4	1326	1.1018770	0.2705152	0.7404026	ns
	dog	other	4	7	-0.8061241	0.4201713	0.7404026	ns
	**none**	**other**	**1326**	**7**	**-2.7892959**	**0.0052823**	**0.0264114**	*****
anxiety	cat	dog	9	4	-0.7357550	0.4618798	0.9237597	ns
	cat	none	9	1328	0.2073098	0.8357679	0.9237597	ns
	cat	other	9	7	1.4045668	0.1601501	0.6406006	ns
	dog	none	4	1328	1.0214044	0.3070629	0.9211887	ns
	dog	other	4	7	1.8347155	0.0665479	0.3992871	ns
	none	other	1328	7	1.6848724	0.0920132	0.4600659	ns
calmness	cat	dog	9	4	-0.3052246	0.7601951	1.0000000	ns
	cat	none	9	1328	-0.5950803	0.5517898	1.0000000	ns
	cat	other	9	7	-1.4893133	0.1364049	0.8184293	ns
	dog	none	4	1328	-0.0311810	0.9751252	1.0000000	ns
	dog	other	4	7	-0.9048204	0.3655605	1.0000000	ns
	none	other	1328	7	-1.4553337	0.1455769	0.8184293	ns
cheerfulness	cat	dog	9	4	-0.1482631	0.8821351	1.0000000	ns
	cat	none	9	1327	-0.1708463	0.8643447	1.0000000	ns
	cat	other	9	7	-0.6378185	0.5235918	1.0000000	ns
	dog	none	4	1327	0.0638109	0.9491208	1.0000000	ns
	dog	other	4	7	-0.3706788	0.7108767	1.0000000	ns
	none	other	1327	7	-0.6974054	0.4855491	1.0000000	ns
feeling fresh and rested	cat	dog	9	4	-1.1667865	0.2432966	0.9731864	ns
	cat	none	9	1324	-0.9829377	0.3256381	0.9769143	ns
	cat	other	9	7	-2.0468453	0.0406733	0.2440396	ns
	dog	none	4	1324	0.7436651	0.4570791	0.9769143	ns
	dog	other	4	7	-0.5270749	0.5981416	0.9769143	ns
	none	other	1324	7	-1.8544205	0.0636790	0.3183951	ns
having interesting day	cat	dog	9	4	-1.1984304	0.2307495	0.8272816	ns
	cat	none	9	1326	-0.2632202	0.7923808	1.0000000	ns
	cat	other	9	7	-1.5811434	0.1138453	0.5692263	ns
	dog	none	4	1326	1.2623569	0.2068204	0.8272816	ns
	dog	other	4	7	-0.1222980	0.9026630	1.0000000	ns
	none	other	1326	7	-1.8703360	0.0614372	0.3686231	ns
**sadness**	cat	dog	9	4	1.0964945	0.2728624	1.0000000	ns
	cat	none	9	1329	0.2928578	0.7696309	1.0000000	ns

	cat	other	9	7	2.2089049	0.0271813	0.1359063	ns
	dog	none	4	1329	-1.1202393	0.2626118	1.0000000	Ns
	dog	other	4	7	0.7247678	0.4685945	1.0000000	ns
	none	other	1329	7	2.6790112	0.0073840	0.0443040	*
self-reported health	cat	dog	9	4	-0.6102490	0.5416969	1.0000000	ns
	cat	none	9	1333	-1.2744255	0.2025127	1.0000000	ns
	cat	other	9	7	-1.0540870	0.2918431	1.0000000	ns
	dog	none	4	1333	-0.1188741	0.9053751	1.0000000	ns
	dog	other	4	7	-0.2624447	0.7929786	1.0000000	ns
	none	other	1333	7	-0.2769974	0.7817821	1.0000000	ns

Detailed results of the bootstrapped GLMM series

The following figures represent forest plots of GLMM series A-E, after bootstrapping with case resampling (B=1000). The values represent the standardized beta values, the thick line the 95% confidence intervals and the thin lines the 66% confidence intervals.

GLMM series A

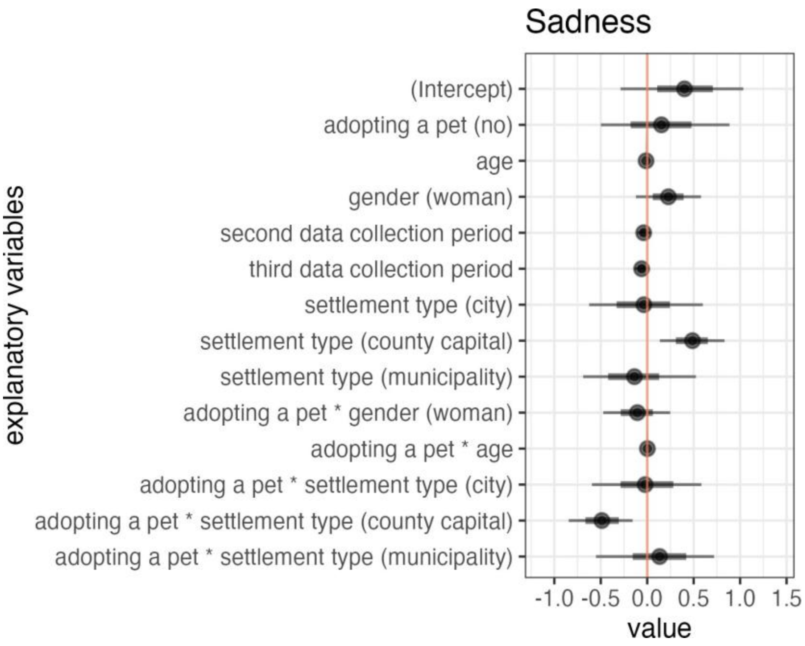

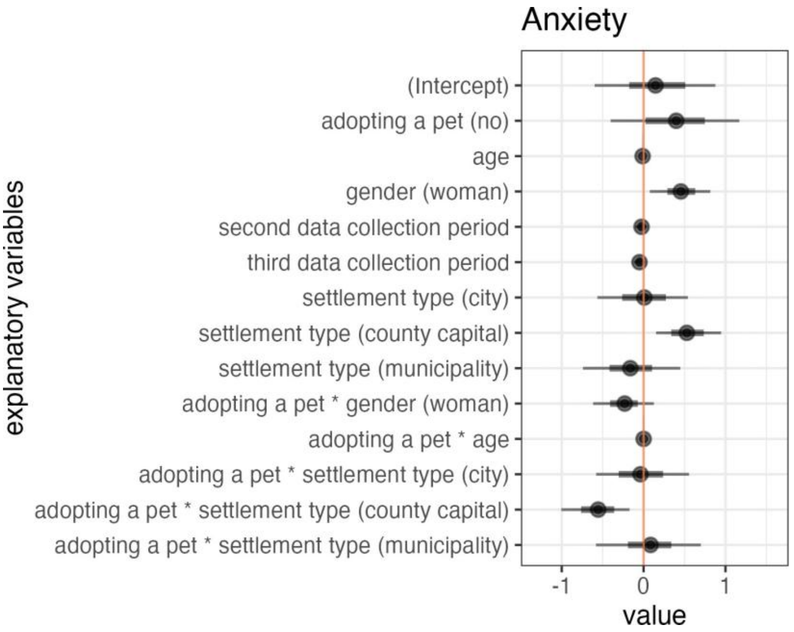

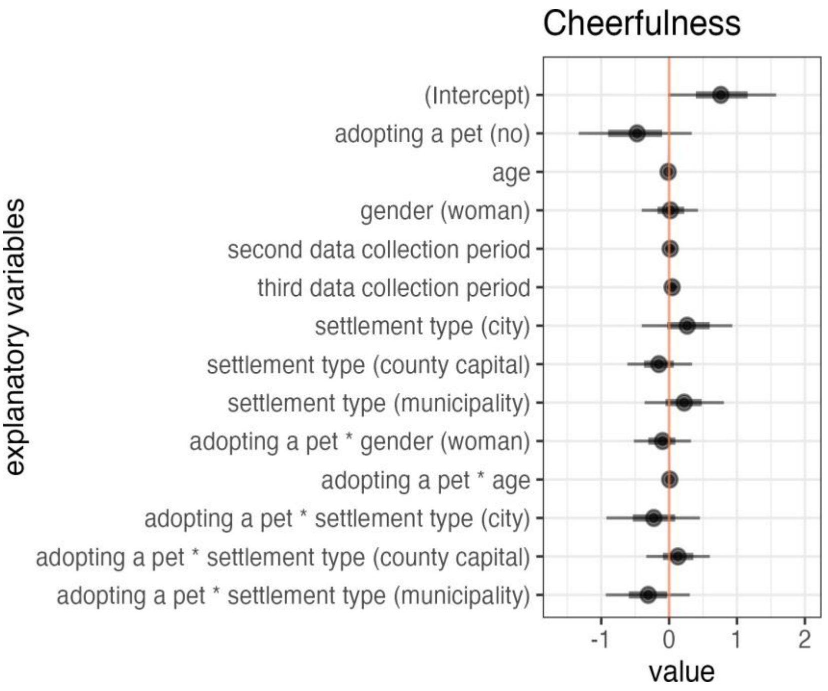
Cheerfulness
(Intercept)
adopting a pet (no)
age
gender (woman)
second data collection period
third data collection period
settlement type (city)
settlement type (county capital)
settlement type (municipality)
adopting a pet * gender (woman)
adopting a pet * age
adopting a pet * settlement type (city)
adopting a pet * settlement type (county capital)
adopting a pet * settlement type (municipality)
explanatory variables
-1
0
1
2
value

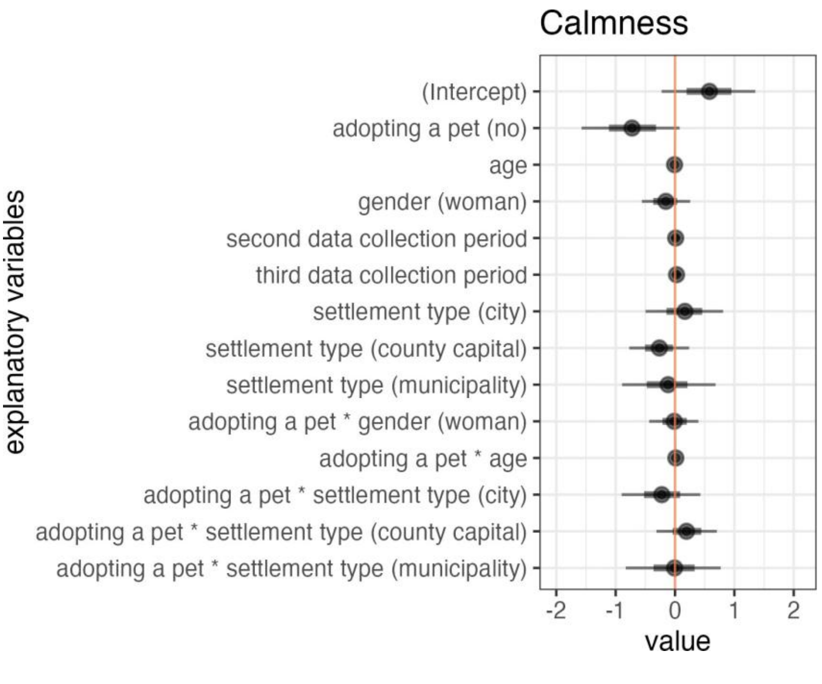
Calmness
(Intercept)
adopting a pet (no)
age
gender (woman)
second data collection period
third data collection period
settlement type (city)
settlement type (county capital)
settlement type (municipality)
adopting a pet * gender (woman)
adopting a pet * age
adopting a pet * settlement type (city)
adopting a pet * settlement type (county capital)
adopting a pet * settlement type (municipality)
explanatory variables
-2
-1
0
1
2
value

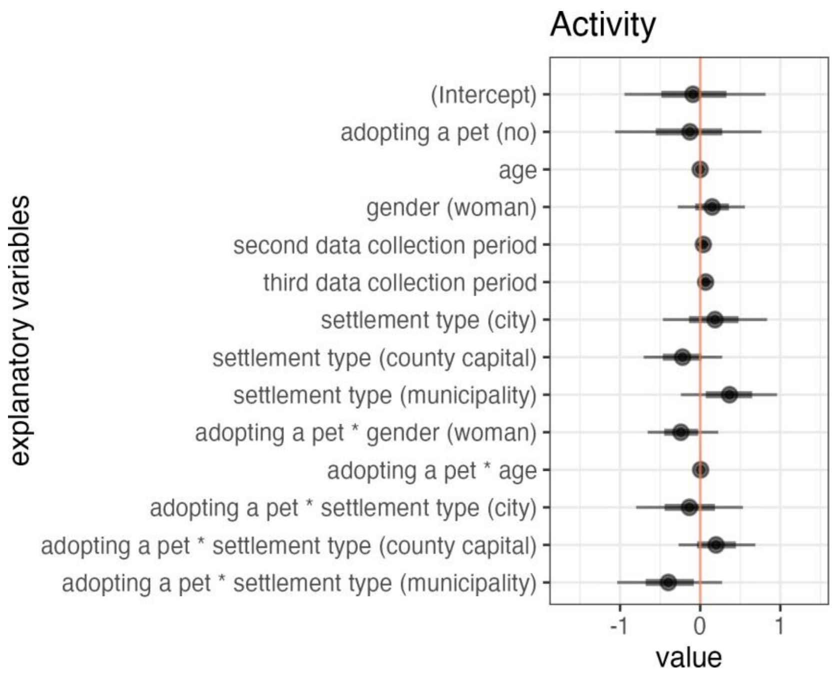
Activity
(Intercept)
adopting a pet (no)
age
gender (woman)
second data collection period
third data collection period
settlement type (city)
settlement type (county capital)
settlement type (municipality)
adopting a pet * gender (woman)
adopting a pet * age
adopting a pet * settlement type (city)
adopting a pet * settlement type (county capital)
adopting a pet * settlement type (municipality)
explanatory variables
-1
0
1
value

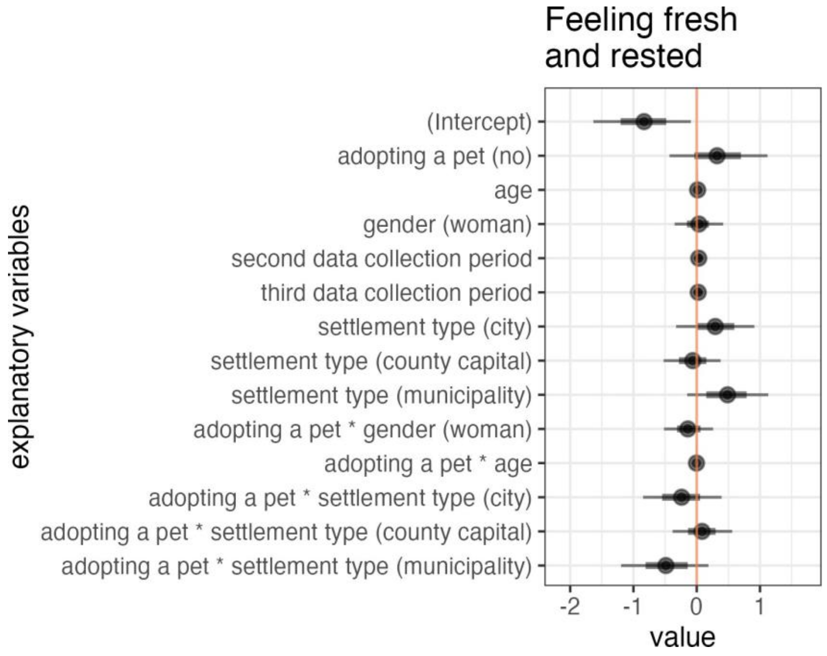
Feeling fresh and rested
(Intercept)
adopting a pet (no)
age
gender (woman)
second data collection period
third data collection period
settlement type (city)
settlement type (county capital)
settlement type (municipality)
adopting a pet * gender (woman)
adopting a pet * age
adopting a pet * settlement type (city)
adopting a pet * settlement type (county capital)
adopting a pet * settlement type (municipality)
explanatory variables
-2
-1
0
1
value

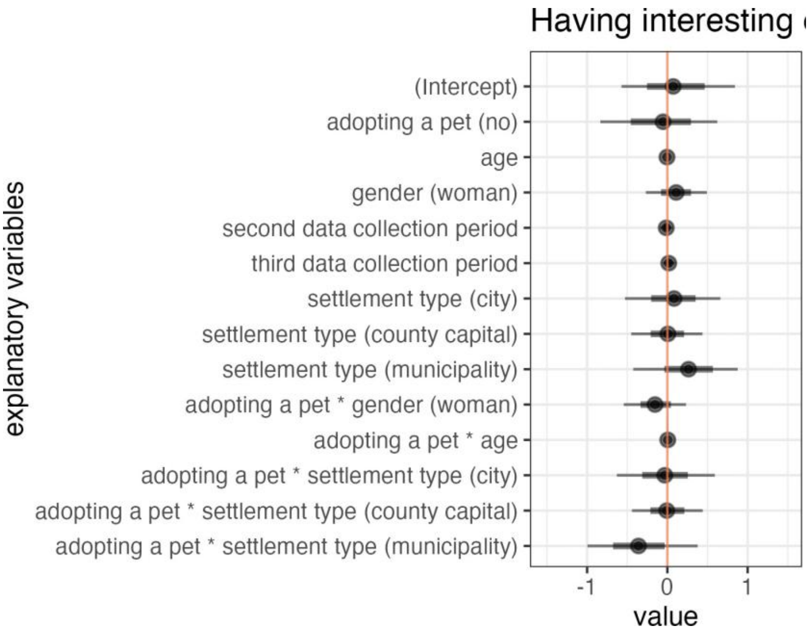
Having interesting
(Intercept)
adopting a pet (no)
age
gender (woman)
second data collection period
third data collection period
settlement type (city)
settlement type (county capital)
settlement type (municipality)
adopting a pet * gender (woman)
adopting a pet * age
adopting a pet * settlement type (city)
adopting a pet * settlement type (county capital)
adopting a pet * settlement type (municipality)
explanatory variables
-1
0
1
value

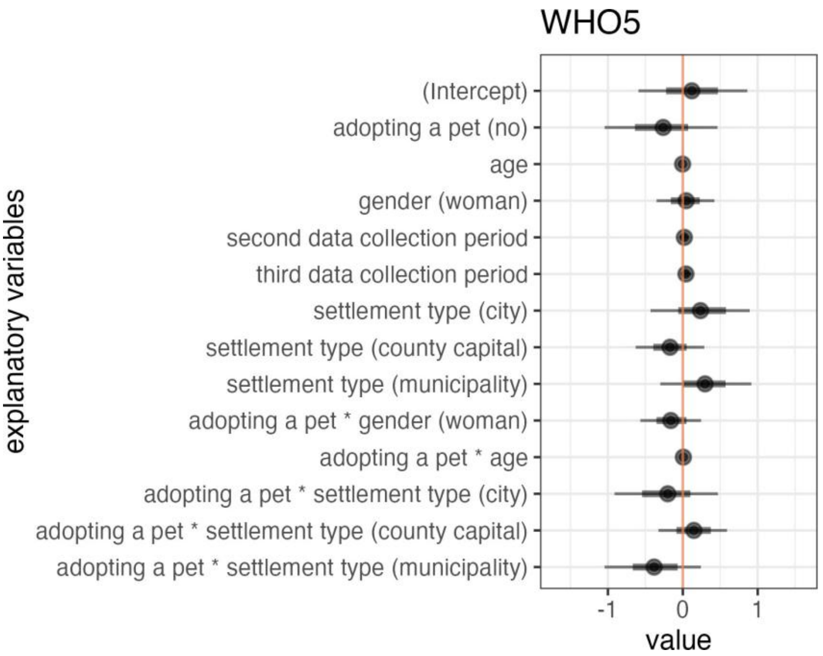
WHO5
(Intercept)
adopting a pet (no)
age
gender (woman)
second data collection period
third data collection period
settlement type (city)
settlement type (county capital)
settlement type (municipality)
adopting a pet * gender (woman)
adopting a pet * age
adopting a pet * settlement type (city)
adopting a pet * settlement type (county capital)
adopting a pet * settlement type (municipality)
explanatory variables
-1
0
1
value

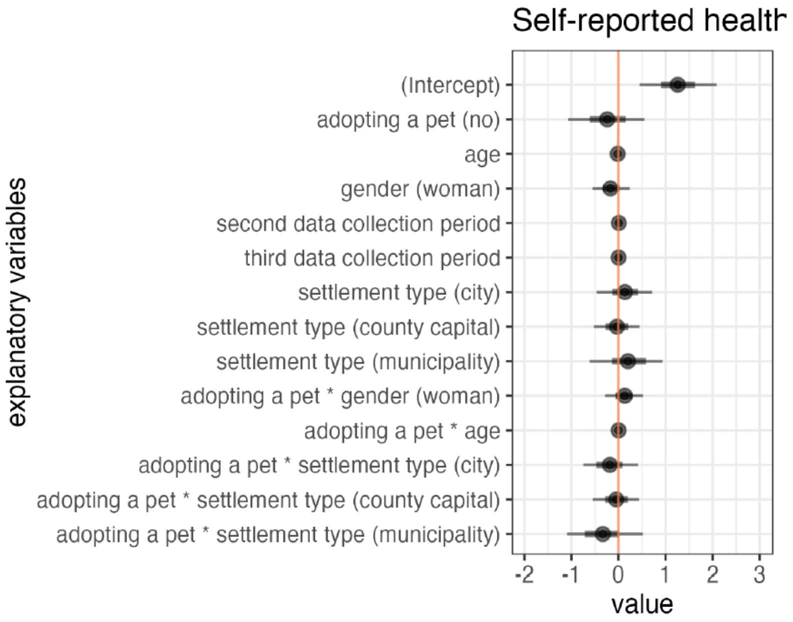
Self-reported health
explanatory variables
(Intercept)
adopting a pet (no)
age
gender (woman)
second data collection period
third data collection period
settlement type (city)
settlement type (county capital)
settlement type (municipality)
adopting a pet * gender (woman)
adopting a pet * age
adopting a pet * settlement type (city)
adopting a pet * settlement type (county capital)
adopting a pet * settlement type (municipality)
-2 -1 0 1 2 3
value

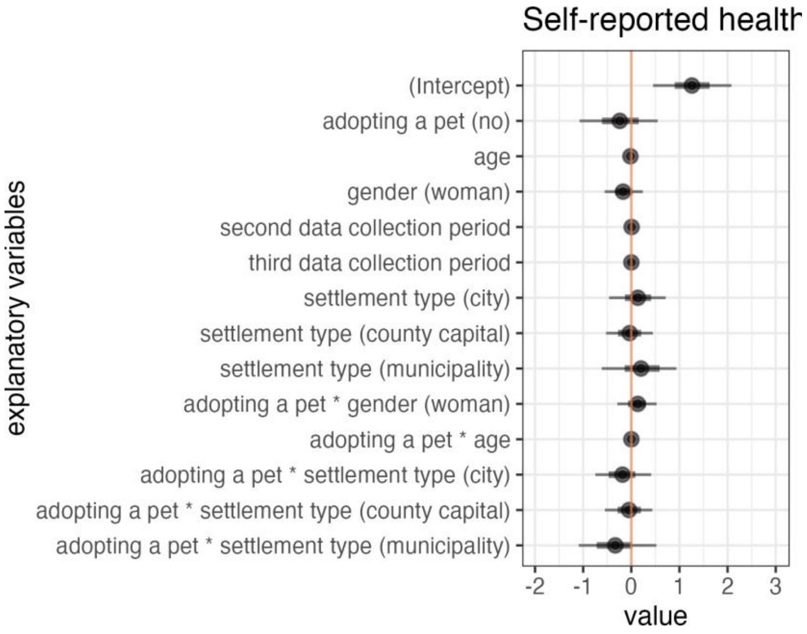
Self-reported health
explanatory variables
(Intercept)
adopting a pet (no)
age
gender (woman)
second data collection period
third data collection period
settlement type (city)
settlement type (county capital)
settlement type (municipality)
adopting a pet * gender (woman)
adopting a pet * age
adopting a pet * settlement type (city)
adopting a pet * settlement type (county capital)
adopting a pet * settlement type (municipality)
-2 -1 0 1 2 3
value

GLMM Series B

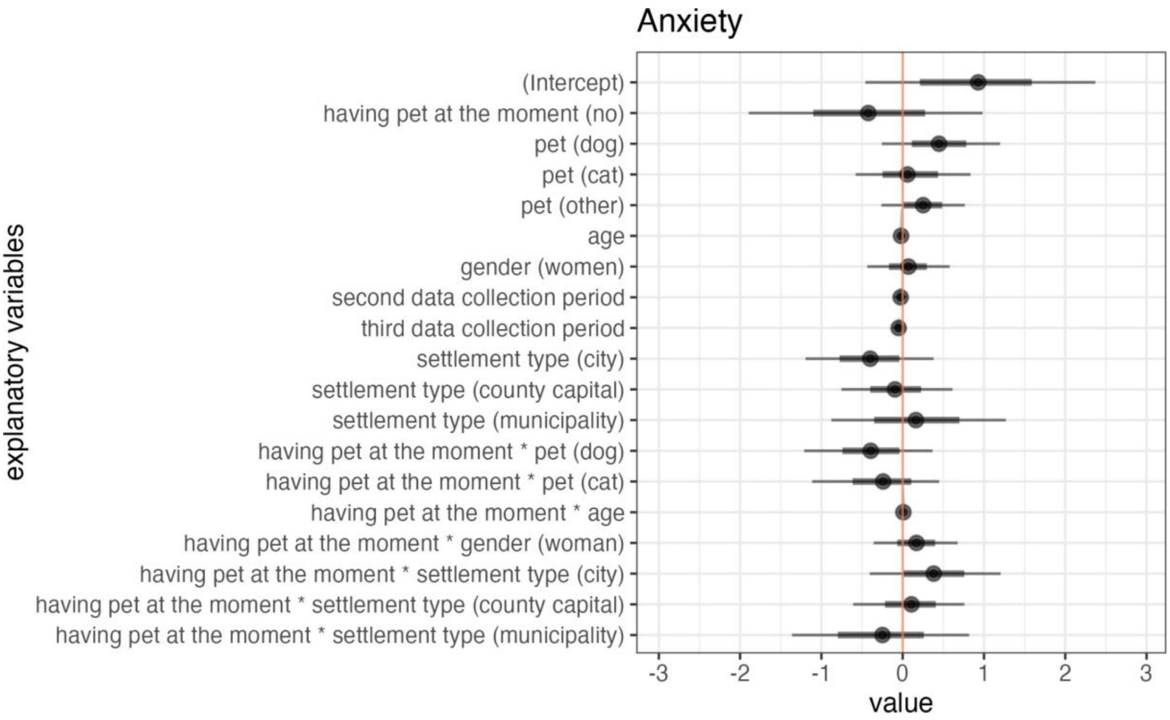

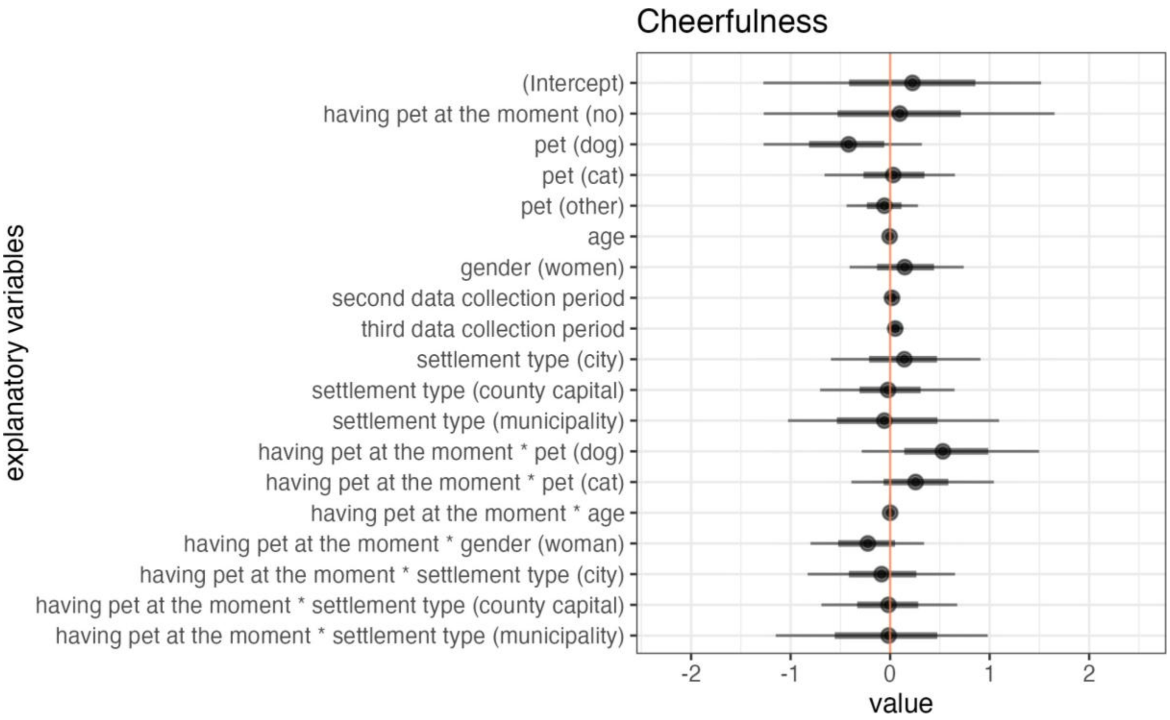

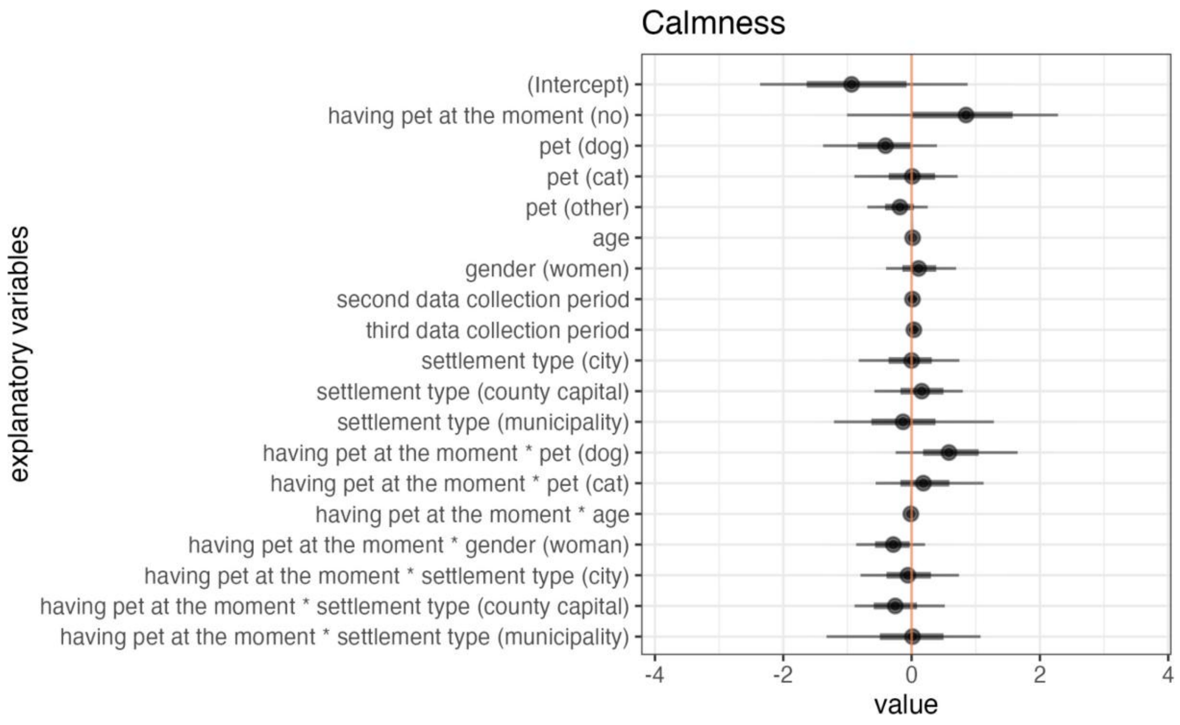
Calmness
(Intercept)
having pet at the moment (no)
pet (dog)
pet (cat)
pet (other)
age
gender (women)
second data collection period
third data collection period
settlement type (city)
settlement type (county capital)
settlement type (municipality)
having pet at the moment * pet (dog)
having pet at the moment * pet (cat)
having pet at the moment * age
having pet at the moment * gender (woman)
having pet at the moment * settlement type (city)
having pet at the moment * settlement type (county capital)
having pet at the moment * settlement type (municipality)
explanatory variables
-4
-2
0
2
4
value

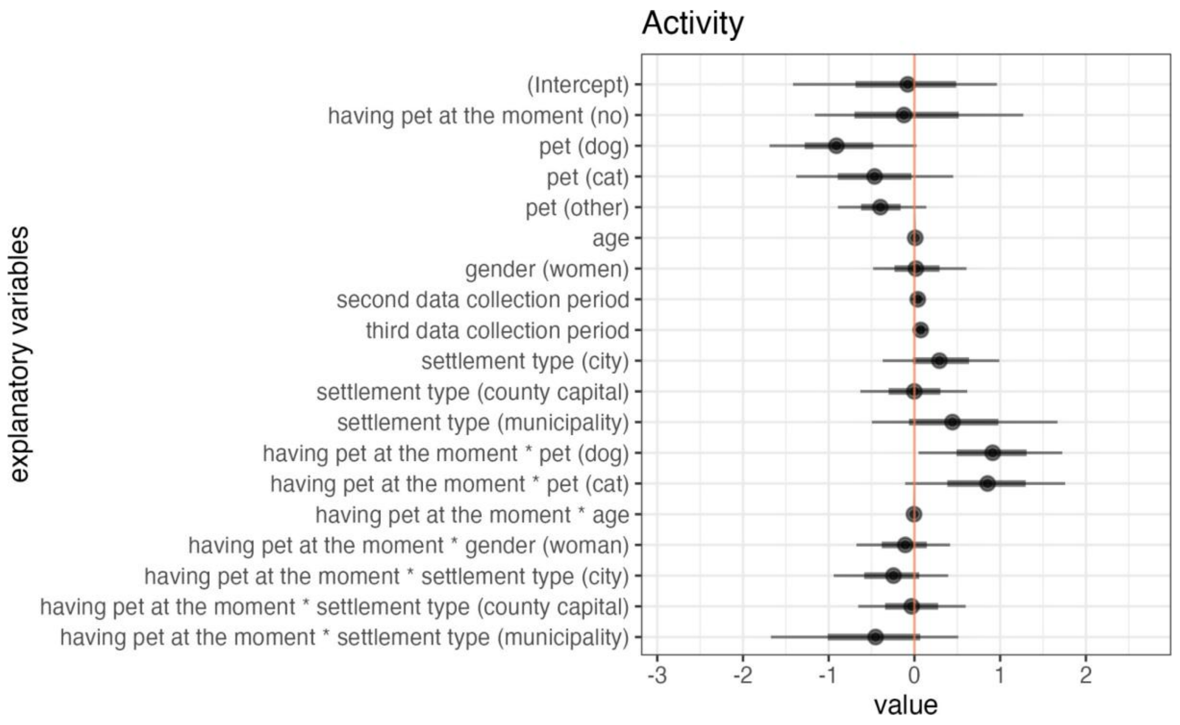
Activity
(Intercept)
having pet at the moment (no)
pet (dog)
pet (cat)
pet (other)
age
gender (women)
second data collection period
third data collection period
settlement type (city)
settlement type (county capital)
settlement type (municipality)
having pet at the moment * pet (dog)
having pet at the moment * pet (cat)
having pet at the moment * age
having pet at the moment * gender (woman)
having pet at the moment * settlement type (city)
having pet at the moment * settlement type (county capital)
having pet at the moment * settlement type (municipality)
explanatory variables
-3
-2
-1
0
1
2
value

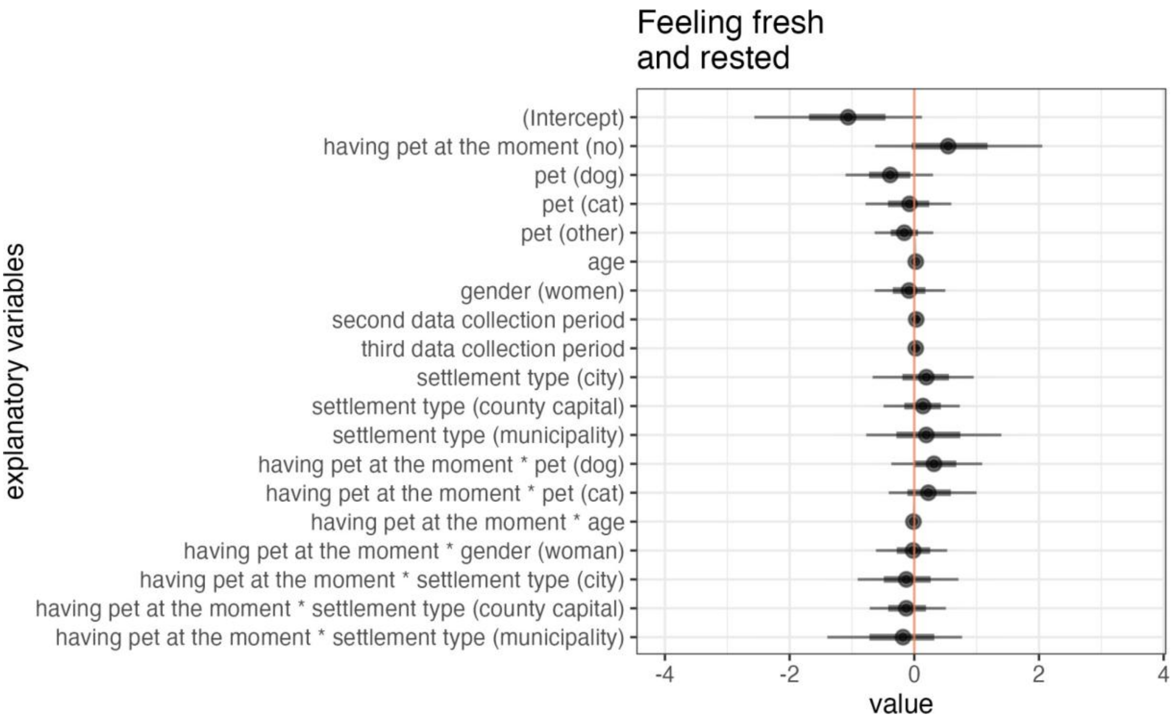
Feeling fresh and rested
(Intercept)
having pet at the moment (no)
pet (dog)
pet (cat)
pet (other)
age
gender (women)
second data collection period
third data collection period
settlement type (city)
settlement type (county capital)
settlement type (municipality)
having pet at the moment * pet (dog)
having pet at the moment * pet (cat)
having pet at the moment * age
having pet at the moment * gender (woman)
having pet at the moment * settlement type (city)
having pet at the moment * settlement type (county capital)
having pet at the moment * settlement type (municipality)
explanatory variables
-4
-2
0
2
4
value

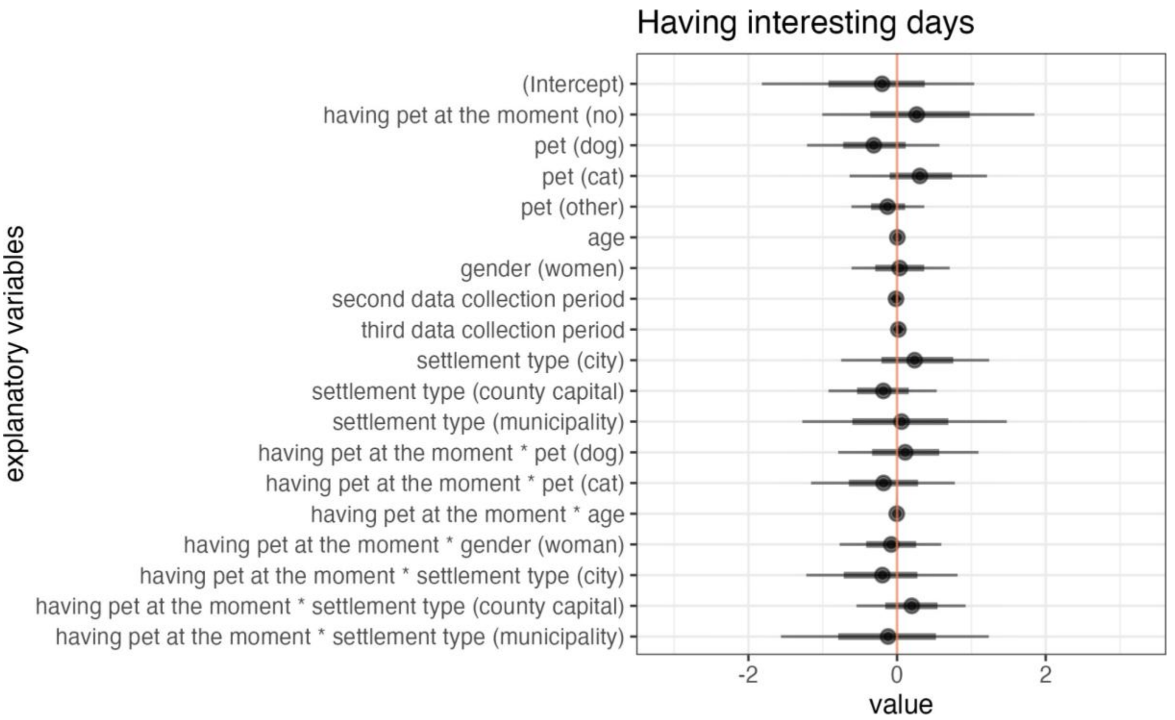
Having interesting days
(Intercept)
having pet at the moment (no)
pet (dog)
pet (cat)
pet (other)
age
gender (women)
second data collection period
third data collection period
settlement type (city)
settlement type (county capital)
settlement type (municipality)
having pet at the moment * pet (dog)
having pet at the moment * pet (cat)
having pet at the moment * age
having pet at the moment * gender (woman)
having pet at the moment * settlement type (city)
having pet at the moment * settlement type (county capital)
having pet at the moment * settlement type (municipality)
explanatory variables
-2
0
2
value

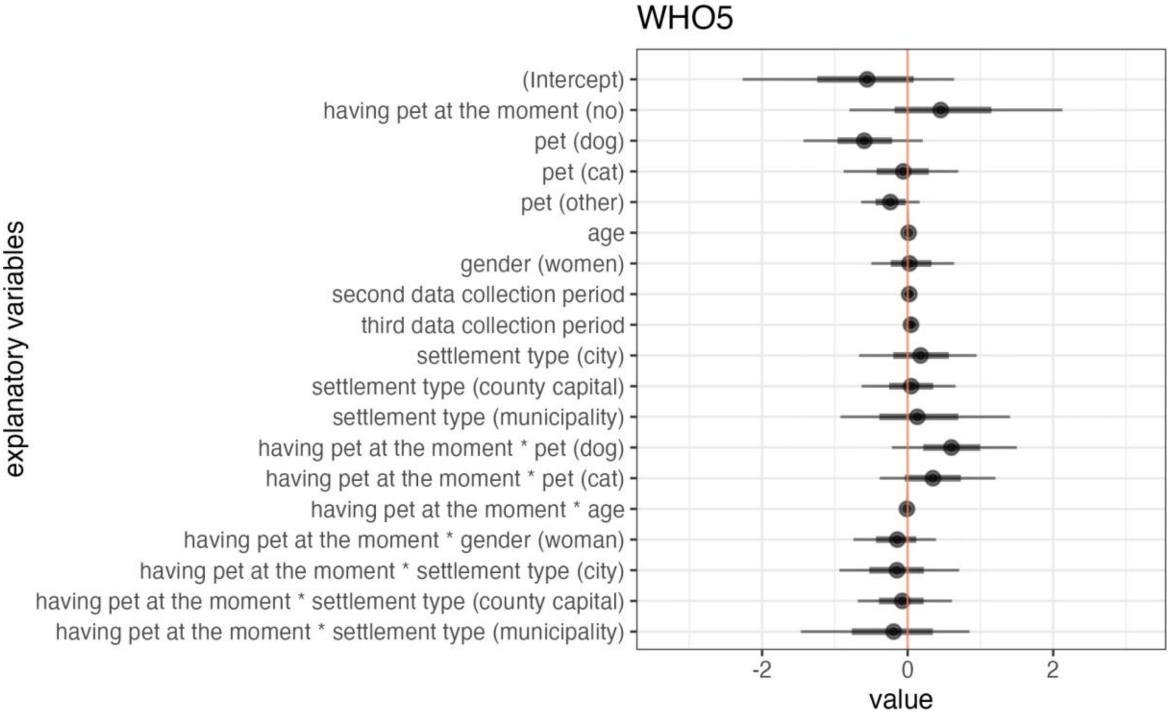
WHO5
(Intercept)
having pet at the moment (no)
pet (dog)
pet (cat)
pet (other)
age
gender (women)
second data collection period
third data collection period
settlement type (city)
settlement type (county capital)
settlement type (municipality)
having pet at the moment * pet (dog)
having pet at the moment * pet (cat)
having pet at the moment * age
having pet at the moment * gender (woman)
having pet at the moment * settlement type (city)
having pet at the moment * settlement type (county capital)
having pet at the moment * settlement type (municipality)
explanatory variables
-2
0
2
value

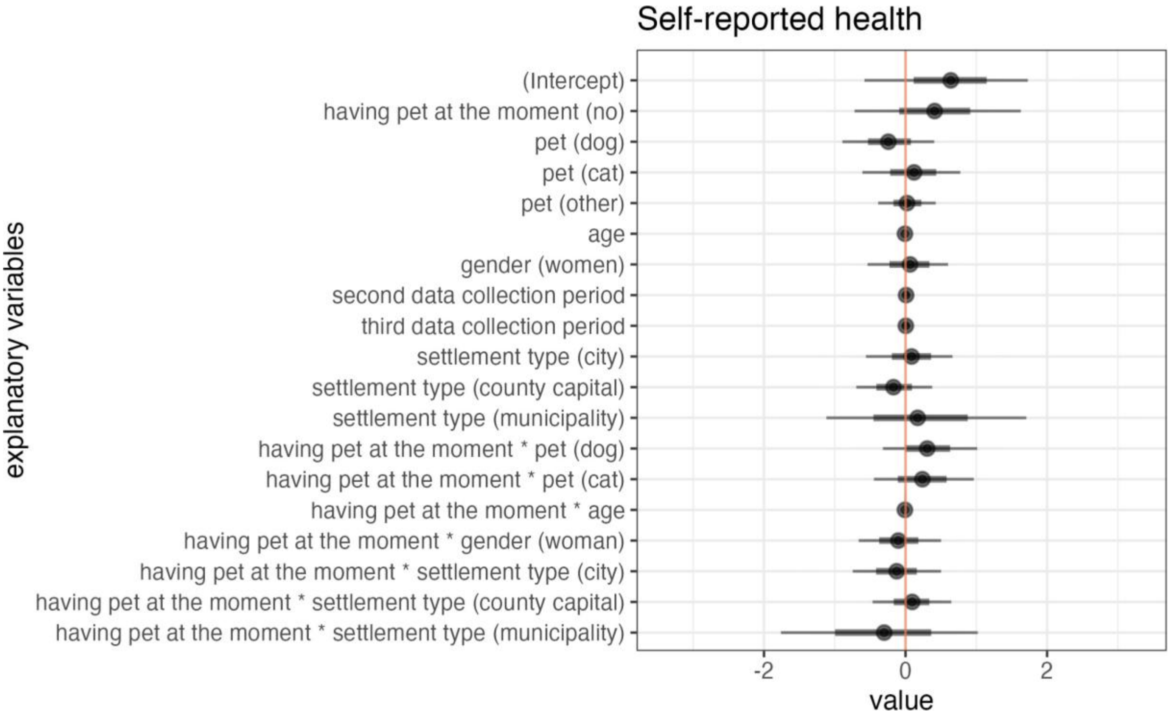
Self-reported health
(Intercept)
having pet at the moment (no)
pet (dog)
pet (cat)
pet (other)
age
gender (women)
second data collection period
third data collection period
settlement type (city)
settlement type (county capital)
settlement type (municipality)
having pet at the moment * pet (dog)
having pet at the moment * pet (cat)
having pet at the moment * age
having pet at the moment * gender (woman)
having pet at the moment * settlement type (city)
having pet at the moment * settlement type (county capital)
having pet at the moment * settlement type (municipality)
explanatory variables
-2
0
2
value

GLMM series C

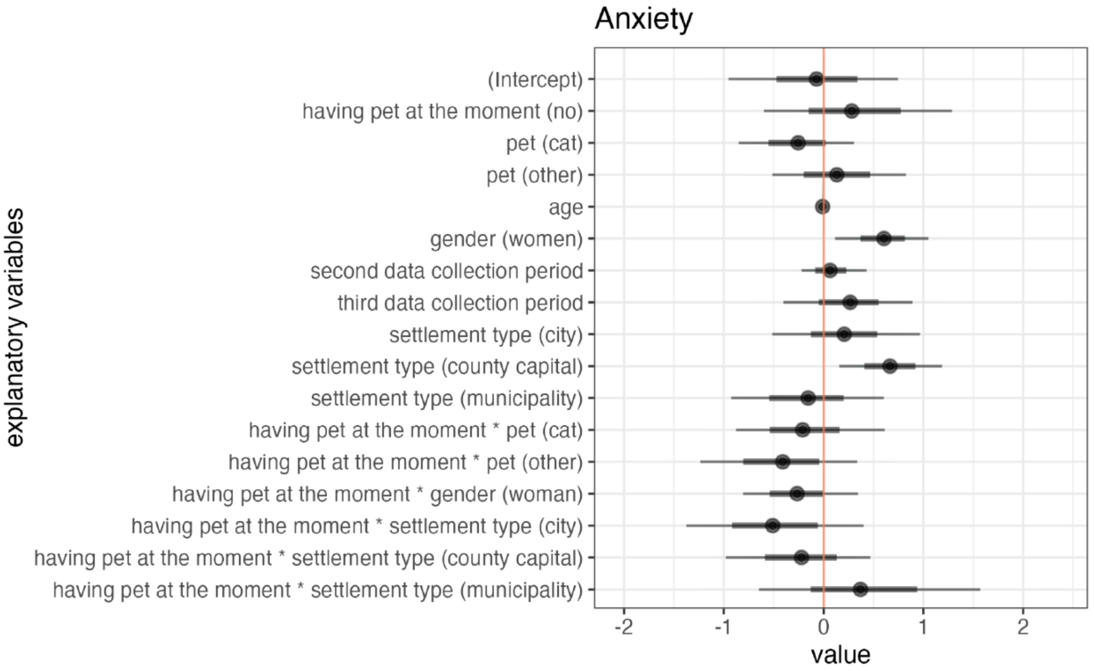

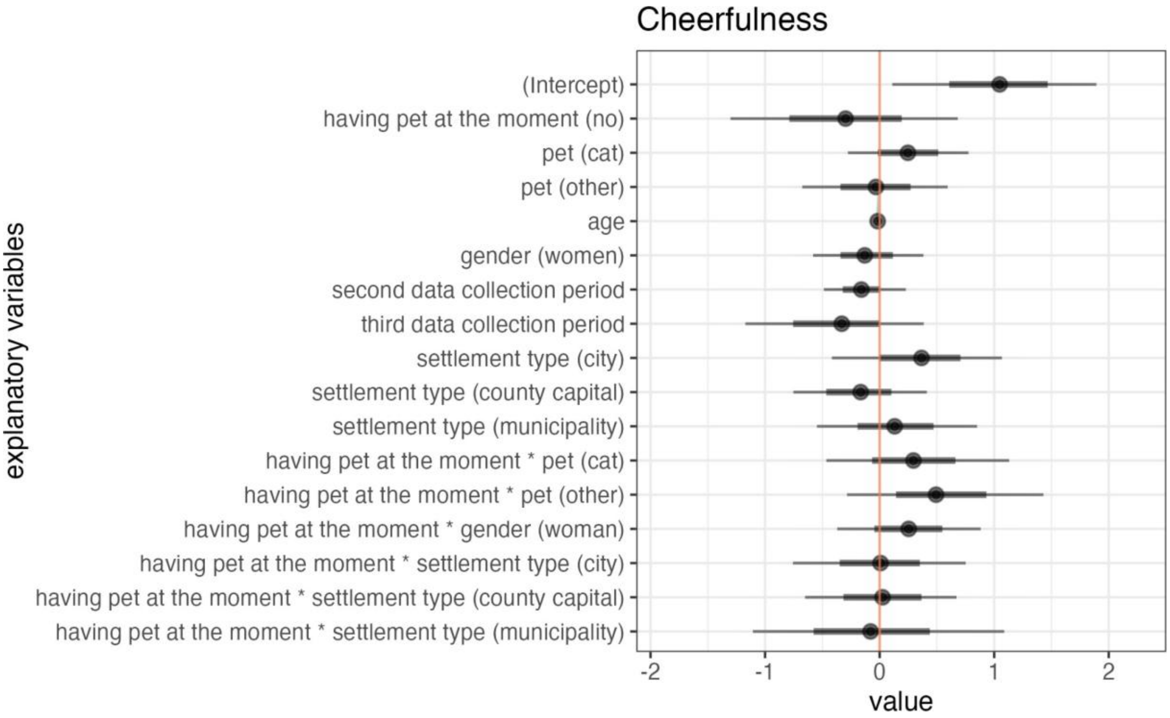

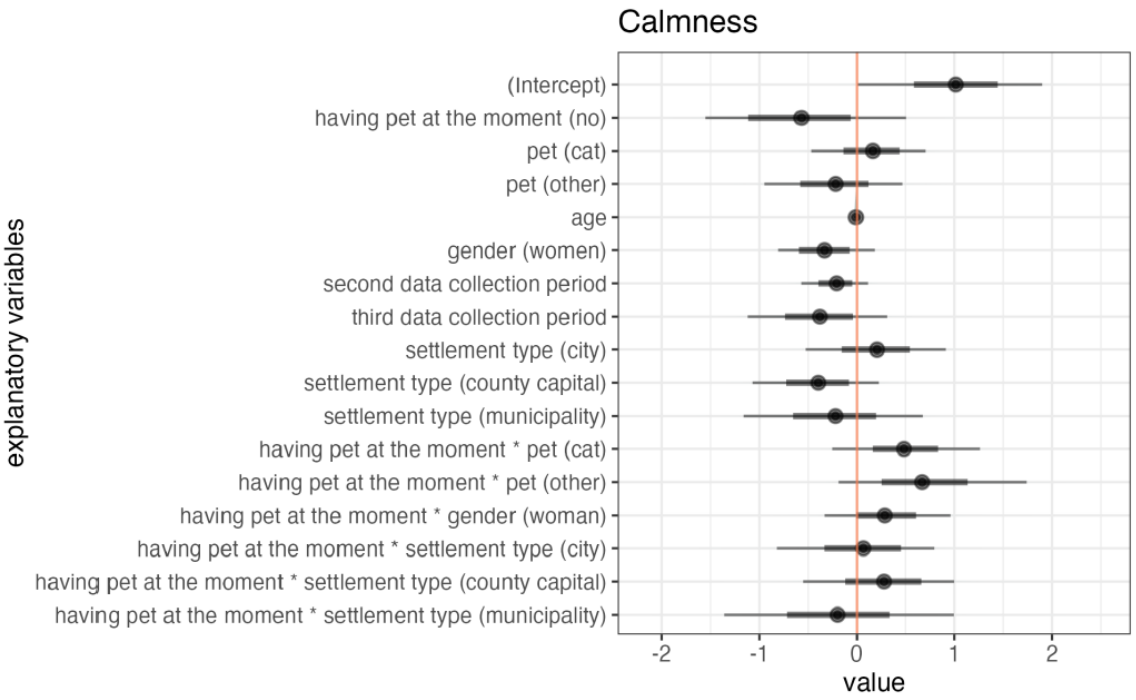
Calmness
(Intercept)
having pet at the moment (no)
pet (cat)
pet (other)
age
gender (women)
second data collection period
third data collection period
settlement type (city)
settlement type (county capital)
settlement type (municipality)
having pet at the moment * pet (cat)
having pet at the moment * pet (other)
having pet at the moment * gender (woman)
having pet at the moment * settlement type (city)
having pet at the moment * settlement type (county capital)
having pet at the moment * settlement type (municipality)
explanatory variables
-2
-1
0
1
2
value

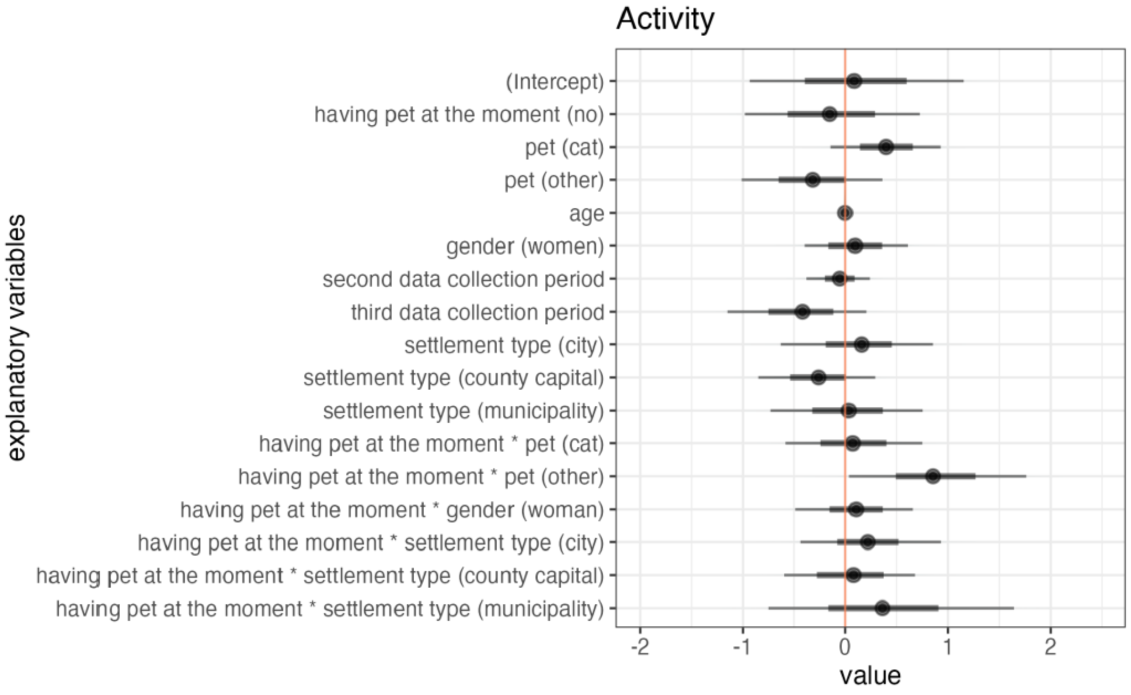
Activity
(Intercept)
having pet at the moment (no)
pet (cat)
pet (other)
age
gender (women)
second data collection period
third data collection period
settlement type (city)
settlement type (county capital)
settlement type (municipality)
having pet at the moment * pet (cat)
having pet at the moment * pet (other)
having pet at the moment * gender (woman)
having pet at the moment * settlement type (city)
having pet at the moment * settlement type (county capital)
having pet at the moment * settlement type (municipality)
explanatory variables
-2
-1
0
1
2
value

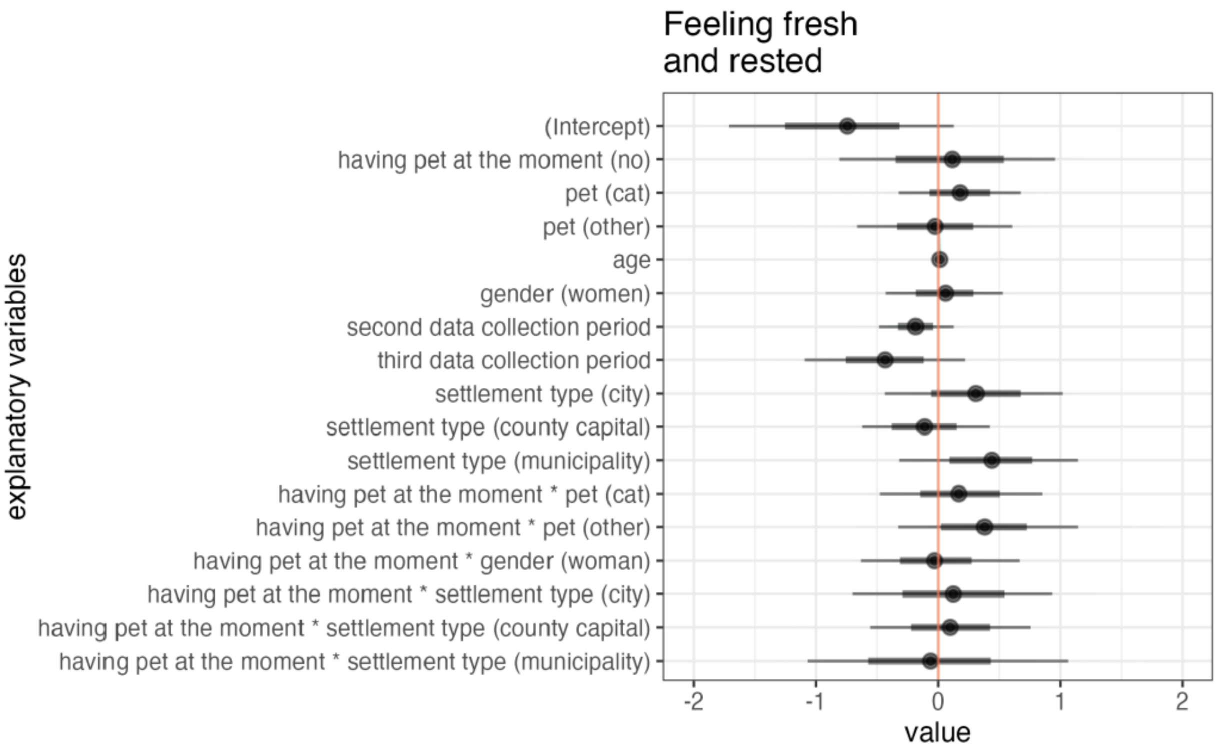
Feeling fresh and rested
(Intercept)
having pet at the moment (no)
pet (cat)
pet (other)
age
gender (women)
second data collection period
third data collection period
settlement type (city)
settlement type (county capital)
settlement type (municipality)
having pet at the moment * pet (cat)
having pet at the moment * pet (other)
having pet at the moment * gender (woman)
having pet at the moment * settlement type (city)
having pet at the moment * settlement type (county capital)
having pet at the moment * settlement type (municipality)
explanatory variables
-2
-1
0
1
2
value

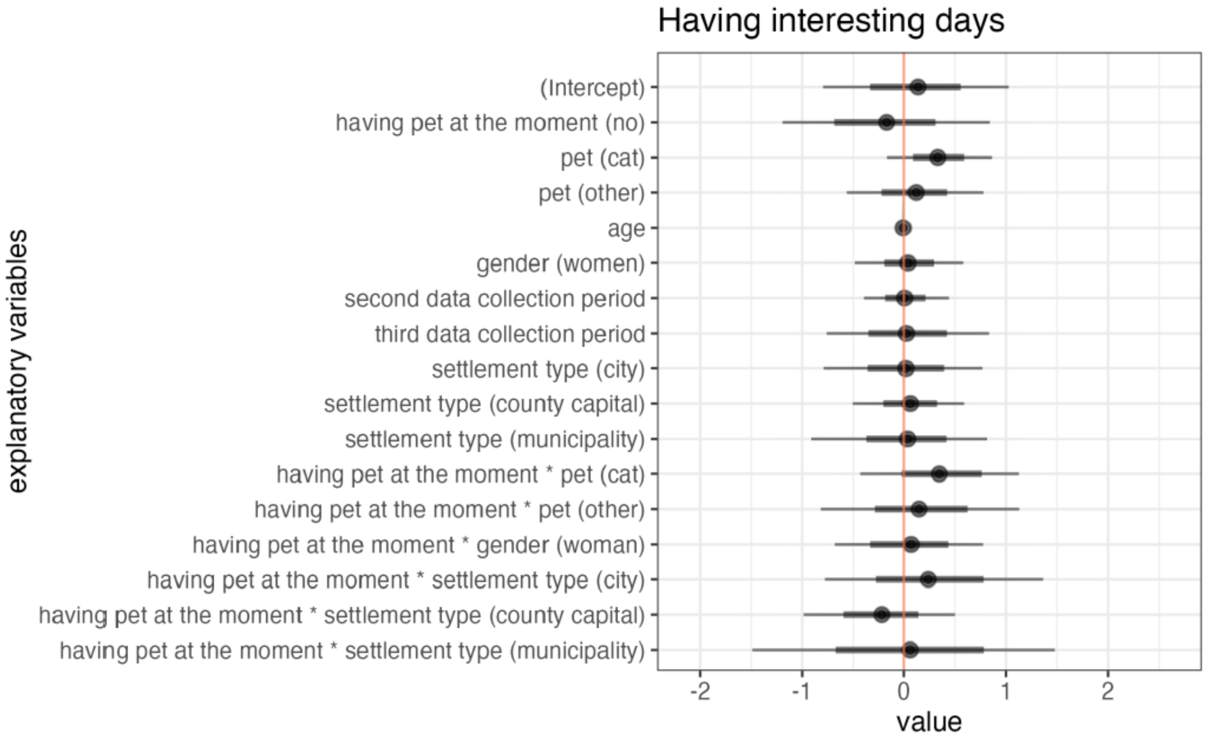
Having interesting days
(Intercept)
having pet at the moment (no)
pet (cat)
pet (other)
age
gender (women)
second data collection period
third data collection period
settlement type (city)
settlement type (county capital)
settlement type (municipality)
having pet at the moment * pet (cat)
having pet at the moment * pet (other)
having pet at the moment * gender (woman)
having pet at the moment * settlement type (city)
having pet at the moment * settlement type (county capital)
having pet at the moment * settlement type (municipality)
explanatory variables
-2
-1
0
1
2
value

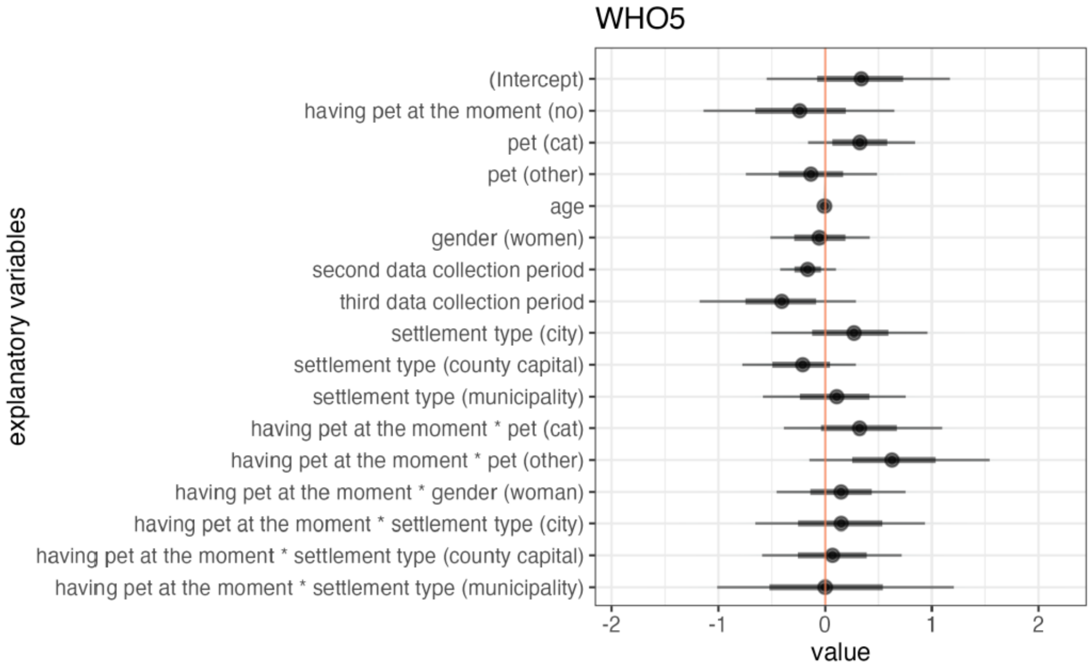
WHO5
(Intercept)
having pet at the moment (no)
pet (cat)
pet (other)
age
gender (women)
second data collection period
third data collection period
settlement type (city)
settlement type (county capital)
settlement type (municipality)
having pet at the moment * pet (cat)
having pet at the moment * pet (other)
having pet at the moment * gender (woman)
having pet at the moment * settlement type (city)
having pet at the moment * settlement type (county capital)
having pet at the moment * settlement type (municipality)
explanatory variables
-2
-1
0
1
2
value

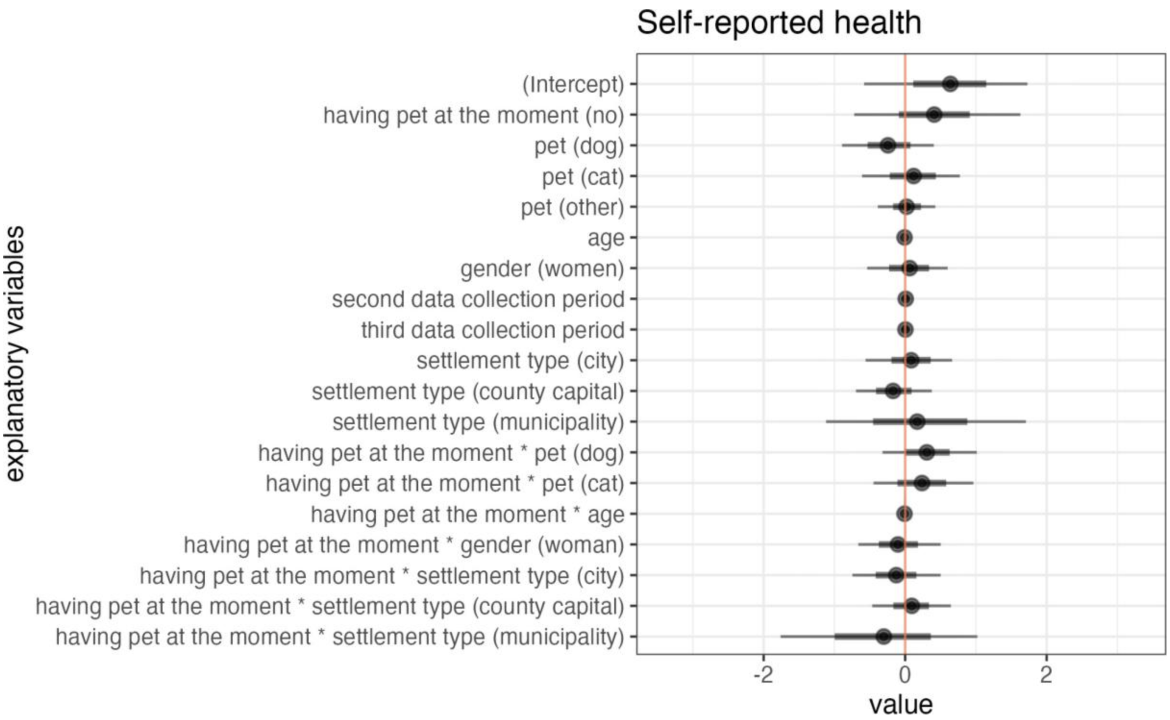
Self-reported health
(Intercept)
having pet at the moment (no)
pet (dog)
pet (cat)
pet (other)
age
gender (women)
second data collection period
third data collection period
settlement type (city)
settlement type (county capital)
settlement type (municipality)
having pet at the moment * pet (dog)
having pet at the moment * pet (cat)
having pet at the moment * age
having pet at the moment * gender (woman)
having pet at the moment * settlement type (city)
having pet at the moment * settlement type (county capital)
having pet at the moment * settlement type (municipality)
explanatory variables
-2
0
2
value

GLMM series D

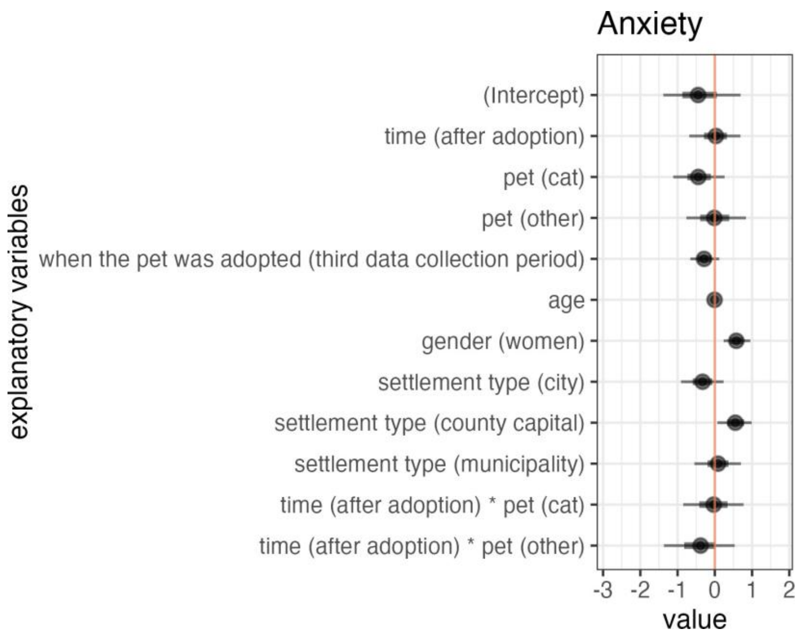

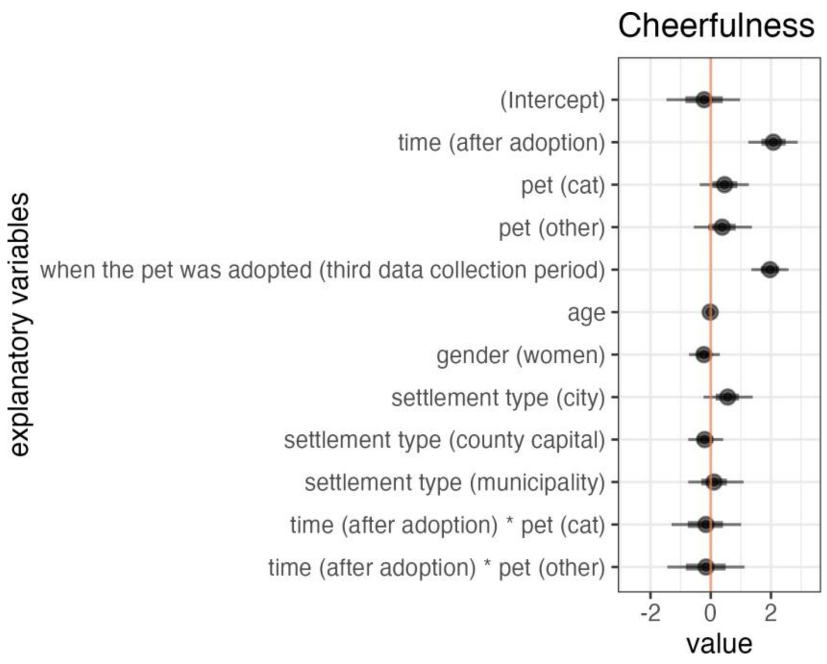

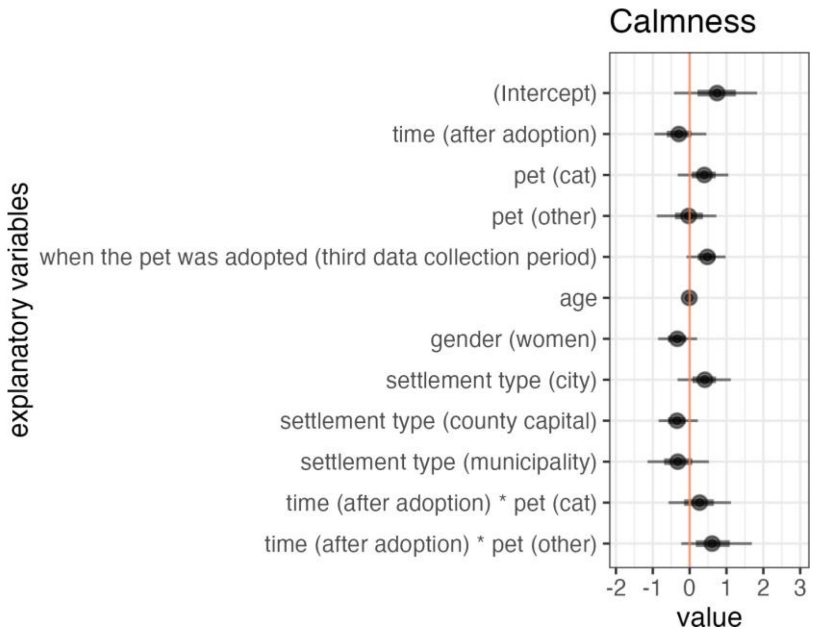
Calmness
(Intercept)
time (after adoption)
pet (cat)
pet (other)
when the pet was adopted (third data collection period)
age
gender (women)
settlement type (city)
settlement type (county capital)
settlement type (municipality)
time (after adoption) * pet (cat)
time (after adoption) * pet (other)
explanatory variables
-2 -1 0 1 2 3
value

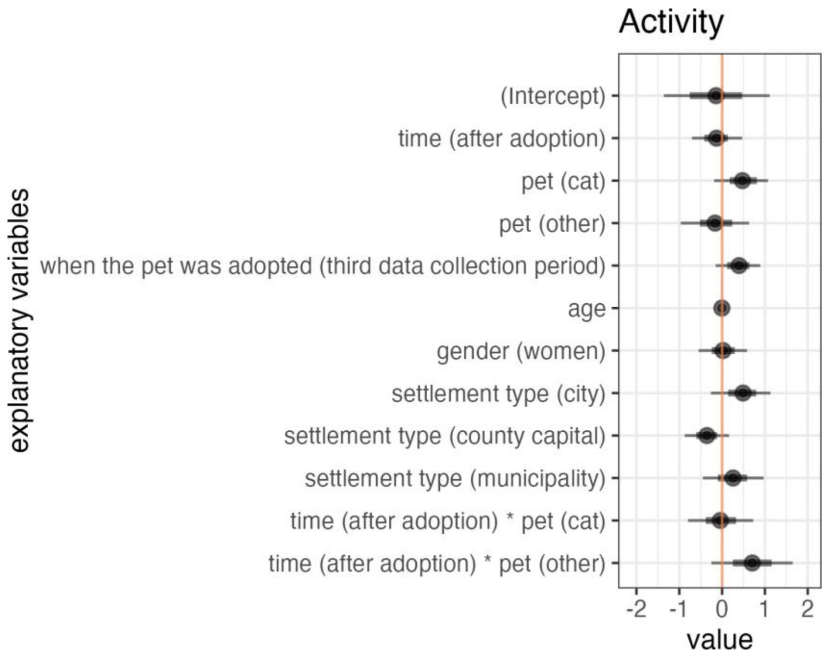
Activity
(Intercept)
time (after adoption)
pet (cat)
pet (other)
when the pet was adopted (third data collection period)
age
gender (women)
settlement type (city)
settlement type (county capital)
settlement type (municipality)
time (after adoption) * pet (cat)
time (after adoption) * pet (other)
explanatory variables
-2 -1 0 1 2
value

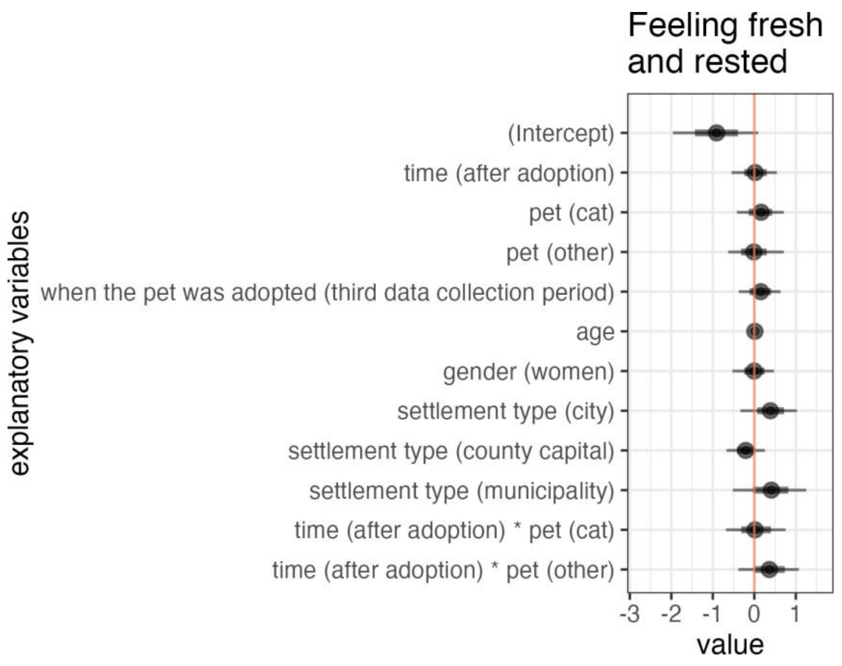
Feeling fresh and rested
explanatory variables
(Intercept)
time (after adoption)
pet (cat)
pet (other)
when the pet was adopted (third data collection period)
age
gender (women)
settlement type (city)
settlement type (county capital)
settlement type (municipality)
time (after adoption) * pet (cat)
time (after adoption) * pet (other)
-3 -2 -1 0 1
value

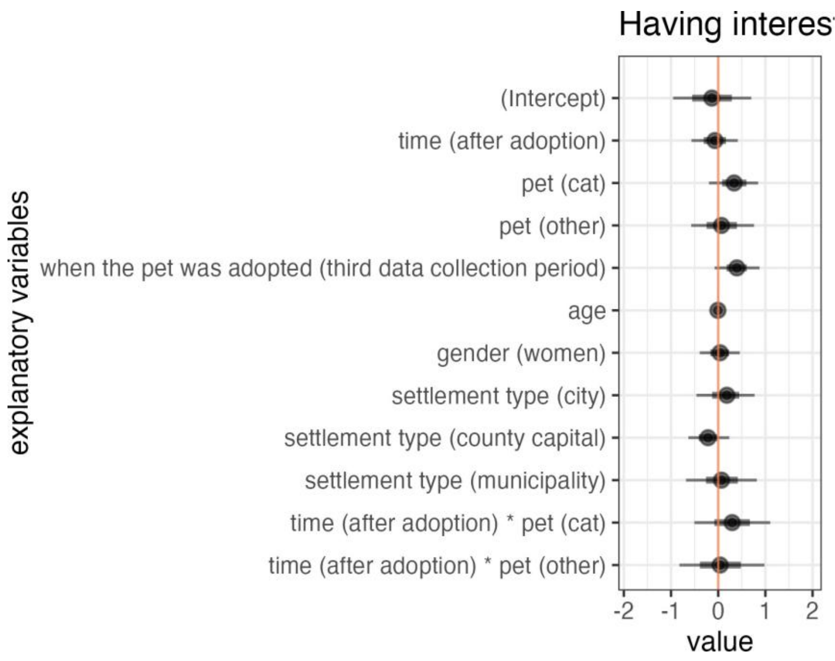
Having interes
explanatory variables
(Intercept)
time (after adoption)
pet (cat)
pet (other)
when the pet was adopted (third data collection period)
age
gender (women)
settlement type (city)
settlement type (county capital)
settlement type (municipality)
time (after adoption) * pet (cat)
time (after adoption) * pet (other)
-2 -1 0 1 2
value

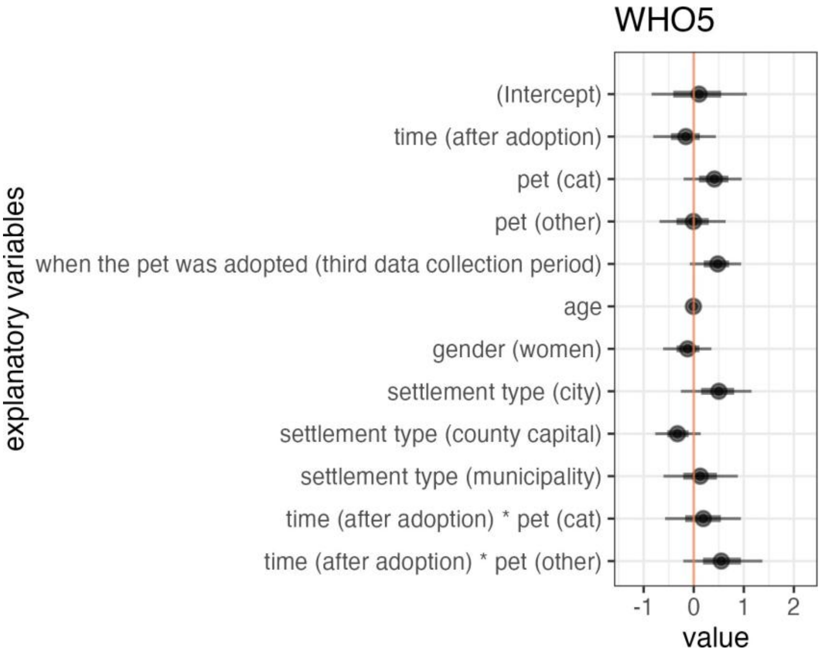
WHO5
explanatory variables
(Intercept)
time (after adoption)
pet (cat)
pet (other)
when the pet was adopted (third data collection period)
age
gender (women)
settlement type (city)
settlement type (county capital)
settlement type (municipality)
time (after adoption) * pet (cat)
time (after adoption) * pet (other)
-1
0
1
2
value

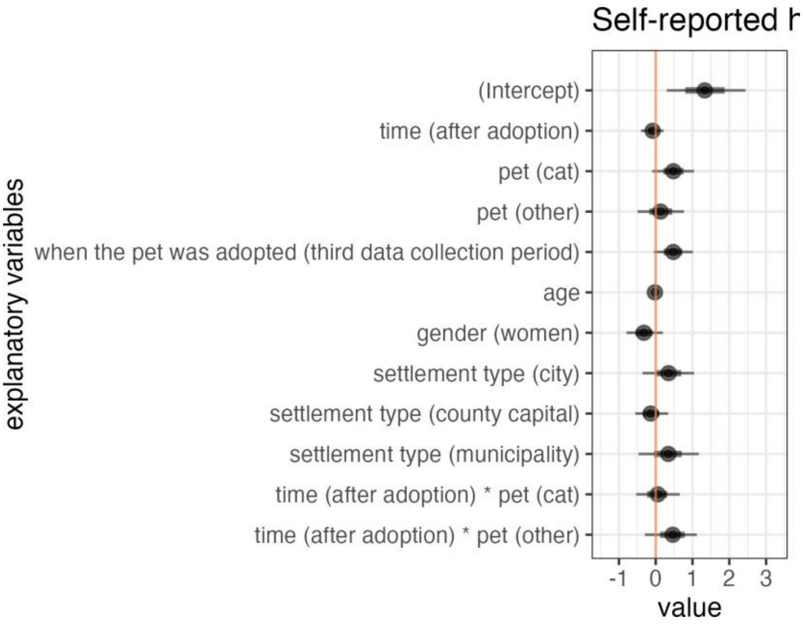
Self-reported h
explanatory variables
(Intercept)
time (after adoption)
pet (cat)
pet (other)
when the pet was adopted (third data collection period)
age
gender (women)
settlement type (city)
settlement type (county capital)
settlement type (municipality)
time (after adoption) * pet (cat)
time (after adoption) * pet (other)
-1
0
1
2
3
value

GLMM series E

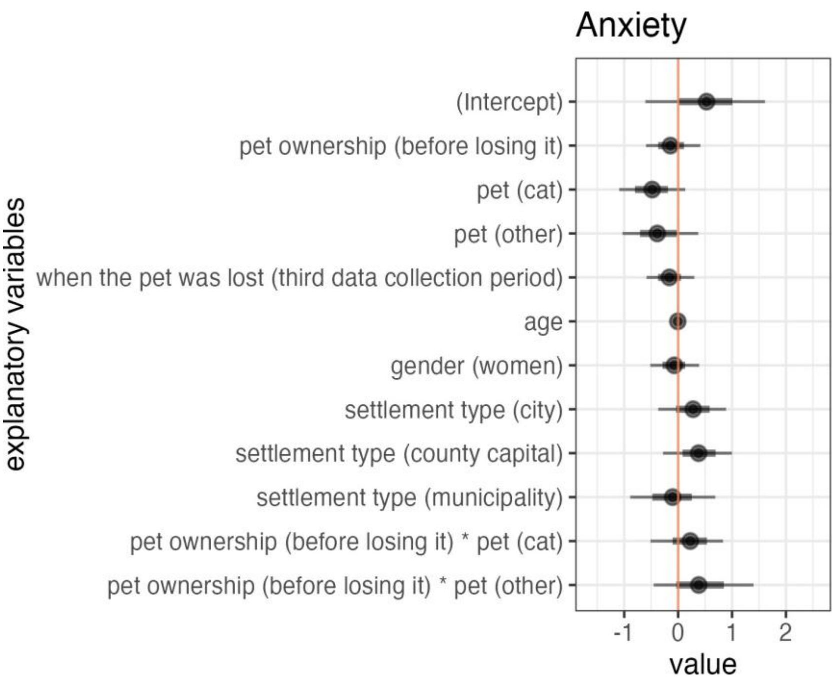

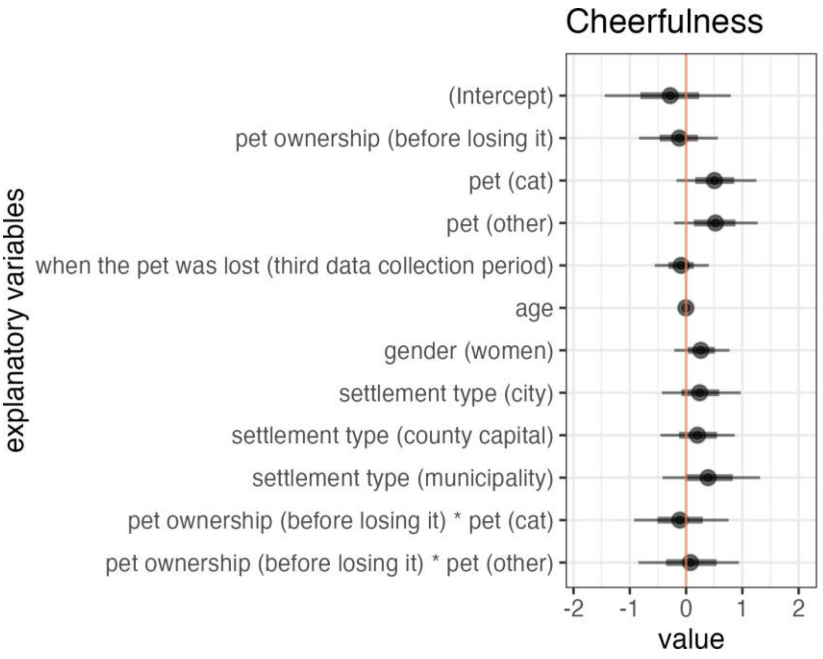

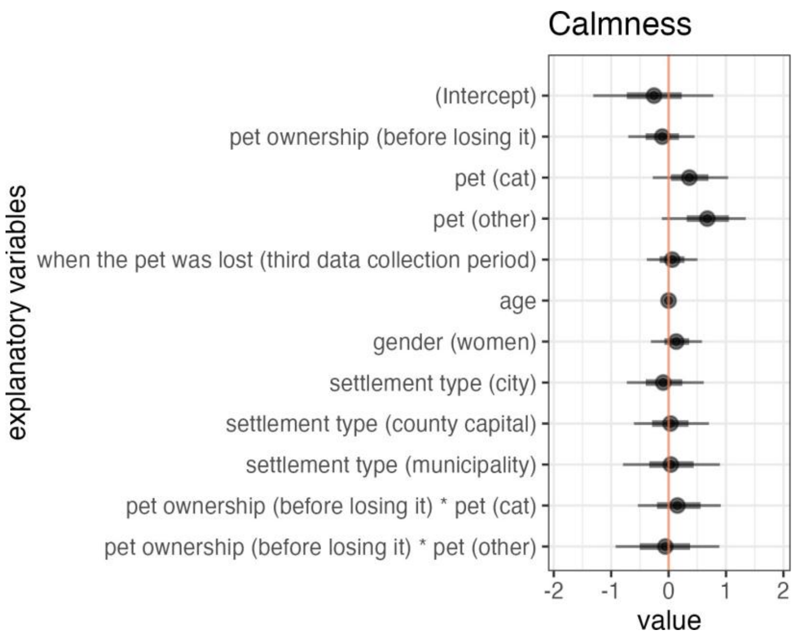
Calmness
explanatory variables
(Intercept)
pet ownership (before losing it)
pet (cat)
pet (other)
when the pet was lost (third data collection period)
age
gender (women)
settlement type (city)
settlement type (county capital)
settlement type (municipality)
pet ownership (before losing it) * pet (cat)
pet ownership (before losing it) * pet (other)
-2
-1
0
1
2
value

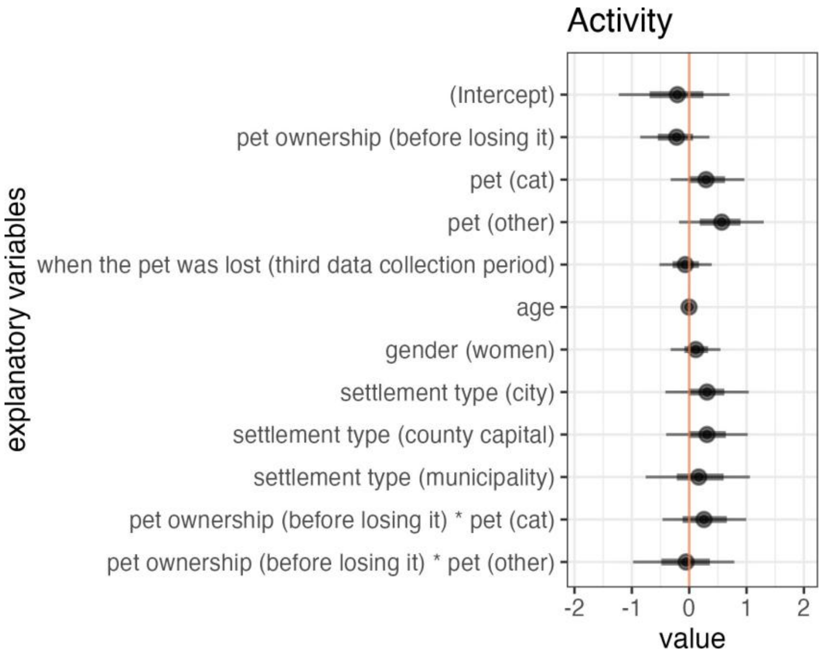
Activity
explanatory variables
(Intercept)
pet ownership (before losing it)
pet (cat)
pet (other)
when the pet was lost (third data collection period)
age
gender (women)
settlement type (city)
settlement type (county capital)
settlement type (municipality)
pet ownership (before losing it) * pet (cat)
pet ownership (before losing it) * pet (other)
-2
-1
0
1
2
value

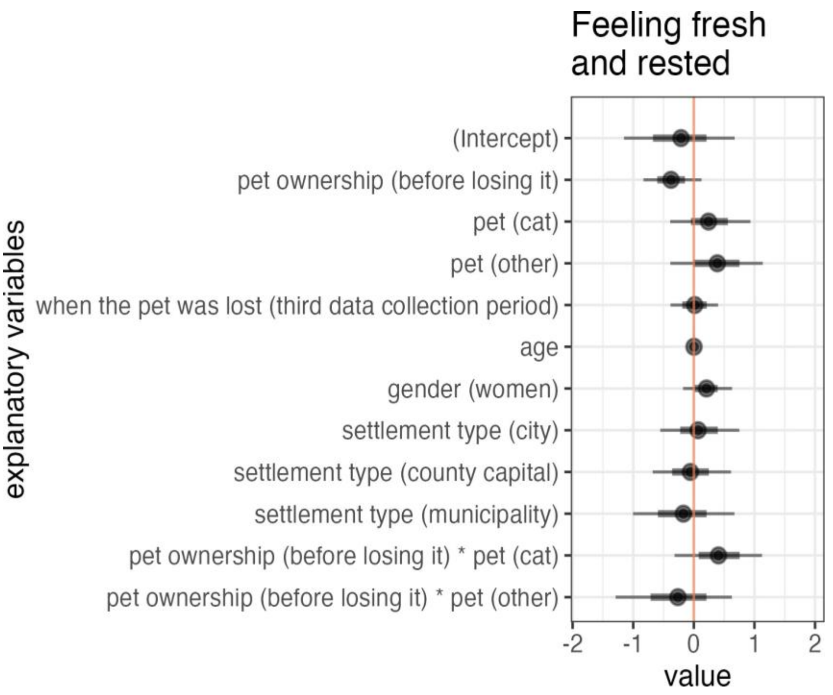
Feeling fresh and rested
(Intercept)
pet ownership (before losing it)
pet (cat)
pet (other)
when the pet was lost (third data collection period)
age
gender (women)
settlement type (city)
settlement type (county capital)
settlement type (municipality)
pet ownership (before losing it) * pet (cat)
pet ownership (before losing it) * pet (other)
explanatory variables
-2
-1
0
1
2
value

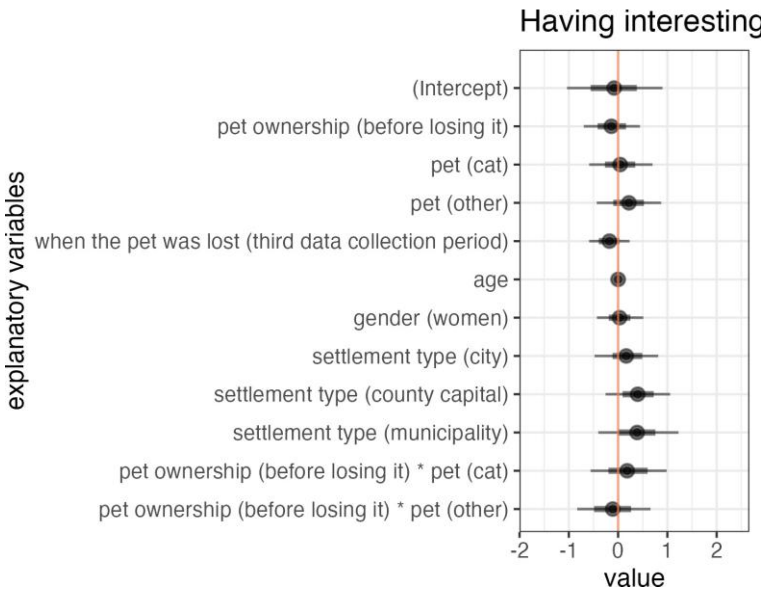
Having interesting
(Intercept)
pet ownership (before losing it)
pet (cat)
pet (other)
when the pet was lost (third data collection period)
age
gender (women)
settlement type (city)
settlement type (county capital)
settlement type (municipality)
pet ownership (before losing it) * pet (cat)
pet ownership (before losing it) * pet (other)
explanatory variables
-2
-1
0
1
2
value

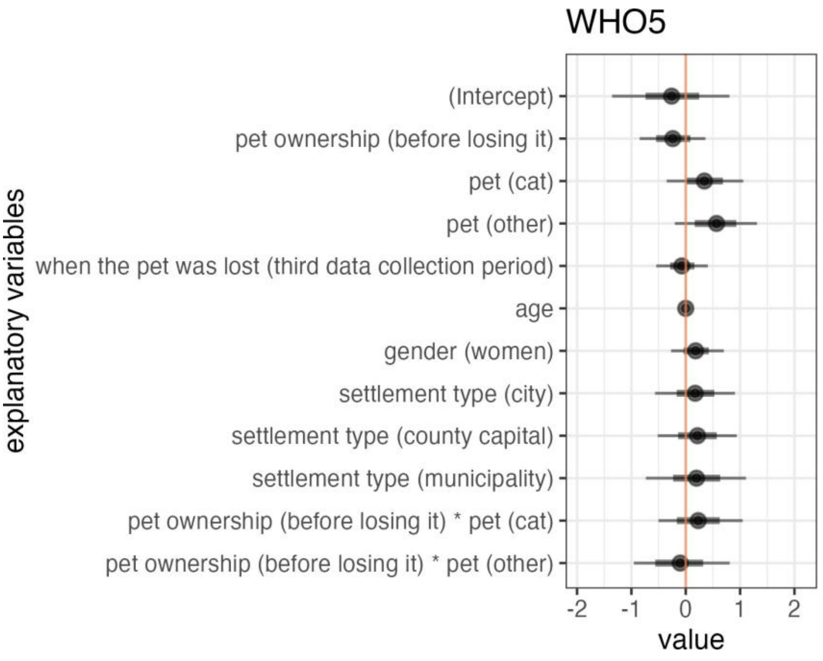

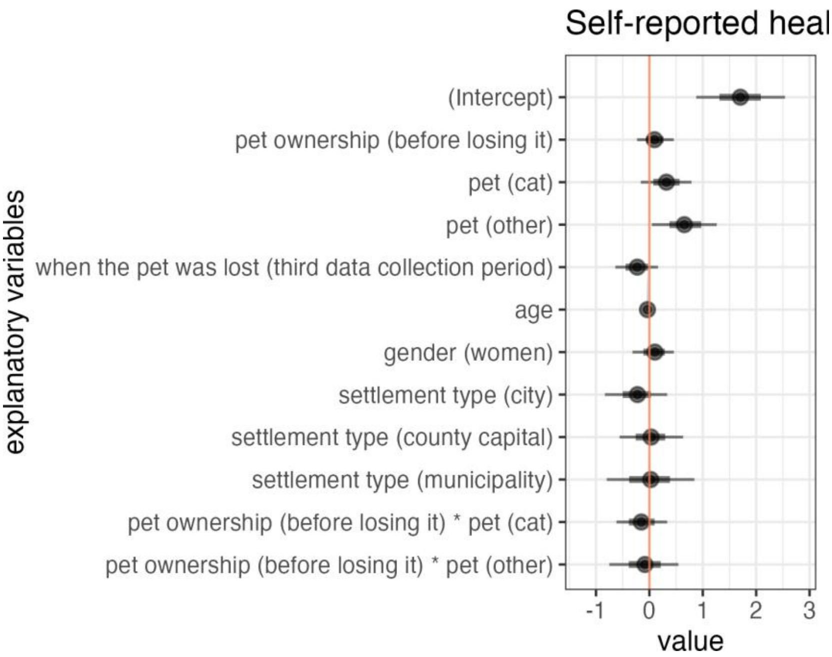

The effect of demographic variables

Age had a negative effect on cheerfulness (GLMM series A, C and D) and health (GLMM series A, C and D), and a positive effect on waking up fresh (GLMM series A and B; Figures S2, S4, S5). With age, anxiety decreased (GLMM series B; Figure S3).

Women were more anxious than men (GLMM series A, C and D; Figures S2, S4, S5).

Participants living in a county capital were sadder and more anxious than participants living in the capital city, Budapest (GLMM series A, C and D; Figures S2, S4, S5). Participants who adopted a pet and lived in a county capital were more anxious and sadder than participants who adopted a pet but lived in the capital city or did not adopt a pet.
